# Dynamical modelling of viral infection and cooperative immune protection in COVID-19 patients

**DOI:** 10.1371/journal.pcbi.1011383

**Published:** 2023-09-01

**Authors:** Zhengqing Zhou, Dianjie Li, Ziheng Zhao, Shuyu Shi, Jianghua Wu, Jianwei Li, Jingpeng Zhang, Ke Gui, Yu Zhang, Qi Ouyang, Heng Mei, Yu Hu, Fangting Li

**Affiliations:** 1 School of Physics, Center for Quantitative Biology, Peking University, Beijing, China; 2 Department of Immunology, School of Basic Medical Sciences, NHC Key Laboratory of Medical Immunology, Peking University, Beijing, China; 3 Peking University Third Hospital, Peking University, Beijing, China; 4 Institute of Hematology, Union Hospital, Tongji Medical College, Huazhong University of Science and Technology, Wuhan, China; Queensland University of Technology, AUSTRALIA

## Abstract

Once challenged by the SARS-CoV-2 virus, the human host immune system triggers a dynamic process against infection. We constructed a mathematical model to describe host innate and adaptive immune response to viral challenge. Based on the dynamic properties of viral load and immune response, we classified the resulting dynamics into four modes, reflecting increasing severity of COVID-19 disease. We found the numerical product of immune system’s ability to clear the virus and to kill the infected cells, namely immune efficacy, to be predictive of disease severity. We also investigated vaccine-induced protection against SARS-CoV-2 infection. Results suggested that immune efficacy based on memory T cells and neutralizing antibody titers could be used to predict population vaccine protection rates. Finally, we analyzed infection dynamics of SARS-CoV-2 variants within the construct of our mathematical model. Overall, our results provide a systematic framework for understanding the dynamics of host response upon challenge by SARS-CoV-2 infection, and this framework can be used to predict vaccine protection and perform clinical diagnosis.

## Author summary

Once challenged by the SARS-CoV-2 virus, the host immune system initiates a dynamic process against infection. Countless experimental, clinical, and theoretical studies have been carried out to understand the pathogenesis of SARS-CoV-2 infection, improve treatments or optimize vaccine strategies. Most of the time, people have been focusing on one arm of host immune system during SARS-CoV-2 infection and vaccine protection, despite there are three. A systematic understanding towards the innate, cellular, and humoral immunologic responses remains incomplete. Here, we report a mathematical model that captures the virus-immunity dynamics in both primary infection and vaccine-rendered protection. Mathematical analysis reveals the overall host immune system functions as an integrative dynamic metric combating SARS-CoV-2 infection. The metric reveals the cooperative nature of the immune system, especially between cellular and humoral immune responses. Numerical simulation and data analysis show this metric serves as a strong correlate for disease severity and vaccine protection rates against the original and variant SARS-CoV-2 strains. Our results put forth a systematic framework for understanding the virus-immune interaction during SARS-CoV-2 infection, which could be further deployed for treatment formulation and vaccine optimization.

## Introduction

Coronavirus disease 2019 (COVID-19) caused by the SARS-CoV-2 virus has spread globally with untold damage to global health, economy, and society. SARS-CoV-2 and its variants of concern (VOCs) have caused high morbidity and mortality among the unvaccinated, even escaping the protective immunity of neutralizing antibodies provided by mRNA-based and other vaccines. COVID-19 patients with weaker innate immunity, as manifested by lower HLA-II expression level [1], are more likely to show severe symptoms (~20%), including lymphopenia and cytokine release syndrome [2], otherwise known as “cytokine storm”, accompanied by elevation of the proinflammatory cytokine IL-6. COVID-19 patients exhibit a longer incubation period (4~12 days) in comparison to SARS patients (2~7 days) [3], calling for the establishment of different management and prevention protocols. In addition, upon symptom onset, the viral load of SARS patients is significantly lower than that observed in COVID-19 patients [4]. The IFN-I response is significantly lower in COVID-19 patients compared to patients with influenza. Collectively, this evidence suggests, in general, that SARS-CoV-2 induces both weak and delayed innate immune response in comparison to other viruses that infect the respiratory system. Various clinical trials [3,5–8] have been implemented, aimed at formulating vaccines and antiviral treatments for COVID-19 patients.

According to the COVID-19 vaccine tracker (https://covid19.trackvaccines.org/, last updated 2022/12/02), only 50 out of 242 vaccine candidates has been approved globally. Recent research has identified neutralizing antibody level as a correlate of protection [9–12] with the rate of protection varying from 50% to 95% [13], as a predictor of the temporal efficacy of vaccines and, thus, a guide for the development of future vaccines [13]. Despite global efforts to develop and popularize vaccines, SARS-CoV-2 is gradually mutating with the potential for eroding, or even collapsing, herd immunity hard-won through global vaccination programs. For example, the Alpha variant (B.1.1.7), which became dominant in the UK in late 2020, the Delta variant (B.1.617.2), dominant in the summer of 2021, and the newly emerged and highly infectious Omicron (B.1.1.529) all feature increased person-to-person transmissibility [14–16] and the increasing ability to evade protective immunological surveillance [16–20].

Countless experimental and clinical investigations have been carried out so far with the aim of understanding the pathogenesis of SARS-CoV-2 infection [1,21], treating the infected [22] and protecting the susceptible [23]. Mathematical modeling could be a useful tool for studying the development of viral infection. In the last decade of the twentieth century, mathematical modeling has been used for quantitative interpretation of clinical data in HIV, HBV, and HCV viral infection. As a result, our understanding of the dynamics of these viruses and the implementation of treatment strategies have significantly improved [24–28]. The modeling of influenza infection further investigated the function of innate and adaptive immunity [29,30]. Mathematical model has also successfully established the bi-stable outcome of HCV and LCMV infections [31]. In the context of the global COVID-19 pandemic, mathematical models have also been deployed to analyze the timing and efficacy of antiviral therapies [4,32], correlations between viral dynamics and both infectivity [33] and mortality [34], and the circulation of white blood cells between immune compartments [35]. However, a systematic approach toward understanding immunologic response in SARS-CoV-2 infection and vaccine protection is yet to be accomplished.

SARS-CoV-2 infection leads to heterogeneous infection progress. To understand the immune embedding of these variable disease outcomes, mathematical models have successfully associated innate immunity with disease onset and inflammation level [36–40], and T cell response with infection clearance [37,38]. However, due to the complexity of the immune system, it remains a challenge to conclude the existing evidence by a general principle that links to different immune elements and accounts for the heterogeneous infection outcomes. Moreover, in vaccinated patients, antibodies [11] and immune memory cells [41] also actively participate in combating the viral challenge. The construction of a model that includes the innate, cellular, and humoral immunity and immune memories will allow us to 1) unveil the general principle that dictates different disease outcomes, 2) investigate the relationship between vaccine immunogenicity and efficacy, and 3) sort out major immunological and epidemiological differences in populations at risk for SARS-CoV-2 and its variants.

We herein offer such a systematic approach and report the development of a mathematical model that captures virus-host immune dynamics in both infection and vaccine-protected states. More specifically, we studied the within-host infection process in a heterogeneous population. We studied how cellular and humoral memory cooperate to protect against SARS-CoV-2 infection, and we put forward a quantitative predictor of vaccine efficacy. Finally, we discussed how the infection process and vaccination outcome are affected by newly emerged SARS-CoV-2 VOCs.

## Results

### A virus-immunity network model provides a framework for understanding the dynamic processes of SARS-CoV-2 infection

For our purposes, an effective mathematical model should account for key immune elements that constitute human immune response against SARS-CoV-2 challenge. Its construction should allow us to 1) understand the different outcomes of SARS-CoV-2 infection based on individual immune response, 2) analyze the temporal dynamics of different immune elements and how they are orchestrated to clear the infection and minimize the pathology, and 3) formulate relevant treatment and vaccination strategies.

**Fig 1:**
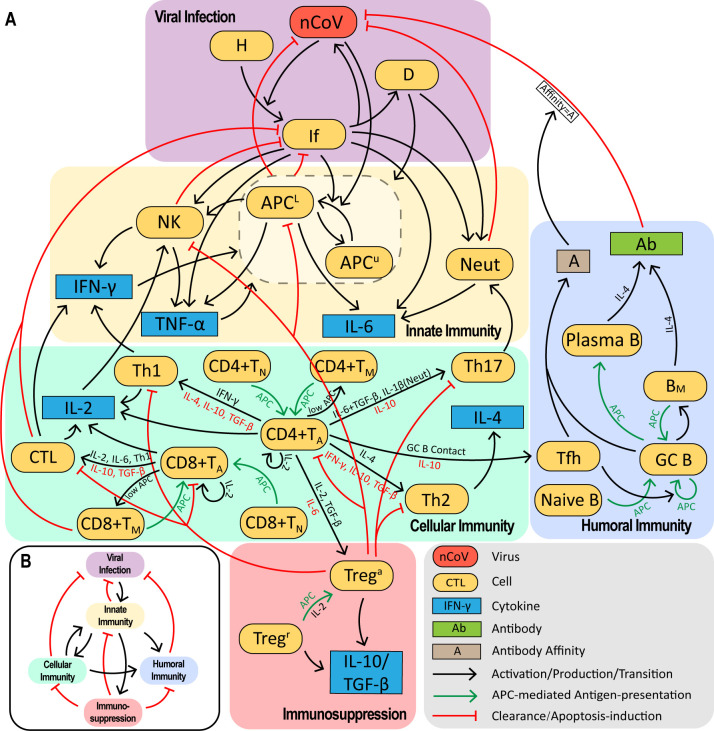
Immune system response network against SARS–CoV–2 infection. **(A)** The immune response network includes five modules: viral infection, innate immunity, cellular immunity, humoral immunity, and immunosuppression. Each module contains complex and nonlinear interactions among immune cells and cytokines. Black arrows represent activation, production or transition from one end to another, green arrows represent the transition process mediated by APC, and red arrows represent viral clearance and induced apoptosis. The cytokines along the arrow represent promotion (black) or repression (red) roles during lymphocyte activation and differentiation processes. **(B)** Overall interactions among the five modules.

In Fig 1, we constructed a virus-immunity interaction network consisting of a viral infection module, innate, cellular, and humoral immunity modules, and an immunosuppression module. In general, viral infection of target epithelial cells will be detected by innate immune cells like antigen-presenting cells (APC), natural killer cells (NK) and neutrophils (Neut). Through secretion of inflammatory cytokines and, more importantly, antigen presentation by APCs, innate immunity activates downstream adaptive immunity, including CD4+ and CD8+ T cells and B cells. These activated lymphocytes expand and carry out effector functions, working as helper T cells (Th), cytotoxic T cells (CTL) and plasma B cells (PB) secreting antibodies (Abs) to clear the virus and kill infected cells. Moreover, after infection, regulatory T cells (Treg) help downregulate the immune system, while some of these activated lymphocytes enter memory phase with latent ability to respond more rapidly upon reintroduction of the same pathogen. In particular, germinal center B cells (GC B) differentiate into PB, long-lived plasma cells that secret antibodies continuously, and memory B cells that can quickly respond to antigen by initiating secondary germinal center reaction [42]. For memory T cells, activated CD8+ T cells (CD8+T_A_) differentiate into effector memory T cells that exert cytotoxic functions, or differentiate into central memory T cells that can rapidly proliferate and differentiate into CD8+T_A_ in response to a “recognized” antigen. In short, memory CD4+ T cells (CD4+T_M_) can quickly differentiate into activated CD4+ T cells (CD4+T_A_) in response to antigen stimulation.

Type I Interferon (IFN-I) restricts viral replication and orchestrates innate and cellular immunity during viral infection [43]. SARS-CoV-2 have shown the remarkable ability to evade IFN-I [44,45]. The IFN-I level in COVID patients [1,46–49] is of an order of magnitude lower than the working concentration of IFN-I to protect target cells from SARS-CoV-2 infection [50–52], and two orders of magnitude below that of influenza virus infection [47]. While controversial studies have reported elevated [47,49] or reduced IFN-I levels [1] in severe infections, as these values are significantly below the working concentration, they should cause minimal difference in the immune dynamics (Section 1.3 in S1 Text). Therefore, we did not include the effects of IFN-I in our model.

For the sake of simplicity, our model was built according to the following blueprint. First, we focus on host immune response in a local infected area, such as lung and nearby draining lymph nodes. Second, multiple cytokines are correlated with inflammatory status [53], which herein is represented by our choice of IL-6 as the primary indicator of inflammation [2]. Third, we do not distinguish between the subsets of memory lymphocytes in our model, i.e., B_M_ represents both long-lived plasma cells and memory B cells; CD8+T_M_ represents both effector and central memory CD8+ T cells. Based on the network in Fig 1, we built a 32-variable ordinary differential equation (ODE) model to depict the dynamic processes of immune response against SARS-CoV-2 infection (Section 2 in S1 Text).

In the lung area, we define [*nCoV*] as the number density of free viral load, while [*H*] and [*If*] denote, respectively, healthy pulmonary epithelial cells and infected cells. The number density of neutrophils, antigen-loaded and unloaded APCs, NK cells and CTLs is respectively denoted as [*Neut*],[*APC*^*l*^], [*APC*^*u*^], [*NK*], and [*CTL*]. Similarly, [*CD*4+*T*_*M*_], [*CD*8+*T*_*M*_], and [*B*_*M*_] represent the number density of CD4+ and CD8+ T memory cells and B memory cells, respectively. [*Ab*] is neutralizing antibody titer. Viral load and lymphocytes are in units of 10^6^/mL, cytokines are in units of pg/mL, and antibodies are in units of μg/mL. More details about immune cells, cytokine dynamics and the ODE model can be found in Section 2 of S1 Text.

### Immune efficacy *ϵ* quantifies immune protection against viral infection

The immune system rallies immune cells, cytokines, chemokines and antibodies upon viral challenge. Immune response involves dozens of different types of immune cells and hundreds of functioning molecules. It remains a challenge to quantify the overall strength of host immune response and assess the relative importance of these elements. Here we show the derivation of an indicator for immune response strength against viral infection in our model.

In the viral infection module, the following equations show how SARS-CoV-2 virus infects lung epithelial cells:

$$\frac{d[nCoV]}{dt}=N_{1}d_{If}[If]-d_{v}\frac{\left[nCoV\right]}{K_{m}+[nCoV]}-\left\{f_{AntV}^{APC}k_{1}^{clear}[APC^{l}]+f_{AntV}^{APC}k_{2}^{clear}[APC^{u}]+k_{3}^{clear}[Neut]+k_{4}^{clear}A[Ab]\right\}[nCoV]$$ (1)

$$\frac{d[If]}{dt}=k_{infect}[nCoV][H]-d_{If}[If]-\left\{f_{AntV}^{APC}k_{1}^{kill}[APC^{l}]+f_{AntV}^{APC}k_{2}^{kill}[APC^{u}]+f_{eff}^{NK}k_{3}^{kill}[NK]+k_{4}^{kill}[CTL]+k_{5}^{kill}[CD8+T_{M}]\right\}[If]$$ (2)

$$\frac{d[H]}{dt}=r_{H}-k_{infect}[nCoV][H]-d_{H}[H]$$ (3)

The dynamics of viral infection is described in Eq 1. Virions are produced from infected cells at the rate of *N*_1_*d*_*If*_[*If*], where *N*_1_ is the burst size of SARS-CoV-2 virus (average number virions produced by a single infected cell), and *d*_*If*_ is the death rate of infected cells that release new virions. Virus is cleared by APCs, neutrophils and antibodies, together, at a combined rate of $\left\{f_{AntV}^{APC}k_{1}^{clear}[APC^{l}]+f_{AntV}^{APC}k_{2}^{clear}[APC^{u}]+k_{3}^{clear}[Neut]+k_{4}^{clear}A[Ab]\}[nCoV\right]$ and by mucosal immunity $d_{v}\frac{\left[nCoV\right]}{K_{m}+[nCoV]}$. In Eq 2, epithelial cells [*H*] are infected by free virions at rate *k*_*infect*_[*nCoV*][*H*] and turned into infected cells. Infected cells are killed by APCs, NK cells, CTLs and CD8+T_M_ cells at rate $\left\{f_{AntV}^{APC}k_{1}^{kill}[APC^{l}]+f_{AntV}^{APC}k_{2}^{kill}[APC^{u}]+f_{eff}^{NK}k_{3}^{kill}[NK]+k_{4}^{kill}[CTL]+k_{5}^{kill}[CD8+T_{M}]\}[If\right]$ and die at rate *d*_*If*_[*If*]. In Eq 3, healthy lung epithelial cells renew at rate *r*_*H*_ and undergo normal death at rate *d*_*H*_[*H*]. In the above, $f_{AntV}^{APC}$ and $f_{eff}^{NK}$ account for the augmentation of APC and NK effector function by cytokines and inflammatory signals, including IFN-γ for APC and IL-2 for NK. The affinity of antibodies to virions, named as *A*, increases in the presence of germinal center B cells (GC B) and T follicular helper cells (Tfh) along the course of disease, and plateaus when the infection is cleared and Tfh contracts (S4 Fig).

We define the infected cell killing rate as $\epsilon_{k}(t)\equiv {d_{If}+f}_{AntV}^{APC}k_{1}^{kill}[APC^{l}]+f_{AntV}^{APC}k_{2}^{kill}[APC^{u}]+f_{eff}^{NK}k_{3}^{kill}[NK]+k_{4}^{kill}[CTL]+k_{5}^{kill}[CD8+T_{M}]$, including virus- and immunity- mediated cell death, where the latter contributes to the majority of infected cell death when evoked. Similarly, the virus clearance rate is defined as *ϵ*_*c*_(*t*)+*ϵ*_*v*_(*t*), composing of mucosal immunity $\epsilon_{v}(t)=d_{v}\frac{1}{K_{m}+[nCoV]}$, and innate and humoral immune response to clear the virus $\epsilon_{c}(t)=f_{AntV}^{APC}k_{1}^{clear}[APC^{l}]+f_{AntV}^{APC}k_{2}^{clear}[APC^{u}]+k_{3}^{clear}[Neut]+k_{4}^{clear}A[Ab]$. When viral load is comparatively large, as in [*nCoV*]≫*K*_*m*_, *ϵ*_*v*_ goes to 0.

To obtain a concise dynamic equation of viral load, we set *d*[*If*]/*dt* = 0, and have $\frac{d[nCoV]}{dt}=(\varepsilon_{c}+\varepsilon_{v})(R_{t}-1)[nCoV]$. The viral reproduction number is defined as $R_{t}=\frac{\gamma }{(\varepsilon_{c}+\varepsilon_{v})\varepsilon_{k}}\frac{\left[H\right]}{[H{]}_{0}}$ [4,27]. *γ* ≡ *N*_1_*d*_*If*_*k*_*infect*_[*H*]_0_ stands for the maximum capability for the virus to replicate and [*H*]_0_ stands for the steady-state healthy pulmonary epithelial cell density. Assumption on pseudo steady state of viral load can also arrive at the same *R*_*t*_ (section 2.2 in S1 Text). Since we mainly focus on how the host immune system clears virus and kills infected cells, and *ϵ*_*v*_ goes to zero at high viral load, we define host immune efficacy as *ϵ*(*t*) ≡ *ϵ*_*c*_(*t*) *ϵ*_*k*_(*t*). Thus, the viral reproductive number can be approximated as $R_{t}=\frac{\gamma }{\epsilon }\frac{\left[H\right]}{{\left[H\right]}_{0}}$. The immune efficacy stands for the immune system’s strength to combat viral infection. The viral load will increase when *ϵ*<*γ*[*H*]/[*H*]_0_ (*R*_*t*_>1) and decrease when *ϵ*>*γ*[*H*]/[*H*]_0_. In the limit case when *ϵ* = 0, the viral load will exhibit unbounded increase (S1 Fig).

The immune efficacy is the numerical product of the killing and clearing effects by multiple innate and adaptive immune elements. As both immune arms actively participate in the killing of infected cells and clearance of virus particles, we denote innate immunity killing as $\epsilon_{k}^{i}\equiv f_{AntV}^{APC}k_{1}^{kill}[APC^{l}]+f_{AntV}^{APC}k_{2}^{kill}[APC^{u}]+f_{eff}^{NK}k_{3}^{kill}[NK]$, innate immunity clearance as $\epsilon_{c}^{i}\equiv f_{AntV}^{APC}k_{1}^{clear}[APC^{l}]+f_{AntV}^{APC}k_{2}^{clear}[APC^{u}]+k_{3}^{clear}[Neut]$, cellular immunity killing as $\epsilon_{k}^{a}\equiv k_{4}^{kill}[CTL]+k_{5}^{kill}[CD8+T_{M}]$ and humoral immunity clearance as $\epsilon_{c}^{a}\equiv k_{4}^{clear}A[Ab]$. The immune efficacy, by definition, is $\epsilon =(\epsilon_{k}^{i}+\epsilon_{k}^{a}+d_{If})(\epsilon_{c}^{i}+\epsilon_{c}^{a})$. Thus theoretically it can be dissected into innate immunity $\epsilon_{i}=(\epsilon_{k}^{i}+d_{If})\epsilon_{c}^{i}$, and adaptive immunity *ϵ*_*a*_ = *ϵ*−*ϵ*_*i*_ = *ϵ*_*aa*_+*ϵ*_*ai*_, where $\epsilon_{aa}=(\epsilon_{k}^{a}+d_{If})\epsilon_{c}^{a}$ is the pure cooperation between T cell and antibody, and $\epsilon_{ia}=\epsilon_{k}^{i}\epsilon_{c}^{a}+\epsilon_{c}^{i}\epsilon_{k}^{a}$ is the cooperation between adaptive immune elements with innate immunity (S8 Fig).

### Classification of immune response against SARS-CoV-2

Human immune response during infection varies by individual and is variable according to age, physical condition and gender [54,55]. This individual randomness, in addition to variation in initial inoculum and personal susceptibility, determines the progress and severity of infection.

To ensure our model generates reasonable results of immune response, we constrained the levels of immune cells and cytokines in our model within physiological range (S1 and S3 Tables) based on the immune profiles of peripheral blood [56,57], bronchoalveolar lavage fluid [58] and our clinical data (S24 Fig). We then used the Latin hypercube sampling method [59] to generate suitable kinetic parameter sets that satisfy the above constrained condition (Method 2). In our model, we set virulence of the wild-type SARS-CoV-2 strain as *γ* = 3.6 *day*^−2^ and initial viral load as 10^4^/mL.

To understand the heterogeneous outcome of infection, we simulated immune response over time from randomly sampled parameter sets, and classified the immune responses into four modes to reflect increasing severity of infection. Our simulations recapitulate clinically observed viral load dynamics and immune responses (S5 and S6 Figs). These modes are defined by the final viral load and IL-6 peak, to reflect the prolonged recovery of infection [60,61], and inflammatory status in patients. In particular, prolonged viral shedding [62,63] and elevated IL-6 level [64,65] have been found to correlate with more severe cases. Mode 1, 2 and 3 are characterized by recovery from infection with virus having been cleared by the 50^th^ day after infection. Mode 4 is characterized by persistent infection with [nCoV]>10^6^/mL at 50 days post-infection. Boundaries distinguishing the four modes based on IL-6 peaks are set as following: Mode 1: [IL-6]_max_<1000 pg/mL, Mode 2: 1000 pg/mL<[IL-6]_max_<2000 pg/mL, and Mode 3 and 4: [IL-6]_max_>2000 pg/mL. In our model, we ascribe Mode 1~3 with their increasing IL-6 level to patients experiencing more extensive infection and more severe symptoms. Meanwhile, Mode 4 patients are severe or critical care patients with chronic infection which most notably occurs in immunocompromised cases [66,67] and the elderly [60]. The choice of the IL-6 boundary values aims to reflect the increasing inflammation status, and change in the boundary values do not change the qualitative results we discuss below (S2 Fig).

**Fig 2:**
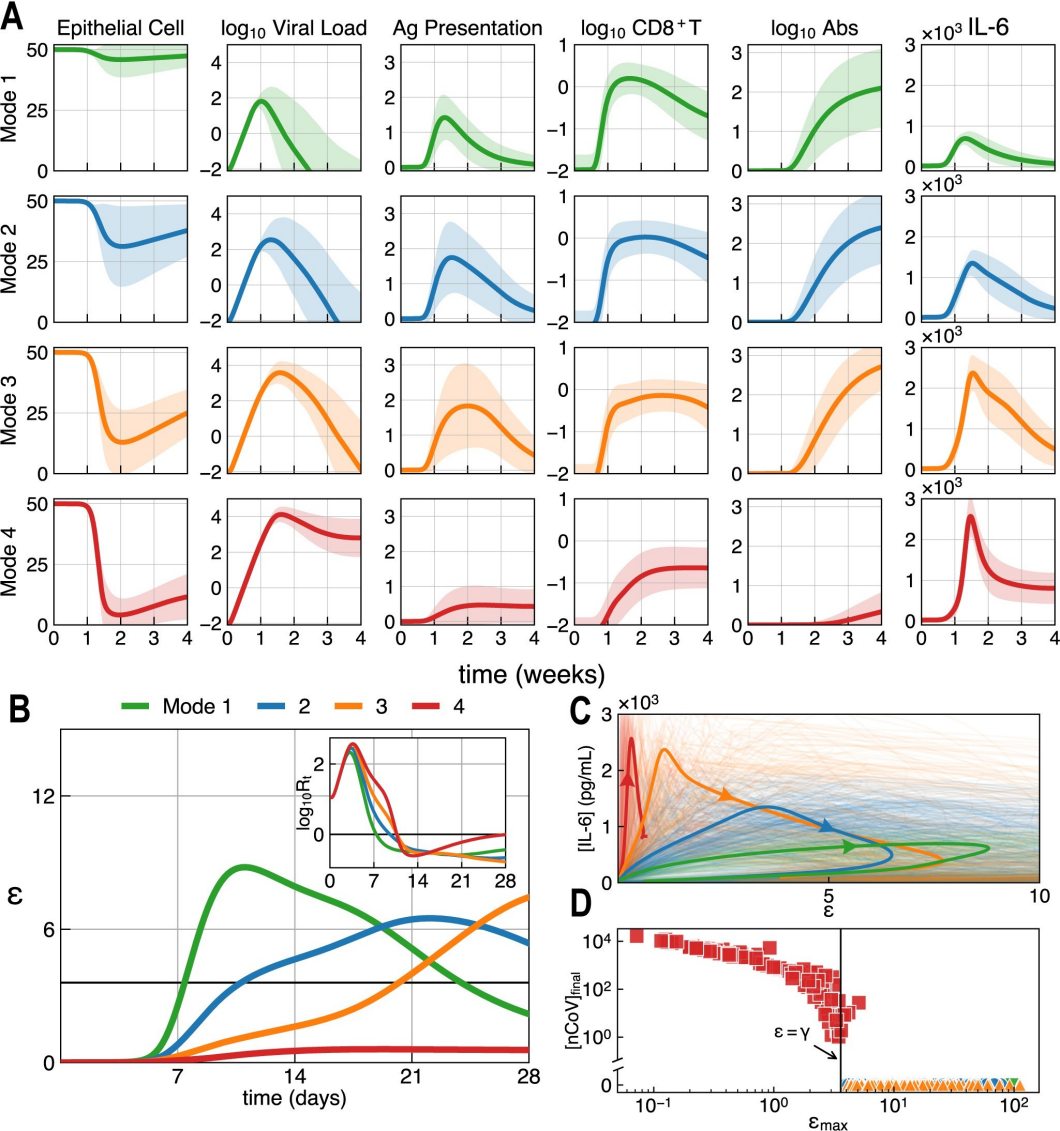
Dynamic trajectories of host immune response against SARS–CoV–2 infection. **(A)** Schematic illustration (mean±std) of four typical modes of immune response. The four immune response modes are defined by their maximum IL–6 level and viral dynamics, as defined in main text. The concentrations of viral load, CD8+ T cells, and Abs are illustrated as the geometric mean±std of samples, and other variables are plotted as the algebraic mean±std of samples. **(B)** Time course of immune efficacy *ε* of the four immune response modes, as shown in the geometric mean of all samples in the mode. Inset figure illustrates the time course of viral reproduction number *R*_*t*_, which is also calculated by the geometric mean of all samples in the mode. The solid line is *ε* = *γ* = 3.6 and *R*_*t*_ = 1. During the first two weeks after viral challenge, Mode 3 and 4 have lower *ε* compared to Mode 1 and 2, resulting in higher *R*_*t*_. Thus, extensive viral infection leads to greater tissue damage and resultant cytokine storm. *ε* value of Mode 1~3 rises in the following weeks, corresponding to full activation of the immune system. Meanwhile, poor response of adaptive immunity of Mode 4 patients leads to persistent infection. **(C)** Dynamic trajectories of SARS–CoV–2 infection are projected onto the 2D plane of *ε* and IL–6. Background: individual trajectories from sampling. Solid lines indicate trajectories of SARS–CoV–2 infection of Mode 1, 2, 3 and 4. Following the direction of the arrows, quicker immune response normally alleviates inflammatory status. **(D)** The relationship between final viral load [*nCoV*]_*final*_ and maximum of immune efficacy *ϵ*_*max*_ of all samples in Mode 1~4. *ϵ*_*max*_>*γ* serves as a necessary condition of viral clearance.

Sample-averaged kinetics in Fig 2A and S4 Fig reveal several characteristics emerging from each defined mode. For instance, the extent of tissue damage in Mode 1 and 2 is milder than that in Mode 3 and 4, and adaptive immunity in Mode 4, especially Ab dynamics, is significantly lower than that in other modes. Using this approach, our simulation results can predict the different degrees of SARS-CoV-2 pathogenicity. That is, upon viral infection in mode 1 and 2 patients, antigen-presenting cells are quickly activated and limit infection by clearing the virus and infected cells, recruiting circulating APCs, and presenting antigen to lymphocytes (S4 and S9 Figs). T cell response and Ab response are subsequently activated, and they function in the prescribed manner to clear virions and kill infected cells. Meanwhile, in Mode 3 and 4 patients, we see a less effective antigen presentation (S4B Fig), coupled with low proliferation of antigen-specific CD8+ T cells, as well as T cell exhaustion, resulting in a weak and delayed CTL response. Without sufficient and timely control by CTL cells, viral loads in Mode 3 and 4 patients continue to overwhelm the system. In the meantime, these severe cases exhibited weak immunosuppression (S7 Fig). Together, these immune signatures result in greater level of inflammation and tissue damage. Across Mode 1–4, the peak viral load increases, agreeing with the observed positive correlation between viral loads and infection severity [62,63,68,69] and mortality [70,71]. In Mode 3 patients, excessive damage-associated molecular patterns (DAMPs) subsequently elicit cytokine and chemokine secretions by APCs. These inflammatory signaling molecules further recruit and activate circulating innate cells, causing cytokine storm. Despite the elevated inflammation, antibody response does ramp up in Mode 3 patients and works together with innate and cellular immunity to clear the infection. However, in Mode 4 patients, unsuccessful recruitment of circulating APCs leads to inadequate innate and adaptive immune response, resulting in prolonged infection and potential risks for other complications and comorbidities.

We can further summarize this SARS-CoV-2 pathogenesis via the description of host immune efficacy, as illustrated in Fig 2B. During the early stage of infection in the first week, Mode 1 and 2 patients have faster and stronger innate immune protection of lung tissue against viral damage. In contrast, Mode 3 and 4 patients exhibit delayed and weaker immunity that brings about more extensive damage with higher viral load. Starting from the 2^nd^ week, cellular immunity comes in and cooperates with innate immunity to clear the infection (S8 Fig). The overall immunity of Mode 1 and 2 patients ramps up with peak immune efficacy *ϵ* averaged at *ϵ*>5>*γ* and viral reproduction number *R*_*t*_<1. Thus, the immune system handily clears the virus and kills infected cells. However, in the first two weeks after infection, Mode 3 and 4 patients experience more severe infection that not only results in extensive tissue damage, but also elicits over-activated inflammatory response by neutrophils and monocytes, leading to the onset of cytokine storm, as referenced above. During the 4^th^ week of infection, Mode 1~3 patients with higher *ϵ* recover from infection. Meanwhile, the immune responses of Mode 4 patients stay low with *R*_*t*_≈1, prolonging viral clearance, likely attributed to limited antibody production and T cell supply. In general, *ϵ*_*max*_>*γ* = 3.6 serves as a necessary condition for recovery from viral infection (Fig 2D). Dissecting the immune efficacy into innate immunity *ϵ*_*i*_, cooperation between innate and adaptive immune elements *ϵ*_*ia*_, and cooperation between T cell and antibody *ϵ*_*aa*_, we found the responsive speed and strength of innate immunity and cellular immunity are negatively correlated with disease severity (S8 Fig), agreeing with previous modelling works [37]. Meanwhile, in acute infection cases (Mode 1–3), adaptive immune response strength, especially antibody titer, is positively correlated with disease severity, due to prolonged antigen presentation (S4B Fig), corroborated by clinical observations [72,73]. Besides, by analyzing time series’ sensitivity, we found that the most sensitive parameters are related to the growth rate of host immune efficacy and virulence (S15 Fig and section 6.2.1 in S1 Text), e.g., activated CD8+ T cell division time (*t*_*CD*8_) and SARS-CoV-2 infection rate (*k*_*infect*_). In section 6.2.2 in S1 Text, we showed that the shifts of the fixed parameters do not change our main conclusions about immune dynamics and immune efficacy of Mode 1~4.

The dynamic interactions between viral infection and immune response revealed a general treatment principle for COVID-19 patients, which we tested *in silico*. In particular, in mode 3 and 4, early-stage weak immunity, as indicated by lower *ϵ*, leads to higher viral load and cytokine release. Excessive inflammation can be alleviated by decreasing virulence and augmenting innate immunity by antiviral agents like IFN-I (S13D Fig). In the late stage of mode 4, weak immunity prolongs the course of disease and opens the window for the onset of other systemic comorbidities. In this case, treatments with monoclonal antibodies could increase immune efficacy and accelerate the recovery process (see S13 and S14 Figs and Section 5 in S1 Text).

### Immune efficacy is a determinant of protection against SARS-CoV-2 infection and a predictor of vaccine efficacy

Vaccination plays a critical role in the global management of the COVID-19 pandemic to protect vaccinees from SARS-CoV-2 infection or from severe symptoms. Mechanistic insights into such immune protection will not only allow us to understand the roles of cellular and humoral immune memory [74,75], but also identify correlates of vaccine protection found in the immune system, i.e., memory T cells (cellular) and memory B cells or neutralizing antibodies (humoral). Identification of such correlates can link individual immune response to population protection, thus enabling the prediction of vaccine efficacy against all infection [76] in advance of large-scale phase 3 trials and assisting in future vaccine development [10,13].

**Fig 3:**
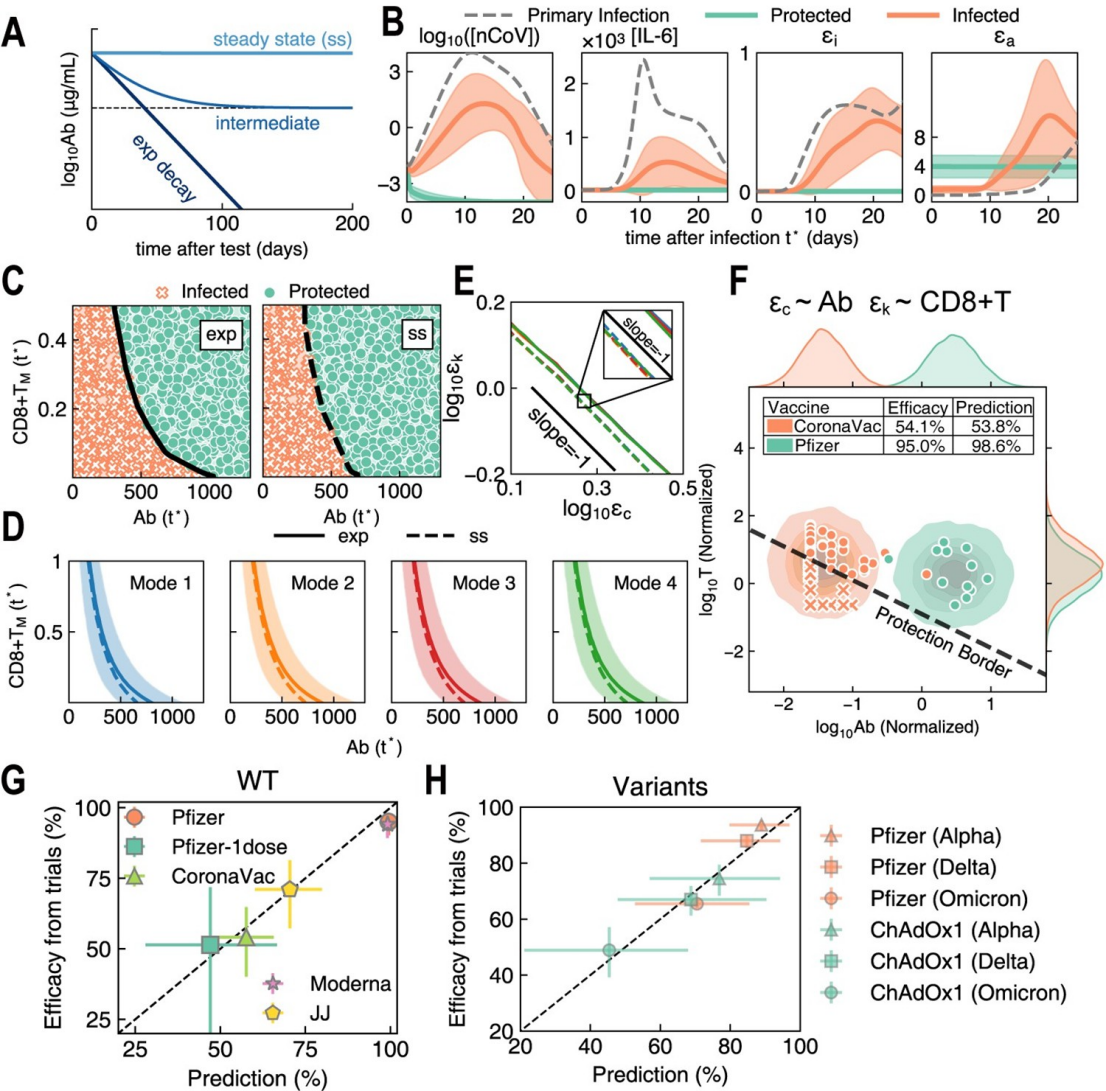
Immune memory protection and vaccine efficacy prediction. **(A)** Schematic time courses of antibody (Ab) titer after vaccination. For a given initial Ab level, Ab kinetics generally evolves as the ‘intermediate’ case where Ab titer is decaying to the steady–state with some antibody–secreting B_M_ cells. The ‘intermediate’ case lies between the two constraining cases, i.e., in the absence of B_M_, Ab decays exponentially and approaches to zero (‘exp’) and a certain level of B_M_ cells can still produce Ab and maintain Ab titer at steady–state (‘ss’). **(B)** Infection dynamics in primary infection without T_M_ and B_M_ (gray) and protected/infected cases after vaccination (teal & orange) for the ‘ss’ case. Teal trajectories represent individuals who can be defined as fully protected from infection based on their strong adaptive immunity and monotonic drop in viral load. Orange trajectories depict individuals who exhibit infection owing to insufficient immune efficacy. All samples are selected from one Mode 3 parameter set with uniformly randomized initial CD8+T_M_ and Ab levels. *ϵ*_*i*_ and *ϵ*_*a*_ stand for immune efficacy of innate and adaptive immunity, *ϵ* = *ϵ*_*i*_+*ϵ*_*a*_. See details in main text. **(C)** Sampling results of Fig 3B in the space of initial CD8+T_M_ and Ab (*t* = t*) for ‘exp’ cases (left) or ‘ss’ cases (right). The solid or dashed lines respectively represent full protection borders for ‘exp’ or ‘ss’ cases such that individuals with immune memory above the borders are protected from infection (teal lines in Fig 3B). **(D)** Full protection borders (mean±std) in Mode 1~4 samples are confirmatory of the general cooperation between cellular and humoral immunity. Solid line: ‘exp’ case; dashed line: ‘ss’ case. **(E)** By projecting all mean protection borders in Fig 3D onto the plane of *ϵ*_*c*_−*ϵ*_*k*_, the –1 slope confirms our theory. Inset: an enlarged view of the protection borders; only minor variance exists among different modes. **(F)** Immunogenicity distributions and estimation of protection rates of CoronaVac (teal) and BNT162b2 (Pfizer, orange) vaccines. Protection border separates protected individuals (circles) from susceptible individuals (crosses). A given vaccine induces a certain distribution of CD8+T_M_ and Ab immunogenicity in the recipient population, and protection rate corresponds to the fraction of recipients above the protection border. Each point in CoronaVac data stands for one participant. Since IFN–γ and Ab data of BNT162b2 cannot be matched, they were shuffled and paired for visual display. Inset table: predicted efficacies *vs*. reported efficacies from phase 3 trials. **(G)** Predictions of vaccine efficacy against wildtype (WT) SARS–CoV–2 based on immunogenicity data shown in Figs 3F and S23. **(H)** Predictions of vaccine efficacy against Alpha (triangles), Delta (diamonds) and Omicron (circles) variants. based on immunogenicity data shown in S23C Fig. (G–H) Vertical and horizontal error bars represent the 95% confidence intervals of reported vaccine efficacy and our prediction, respectively.

In clinical practice, antibody titer is the customary metric for immune protection [10]. Both plasma B cells (PB) and memory B cells (B_M_) produce antibodies, yet they have different lifespans. Antibody titer after vaccination should first decay exponentially owing to the rapid decrease of PB and then converge to a lower steady-state provided by B_M_. This scenario is situated between two sets of constraining kinetics. First, if PB is the sole source of antibody production and PB decays to zero after vaccination, then Ab will exponentially decay after reaching its peak level (denoted as ‘exp’). On the other hand, if B_M_ is the sole source of Ab, then Ab will be maintained at a high steady-state by B_M_ (denoted as ‘ss’) (Fig 3A). In addition to antibody protection, recent studies have demonstrated the important protective role of T cell immunity in vaccination [77–79]. Here, we discuss how memory T cells and antibodies work together in protecting the host from infection in the cases noted above, ‘exp’ and ‘ss’.

We started with an analysis of one case from Mode 3 (one parameter set in S5 Table) and then extended our results to all parameter sets in the four modes. In Fig 3B and 3C, we assumed that a vaccinated individual in Mode 3 is infected at time *t* = t*. We then investigated the dynamic processes of that individual’s immune response, while making the supposition that this individual has different levels of memory T cells and antibody titer corresponding to different levels of humoral and cellular immune memory induced by vaccination. In the simulation, we took initial viral inoculum of 10^4^/ml, fixed the initial CD4+T_M_ and sampled CD8+T_M_ and Ab level uniformly and independently. In the ‘exp’ case, we set B_M_ level as zero and initial antibody titer as *Ab*(t*); in the ‘ss’ case, B_M_ and *Ab*(t*) reach steady-state (Method 3). In Fig 3B, we plotted the infection dynamics in the ‘ss’ case. When an individual has a higher initial adaptive immune efficacy *ϵ*_*a*_(t*), he/she will be protected from infection, and the virus will be cleared directly. We defined fully protected or full protection as a monotonic drop in viral load (teal lines in Fig 3B). The host with insufficient adaptive immunity is plotted as orange lines in Fig 3B, indicating that the virus has successfully escaped host immune surveillance, that viral load is increasing, and that full-blown viral infection is taking place. However, inflammatory responses are alleviated by immune memory. The ‘exp’ case shows behavior similar to that shown in Fig 3B, indicating that a sufficient initial *ϵ*_*a*_(t*) can fully protect the individual from infection.

Based on the sampling results in Fig 3B, we found in Fig 3C a negatively sloped border separating protected (full protection) from infected individuals in phase plane of initial Ab and initial CD8+T_M_ (*Ab*(t*)−*CD*8+*T*_*M*_(t*) plane) in both ‘exp’ and ‘ss’ cases. In all parameter sets of Mode 1~4, we obtained trends similar to those in Fig 3D. The averaged border lines indicate the critical condition in which cellular and humoral immunity work to protect the host from further infection. The fluctuations of borders arise from the variation of killing rate *k*^*kill*^ and clearance rate *k*^*clear*^ in different parameter sets.

By analyzing reproduction number and immune efficacy, we found that the sufficient condition for full protection is *ϵ*_*k*_*ϵ*_*c*_|_*t* = t*_>*γ* or *ϵ*|_*t* = t*_>*γ* (Fig 3E, Method 4). As this multiplication rule has been theoretically discussed [80], here we adopt the *ϵ*>*γ* criterion to determine efficacy of the vaccine. When infected after vaccination, a recipient with immune efficacy *ϵ* that satisfies the *ϵ*>*γ* criterion will be protected. Further, if we have the distribution of immune efficacy across a cohort of vaccinees, we can predict the protection rate of the vaccine. To accomplish this, we made the following assumptions and simplifications of the *ϵ* = *ϵ*_*k*_*ϵ*_*c*_>*γ* criterion. We assumed *ϵ*_*c*_ to be proportional to Ab and *ϵ*_*k*_ to be proportional to CD8+T cell, denoted as *T*. The sufficient condition of full protection should be *k*_*v*_·*T*·*Ab*>*γ*, and it is plotted as the black dashed line in Fig 3F, where *k*_*v*_ represents cytotoxicity of T cells and antibody affinity. We obtained *Ab* and *T* from the immunogenicity data of neutralizing antibody titers and IFN-γ fold changes from vaccine trials (S6 Table). Therefore, based on these input data (*Ab* and *T*), we can predict vaccine efficacy by the fraction of fully protected individuals who satisfy the protection condition *k*_*v*_·*T*·*Ab*>*γ* (Method 5). When considering vaccine efficacy against mutation strain *S* (noted as the subscript), we assumed that *k*_*v*_ and *γ* are constants, making (*γ*/*k*_*v*_)_S_ the only parameter. As shown in Fig 3F, when (*γ*/*k*_*v*_)_*WT*_ = 0.13 for SARS-CoV-2 wild-type strain (WT), we have the best case prediction of vaccine efficacy for CoronaVac and Pfizer (BNT162b2) with root mean square error (RMSE) of 3.5%. Similarly, for SARS-CoV-2 Alpha variant (B.1.1.7), when (*γ*/*k*_*v*_)_*α*_ = 0.66, we can fit vaccine efficacy (RMSE = 3.8%) of BNT162b2 vaccine (Pfizer–BioNTech) and ChAdOx1 nCoV-19 vaccine (AstraZeneca). For the Delta (B.1.617.2) and Omicron variants, we have (*γ*/*k*_*v*_)_*δ*_ = 0.93 with RMSE = 4.3% and (*γ*/*k*_*v*_)_O_ = 2.11 with RMSE = 4.3%, respectively. Predictions for all variants are shown in Figs 3G, 3H and S23. Moreover, in S21 Fig, we discussed the sampling-based protection rates and their contributory factors, such as CD4+T memory levels and initial viral loads.

We did not discuss the dynamic processes of vaccination in this work, but simulated the production of antibodies and formation of immune memory following infection. Our results confirm the clinical findings [72,73] that antibody levels in severe patients are higher than patients with milder symptoms, which provides stronger protection against re-infection (S20 Fig). This could be attributed to prolonged APC activation and antigen-presentation process to B cells. However, mode 4 patients, who are normally immunocompromised and experience persistent infection, have significantly low antibody level to clear infection.

### Dynamics of SARS-CoV-2 variants: Competition between viral virulence *γ* and host immune efficacy *ε*

When people arm themselves with vaccines, the SARS-CoV-2 virus mutates simultaneously. Alpha, Delta and Omicron variants rapidly took over their predecessors and became dominant, and they increased their immune escape ability [17–20] and target cell affinity [81], which in turn broke through vaccine protection [82] and obtained greater transmissibility [14]. Delta variant caused increased viral load [83] and severe outcomes [84] in comparison to Alpha and wild-type, and Alpha infeciton showed higher viral load than that of wild-type [85]. We further incorporated the interactions between variants and the immune system into our computational framework and discussed how variants affect the individual- and population- level characteristics of primary infection and vaccine protection.

Considering within-host infection, the variants showed an array of features different from those of the WT strain, including (1) escape from current neutralizing antibodies via spike protein mutations, (2) increased viral affinity to angiotensin-converting enzyme 2 (ACE2), and (3) increased replication efficiency [86,87]. We depicted these characteristics in our model as (1) decreased antibody affinity, (2) increased infectivity *k*_infect_ and target cell abundance [*H*]_0_, and (3) larger burst size *N*_1_. These newly emerged characteristics contribute to increased virulence *γ*. However, it is difficult to calculate *γ* ≡ *N*_1_*d*_*If*_*k*_infect_·[*H*]_0_ directly. Thus, we integrated the quantitative evidence of antibody affinity and protection rates of vaccines against different variants, together with the protection condition *k*_*v*_·*T*·*Ab*>*γ*, to estimate the virulence *γ* of variants (Method 5). Virulence of the Alpha variant is estimated to be *γ*_*α*_ = 6.1 day^−2^ (5.5~12.2 day^−2^) and that of Delta is estimated to be *γ*_*δ*_ = 10.4 day^−2^ (9.5~10.7 day^−2^), while WT is *γ*_*WT*_ = 3.6 day^−2^ in our model. Detailed parameters can be found in S7 Table. In comparison to WT and other VOCs, the Omicron variant possesses faster proliferation in the bronchi, but reduced replication in the lung [88]. The immune response in bronchi may differ from that in lung. As our model mainly focuses on immune process in the lung area, we did not discuss the infection and immune dynamics of Omicron.

**Fig 4:**
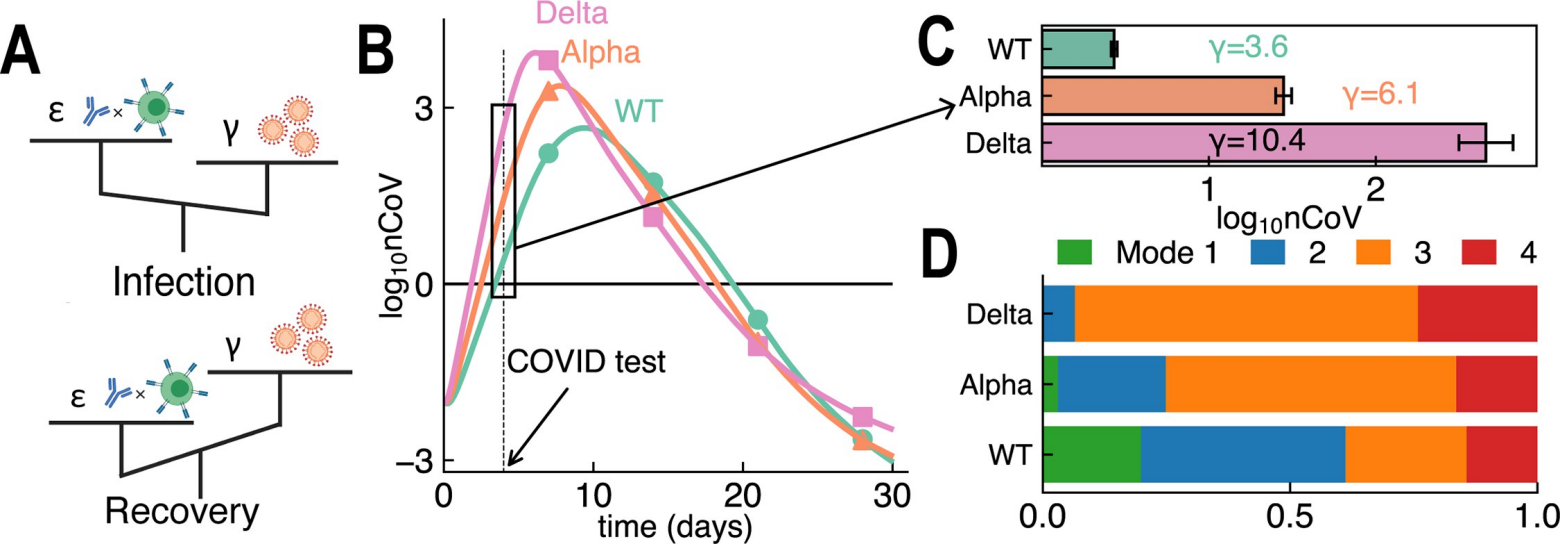
Viral virulence and host immunity dictate the viral dynamics of variants. **(A)** Host immune efficacy *ε* and viral virulence *γ* together determine *R*_*t*_ and thus the course of infection. When a patient’s immune response is evoked, *ε* increases to help clear the virus and kill infected cells, followed by recovery. **(B&C)** Greater *γ* leads to faster viral dynamics (B) and accounts for the ~100–fold difference in viral load (mean±std) of Delta variant upon COVID testing (C). **(D)** Percentages of four typical modes during the infection of wild type (WT), Alpha or Delta variants, where Alpha and Delta variants will induce more severe symptoms in the infected population. The sampling procedure on Alpha and Delta variants is same as that for WT in Fig 2.

The competition between viral virulence and the efficacy of host immune response determines the course and result of infection (Fig 4A). In Fig 4B and 4C, we simulated and illustrated different viral dynamics of WT, Alpha and Delta variants, considering the difference in virulence-related parameters. A greater *γ* yields more rapid viral dynamics; this explains the increased viral load in the Delta variant compared to WT during COVID testing.

Furthermore, in Fig 4D, we examined how Alpha and Delta variants affect within-host immune response and the percentage of four typical modes in the infected population. When infected with a highly virulent variant, the proportion of Mode 1 patients decreased (WT: 20%, Alpha: 3% and Delta: 0%, respectively), and the proportion of Mode 3 patients increased significantly (WT: 24%, Alpha: 59% and Delta: 69%). Our results are consistent with clinical report of increased severity and mortality rate in Alpha variant infections [89,90] and observed increased severity, hospitalization and emergency care risks in Delta infections [91–93]. However, other studies also suggested no difference in infection severity between Delta period and pre-Delta period [94] or even milder symptoms in Delta infections [95], which could be attributed to confounding factors, including vaccination and immediate treatments after infection. We note the fractions of Mode 1–4 are subject to the sampling method and classification boundary, thus their values cannot be directly compared with the actual severity distribution of COVID patients observed clinically. In S21 Fig, we also examined how increased virulence, decreased antibody affinity and higher initial viral inoculum affect immune protection and lower both full protection and severe prevention rates (see definition in Section 7 of S1 Text). In addition, with a few parameters modified (S8 Table), our model revealed infection dynamics of SARS and Influenza A Virus in S19 Fig.

### Clinical immune efficacy correlates with COVID-19 severity

Even as we demonstrated that immune efficacy can be a powerful framework in determining the strength of immune response, it is typically difficult to make the same determination, as directly and longitudinally measured, in clinical settings. Therefore, we herein propose a method to infer immune efficacy by using patients’ clinical hemogram, followed by evaluating the correlation between immune efficacy and disease severity.

We collected the longitudinal data of hemogram and cytokine profiles of 213 patients infected with WT SARS-CoV-2 strain in Feb 2020 from Wuhan Union Hospital in China. All patients were divided into mild/moderate, severe and critical groups based on their clinical symptoms, according to the *Novel* Coronavirus Pneumonia Diagnosis and Treatment Plan (Trial version 7) [96].

**Fig 5:**
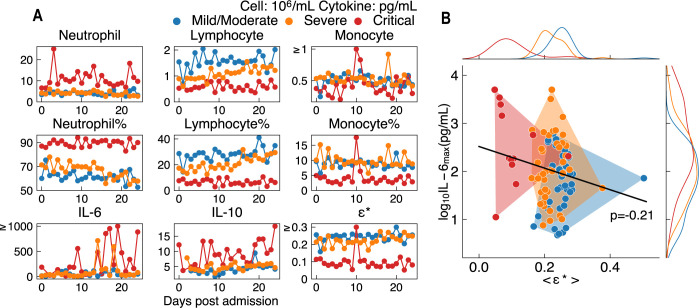
Hemogram and cytokine data of 95 patients with COVID–19. **(A)** We analyzed and illustrated the averaged (by each day) kinetics of peripheral blood immune cells and cytokine profile for all 95 patients (40 mild/moderate, 43 severe and 12 critical). Mild/moderate, severe and critical patients exhibit different degrees of inflammation (IL–6 peak and neutrophil count) with different values of clinical immune efficacy ϵ*, where ϵ* = (*Mono*%+*Neut*%)×(*Mono*%+*Lymph*%). Lower clinical efficacy ϵ* in critical patients represents weak immune response. **(B)** Distribution of patients’ maximum IL–6 level and averaged ϵ* over the first 25 days. Distinct distribution of <ϵ*> is observed. [*IL*−6]_max_ is found to be negatively correlated with <ϵ*> (Pearson Coefficient p = –0.21).

As generally acknowledged, mild/moderate, severe and critical patients show different levels of lymphopenia and IL-6 peak levels (Fig 5A). Immune efficacy *ϵ* dictates the multiplicative manner in which APC, NK, T cells work cooperatively with APC, Neut, and Ab. Ideally, to calculate *ϵ*, it would be necessary to measure the pulmonary level of these immune cells and antibody, as well as the killing and clearing rates of these effectors. However, such calculation is difficult given the limited clinical data available. We therefore attempted to find a feasible solution to estimate immune efficacy (S26 Fig), and we propose an empirical indicator for clinical immune efficacy as ϵ* = (*Mono*%+*Neut*%)×(*Mono*%+*Lymph*%), which is defined by the proportion (percentage) of neutrophils (*Neut*%), lymphocytes (*Lymph*%) and monocytes (*Mono*%). Based on patients’ IL-6 level and clinical immune efficacy, we discussed the classification of clinical patients in S27 Fig. Despite our coarse method, we still found ϵ* proved to be effective as an empirical reflection of immune efficacy. In particular, mild/moderate, severe and critical patients have average ϵ* of 0.25, 0.21, and 0.10, respectively, and negatively correlates with maximum IL-6 level (Fig 5B). Similarly, we have also observed the negative correlation between IL-6 level and averaged immune efficacy in our simulation results (S10 Fig), in which the immune efficacy could be used to classify the patients for its mapping with final viral load (Fig 2D). ϵ* proves to be a good biomarker since it incorporates the characteristics of lymphopenia and high neutrophil counts in severe and critical patients, but excludes the inflation in WBC counts owing to the large quantity of neutrophils (Section 8 in S1 Text). If Ab kinetics, as well as the classification of lymphocytes, are further provided, this empirically defined indicator could be further refined. Ideally, further feedback from a clinical perspective would help in defining a more effective indicator for immune efficacy.

### Inferring immune efficacy from viral shedding data

We exemplify another method for maximum estimate of immune efficacy based on a patient’s viral dynamics. We collected and analyzed viral shedding data by nasopharyngeal swab and sputum for a total of 171 individuals from three recent publications [34,97,98] and data points for a single patient≥3. We built a simplified SARS-CoV-2 infection model in Eqs 4~5 wherein we ignored the mucosal immunity term *ϵ*_*v*_.


$$\frac{d[nCoV]}{dt}=N_{1}d_{If}[If]-\epsilon_{c}[nCoV]$$ (4)

$$\frac{d[If]}{dt}=k_{\mathrm{infect}}[nCoV]{\left[H\right]}_{0}-\epsilon_{k}[If]$$ (5)

**Fig 6:**
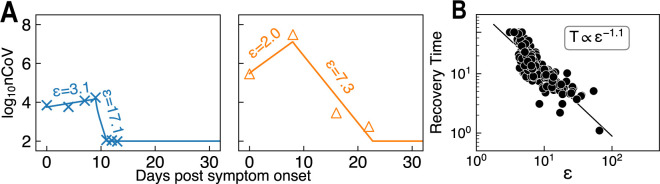
Fitting immune efficacy to viral shedding data. **(A)** Two examples to estimate immune efficacy in both rising and declining phase by fitting Eqs (4~5). **(B)** In the declining phase, the value of *ε* determines patients’ recovery time.

For simplification, in the rising phase of viral kinetics before viral load peak, we set *ϵ*_*c*_ = 1, and in the declining phase after viral load peak, we set *ϵ*_*k*_ = 3. Thus, by fitting *ϵ*_*c*_ or *ϵ*_*k*_ to the rising (24 individuals) or declining (171 individuals) data, we could calculate immune efficacy *ϵ* = *ϵ*_*c*_*ϵ*_*k*_ (see the two examples in Fig 6A and S28 Fig). Since we lacked a disease severity classification for most of the data, we alternatively used maximum viral load as the indicator for patients’ status [34,98]. Among the 24 patients within the rising phase, we found that their maximum viral loads were negatively correlated with immune efficacy (S29 Fig, Pearson p = -0.42). This confirms our results that weak immunity in the early stage may exacerbate patients’ conditions over the course of disease. Other factors, including patients’ susceptibility (therefore *γ*) and viral inoculum, could also affect maximum viral load. Meanwhile, declining stage immune efficacy determines the duration of viral shedding with a power law of -1.1 (Fig 6B). Thus, lower *ϵ* may result in slower recovery, which could potentially contribute to prolonged viral shedding, and subsequent severe lymphopenia, pulmonary damage, and bacterial co-infection. Therefore, patients with compromised immune response are more likely to fall into the critical category and require extra attention.

## Discussion

In this study, we constructed a mathematical model to describe innate and adaptive immune responses, as well as immune memory, upon infection with SARS-CoV-2 and its variants. We put forward a series of quantitative indicators to describe individual immune response, such as immune efficacy *ϵ*, the capability of immune system clearing the virus and killing infected cells (*ϵ*_*c*_ and *ϵ*_*k*_). We also defined virulence *γ* to depict the infectivity of different SARS-CoV-2 variants in the host. Our results showed that the contest between virus virulence *γ* and immune efficacy *ϵ* dictates infection and vaccine protection processes wherein lymphocytes and antibodies, together, contribute to immune efficacy in a multiplication manner.

To analyze the heterogeneity of an infected population, we classified the dynamics of immune responses into four modes to represent increasing severity of SARS-CoV-2 infection. In our work, we based our classification on the maximum IL-6 level and final viral load. IL-6, as an inflammatory cytokine has been found as one of the most prominent biomarkers for severe symptoms [64]. Final viral load is used to identify cases of prolonged viral shedding [60,66,67]. In other modelling works, different criteria are selected to define viral infection severity, including infected cell fraction [38], accumulated tissue damage [31,37] and PAMP and DAMP level [36]. How the difference in these indicators affect the final conclusion should be further explored. In our results, we showed that individuals with faster-responding immune efficacy will usually experience less severe symptoms, agreeing with another modelling study reporting mild symptom patients have higher innate immune response and faster CD8 T cell response [36,37].

Based on the mathematical formulation of immune efficacy, we proposed the numerical product of CD8+ T cell and antibody can be used to predict the protection rates of vaccines. Previous works have used antibody level or its dose response for vaccine efficacy [10,11], yet the cellular immunity has been overlooked [74,75]. Evidences suggest CD8+T cells render protection to vaccinated population when the NAb response is waning or escaped [99,100]. In macaques models, vaccine-induced CD8 immunity cooperates with antibodies to protect against SARS-CoV-2 [101] and SHIV [102]. These evidences and our theoretical results together stress the importance of cellular immunity in vaccine design.

Based on the vaccine efficacy results and our framework around γ-ε competition, we estimated the virulence for alpha and delta variants, and examined the consequence of increasing virulence in primary infection. However, this estimation is coarse, whose verification demands for measurements of virion burst size, infection rate, etc. To our knowledge, such measurements or estimates are usually difficult to obtain [103], where appropriate model could shed light on [4]. We then applied the multiplication formulation of immune efficacy *ϵ* in the clinical diagnosis and treatment processes (Figs 5 and 6, Section 8 in S1 Text), and revealed that lower immune efficacy is associated with more severe symptoms and longer recovery. When different immune-based biomarkers have been proposed and applied clinically, including WBC, lymphocyte, antibody level [104] and C reactive protein [105], we expect the immune efficacy can work as a quantitative indicator representing immune response strength of the host clinically. In future work, we hope to sharpen our theoretical framework in comparison to clinical findings by integrating the effect of cytokine expression and antibody level.

Due to the prolonged duration and rapid spread of the SARS-CoV-2 pandemic, a swarm of variants have arisen and are currently propagating simultaneously [106]. The diverse mixture of the rampaging variants further complicates the situation for our model to predict the epidemiological features of the newly infected. However, our model can still specifically estimate the virulence of single variant and simulate the host immune responses against its infection. Furthermore, incorporating more detailed information of the variant swarm, our framework could potentially be extended to model immune response and predict the vaccine efficacy against combinations of SARS-CoV-2 variants.

We acknowledge a few limitations in the present work. First, our model mainly focused on immune response of SARS-CoV-2 infection in lung and nearby draining lymph nodes; we did not consider systemic clinical symptoms like multiple organ failure, pathological damage of other organs, or preexisting conditions and comorbidities [107]. In our recent work [35], we also investigated bacterial infection in severe COVID-19 patients, together with the circulation of lymphocytes and cytokines among blood, lung, primary and secondary lymphoid organs. Second, in this work, we utilized IL-6 level and viral load to represent infection severity and classify immune responses. In the future, we will improve the classification criteria to depict severity more comprehensively. Finally, we need more clinical and animal data to verify our model, calibrate the kinetic parameters, and test our predictions.

In summary, our work provides a quantitative framework to investigate the dynamic mechanism of host immune response confronting SARS-CoV-2 virus infection. We hope to capture the essential dynamic properties of the host immune response. Thus, we anticipate that our approach can be adapted to other kinds of viral and bacterial infections and that it can be applied to describe and predict the cytokine storm on CAR-T immune treatment [53,108].

## Materials and methods

### 1. Ethics statement

From February 1 to February 29, 2020, 213 laboratory-confirmed COVID-19 admitted cases with authenticated outcome, either discharged or deceased, were collected at Union Hospital. The severity of disease (mild/moderate, severe and critical) was assessed according to the *Novel Coronavirus Pneumonia Diagnosis and Treatment* Plan (Trial version 7) [96]. Clinical information for all recruited patients was collected from the hospital electronic history system. This study was conducted in accordance with the Declaration of Helsinki and was approved by the Ethics Committee of Union Hospital, Tongji Medical College, and Huazhong University of Science and Technology (#2020/0004). Written informed consent was waived owing to the emergence of this high-risk infectious disease.

### 2. Sampling method

To understand population heterogeneity in immune response and clinical conditions during SARS-CoV-2 infection and any other infectious diseases, it is necessary to explore the parameter space of the viral-immune interaction network and identify plausible immune response patterns. To explore the parameter space for a system with 32 variables and 160 parameters, we reduce the dimensionality and size of sampling to increase efficiency. We fix the dissociation constants (Hill constants) for terms representing the system’s dynamics, including dying rate of immune cells, production rate and decay rate of cytokines and virulence-related parameters (infection rate, infected cell dying rate and burst size). Then we sample the kinetic rates of cellular interactions, including CD4+ and CD8+ T cell pool size, using the Latin Hypercube Sampling [59] method in the logarithmic space of ${log}_{10}(P)\in [{log}_{10}(P_{i})-b,{log}_{10}(P_{i})+b]$. The range for each sampled parameter and values for each fixed parameter can be found in S2 Table.

In the sampling process, the initial value (S3 Table) of each sample is fixed for ODE integration (Python scipy library [109], odeint function). The initial value for virus is set at 0.01×10^6^/mL; the initial value for infected cells is set at 0; initial values of naïve CD4+ and CD8+ T cells are the sampling parameters, *CD*4^+^*T*_*N*_ and *CD*8^+^*T*_*N*_; initial values of other variables are set at their steady-state solutions.

To further constrain the parameter space, we first estimated the physiological range of immune cells and cytokines in lung area (S1 Table). As we sampled through the parameter space in the section entitled ‘Classification of immune response against SARS-CoV-2’, we screened off the parameter sets, the ODE solutions of which were observed to lie outside the physiological range. The remaining parameter sets and their ODE solutions (dynamic trajectories) were selected for following classification.

Owing to the complexity of patients’ status as a whole, clinical conditions (asymptomatic, mild, moderate, severe and critical) are diagnosed based mainly on their symptoms. Here, we intend to focus on the dynamic processes of both viral infection and immune response. Therefore, we classified the dynamic trajectories into four typical modes (main text, S4 Table) to reflect patients’ inflammatory response. We assume Mode 1~4 patients will experience increasing inflammatory response and that Mode 4 patients are representative of critical patients and hence take longer to recover from COVID-19.

### 3. Vaccine protection simulation

For the initial conditions in infection after vaccination, we sampled CD8+T_M_ (0~5×10^5^/mL) and antibody titer Ab (0~1200μg/mL) uniformly and independently we fixed the initial CD4+ T_M_ as 2×10^4^/mL and antibody affinity as 1. In the ‘exp’ case, initial B_M_ equals 0. In the ‘ss’ case, $\frac{d[Ab]}{dt}(t^{*})=0$, and initial antibody titer reaches steady-state, as determined by memory B cells. Thus, initial B_M_ is calculated as

$$[B_{M}](t^{*})=\frac{c_{Ab}}{p_{2}^{Ab}(1+h_{IL-4}^{Ab}\frac{{\left[IL-4\right]}_{ss}}{K_{3}^{IL-4}+{\left[IL-4\right]}_{ss}})}[Ab](t^{*}),$$

where [*IL*−4]_*ss*_ is the steady-state of IL-4 concentration. The initial viral inoculum of 10^4^/ml and initial values of naïve CD4+ and CD8+ T cells are half of the sampling parameters, *CD*4^+^*T*_N_/2 and *CD*8^+^*T*_*N*_/2. Initial values of other variables are set at their steady-state solutions.

### 4. Condition for full protection by immune memory

We revisited and analyzed immune efficacy *ε* to understand the cooperative relationship between cellular and humoral memory. Because full protection is defined as a monotonic drop in viral load, this requires that the reproduction number remain *R*_*t*_<1. As healthy target cells [*H*]≤[*H*]_0_ and immune efficacy increase at the beginning of infection, we have $R_{t}=\frac{\gamma }{\epsilon_{k}(\epsilon_{c}+\epsilon_{v})}\frac{\left[H\right]}{{\left[H\right]}_{0}}<\frac{\gamma }{\epsilon_{k}\epsilon_{c}}\leq \frac{\gamma }{\epsilon_{k}\epsilon_{c}}{|}_{t=t*}<1$. Thus, after vaccination, the sufficient condition for full protection is *ϵ*_*k*_*ϵ*_*c*_|_*t* = t*_>*γ* or *ϵ*|_*t* = t*_>*γ*. Simulations verified the multiplication rule of cooperation between cellular (*ϵ*_*k*_) and humoral (*ϵ*_*c*_) immunity (necessary and sufficient condition) in Fig 3E.

### 5. Vaccine data analysis, efficacy prediction and estimation of variant virulence

We fitted the demographic distribution of cellular and humoral immune memory levels elicited by different vaccines to independent lognormal distribution (data of CoronaVac [110], ChAdOx1 nCoV-19 [17,111,112], and BNT162b2 [113,114]; details in S6 Table) and assumed these data to be same as the immunogenicity data in the trials for vaccine efficacy estimation. The immunogenicity data can then be interpreted as vaccine efficacy based on demographic information embedded in the lognormal distribution.

Neutralizing antibody titer was normalized by the mean convalescent plasma antibody level in the same study, further used in a log scale, and denoted as log_10_(Ab). SARS-CoV-2-specific T memory cell data were divided into two groups: IFN-γ secreting cells detected by the Elispot assay and SARS-CoV-2-specific CD8+T% detected by flow cytometry (S6 Table). For both data sources, SARS-CoV-2-specific T memory cell was defined as

$${log}_{10}(T)={log}_{10}{\left(\left[T\right]/[T\right]}_{0}-1),$$

where [*T*] is the IFN-γ response or CD8+T% in SARS-CoV-2-related peptides stimulated serum samples, and [*T*]_0_ is the baseline IFN-γ level or CD8+T% measured in the non-stimulated controls. For BNT162b2 IFN-γ response data, no baseline data were provided [113]. Both ChAdOx1 and BNT162b2 studies share the same IFN-γ detection method. Therefore, the [*T*]_0_ of ChAdOx1 was used to calculate log_10_(T) of BNT162b2. Inspired by the previous study [10], we fitted the vaccine data of log_10_(T) and log_10_(Ab) by the normal distribution $N(\mu_{T},\sigma_{T}^{2})$ and $N(\mu_{A},\sigma_{A}^{2})$, respectively. Here, we used the maximum likelihood estimation, and the likelihood function was

$$L(X|\mu ,\sigma ,l)=\prod_{x\in X}f{\left(x|\mu ,\sigma \right)}^{Sgn(x,l)}{\left[\int_{-\infty }^{x}f(s|\mu ,\sigma )ds\right]}^{1-Sgn(x,l)}.$$

For each vaccine, X is the set of log_10_(Ab) or log_10_(T_M_) data, and *f* is the probability function of a normal distribution with the mean *μ* and standard deviation *σ*. The function Sgn(x,l) = 1 when x > l (the limit of detection (LOD)), and Sgn(x,l) = 0 when x ≤ l. The negative log likelihood function was minimized to estimate the mean and the standard deviation. Thus, for one vaccine (*vax*), we fitted the parameters (*μ*_*T*_, *σ*_*T*_, *μ*_*Ab*_ and *σ*_*Ab*_) and described the populational humoral and cellular response with the joint normal distribution,

$$f_{vax}(x,y;\mu_{T},\sigma_{T},\mu_{Ab},\sigma_{Ab})=\frac{1}{\sqrt{2\pi }\sigma_{T}}e^{-\frac{{\left(y-\mu_{T}\right)}^{2}}{2\sigma_{T}^{2}}}\frac{1}{\sqrt{2\pi }\sigma_{Ab}}e^{-\frac{{\left(x-\mu_{Ab}\right)}^{2}}{2\sigma_{Ab}^{2}}}$$

where x is log10(Ab), and y is log10(T). Notably, the statistical parameters (*μ*_*T*_, *σ*_*T*_, *μ*_*Ab*_ and *σ*_*Ab*_) are different between vaccines.

In Method 4, we derived full protection condition *k*_*v*_·*T*·*Ab*>*γ* for individuals.

Then, for one specific virus strain (*V*), we can predict the vaccine efficacy (*E*) of vaccine (*vax*) against *V*,

$$E(vax,V)=\int_{-\infty }^{+\infty }\int_{\log_{10}{\left(\frac{\gamma }{k_{v}}\right)}_{V}-y}^{+\infty }f_{vax}(x,y)dxdy$$

We changed the value of *(γ/k*_*v*_*)*_*v*_, computed *E(vax*, *V)* for each vax, and calculated the root mean square error (RMSE) based on the real vaccine efficacy reported in the clinical trials. Then, we took the value of *(γ/k*_*v*_*)*_*v*_ with lowest RMSE as the best one for the strain *V*.

It is noteworthy that the reported efficacy of ChAdOx1 against WT [112] (62.1%) is even lower than that against Alpha [17] (74%) and Delta [17] (67%). Because of this confusion in data, we chose not to fit vaccine efficacy against WT to the data of ChAdOx1.

Variant virulence is calculated by the reported antibody affinities and the (*γ*/*k*_*v*_) we estimated. Fitting to vaccine efficacy data gives (*γ*/*k*_*v*_) of Alpha and Delta variants to be 5.08- and 7.15-fold that of WT.

$\frac{\gamma_{Alpha}/k_{v,Alpha}}{\gamma_{WT}/k_{v,WT}}=\frac{{\left(\gamma /k_{v}\right)}_{Alpha}}{{\left(\gamma /k_{v}\right)}_{WT}}=\frac{0.66}{0.13}=5.08$ and $\frac{\gamma_{\mathrm{Delta}}/k_{v,\mathrm{Delta}}}{\gamma_{WT}/k_{v,WT}}=\frac{{\left(\gamma /k_{v}\right)}_{Delta}}{{\left(\gamma /k_{v}\right)}_{WT}}=\frac{0.93}{0.13}=7.15$.

Meanwhile, the antibody affinity of different variants is assumed to be proportional to k_v_ in the model and is reported [115,116] to decrease by 1.5- to 3.3-fold for Alpha (*k*_*v*,*WT*_/*k*_*v*,*Alpha*_∈(1.5,3.3)) and 2.4- to 2.7-fold for Delta (*k*_*v*,*WT*_/*k*_*v*,*Delta*_∈(2.4,2.7)). Thus, with the virulence of WT as 3.6 in the model, the ranges of variant virulence are 5.5~12.2 for Alpha and 9.5~10.7 for Delta. For example, we take *k*_*v*,*WT*_/*k*_*v*,*Delta*_ = 2.4 and

$$\gamma_{Delta}=\frac{\gamma_{\mathrm{Delta}}/k_{v,\mathrm{Delta}}}{\gamma_{WT}/k_{v,WT}}\cdot \gamma_{WT}\cdot \frac{k_{v,Delta}}{k_{v,WT}}=7.15\times 3.6\times \frac{1}{2.4}\approx 10.725.$$

### 6. Clinical data analysis

From February 1 to February 29, 2020, 213 laboratory-confirmed COVID-19 admitted cases with authenticated outcome, either discharged or deceased, were collected at Union Hospital. The severity of disease (mild/moderate, severe and critical) was assessed according to the *Novel Coronavirus Pneumonia Diagnosis and Treatment* Plan (Trial version 7) [96]. As patients recovered and were discharged, the decreasing data volume increased the uncertainty by individual randomness. Also, considering the potential for bacterial infection in the late stage of COVID-19 infection, we finally chose to analyze the longitudinal data of patients from day 0 to day 25, with at least 2 time points (95 patients, 40 mild/moderate, 43 severe and 12 critical) in the main text.

## Supporting information

S1 Text Model details and additional analyses. (PDF)

S1 Fig Simulation of time course of viral dynamics and immune response under extreme cases where only one arm of immunity exists. (PDF)

S2 Fig Choice of IL-6 boundary values for classification does not change the immune response. (PDF)

S3 Fig T cell supply fluxes and classification of Mode 4. (PDF)

S4 Fig Sampling results. (PDF)

S5 Fig Comparison of viral load time courses between model simulation and clinical data. (PDF)

S6 Fig Comparison of viral load, humoral and cellular response between model simulation and COVID-19 patients’ clinical data. (PDF)

S7 Fig Severe cases (Mode 3 and 4) exhibited signatures of weaker immunosuppression, manifested by lower Treg activation level and suppressive cytokine IL-10 / TGF-β. (PDF)

S8 Fig The dynamics of innate immune efficacy *ϵ*_*i*_, adaptive immunity *ϵ*_*aa*_ and their cross term *ϵ*_*ia*_. (PDF)

S9 Fig Class-based principal component analysis (CPCA) on the parameter samples. (PDF)

S10 Fig Immune efficacy as a criterion to classify the modes. (PDF)

S11 Fig Immune dynamics of the 4 modes with different IFN-I levels. (PDF)

S12 Fig Simulations about the non-cytopathic effects of IFN-γ on viral infection and immune response. (PDF)

S13 Fig Treatment strategies for in silico patients. (PDF)

S14 Fig Q value distribution and sensitivity, and the *ε_i_-ε_a_* perspective of treatment strategies. (PDF)

S15 Fig Parameter analysis on the model. (PDF)

S16 Fig Robustness against fixed parameters. (PDF)

S17 Fig Robustness against random sampling. (PDF)

S18 Fig Virulence dictates maximum viral load during infection. (PDF)

S19 Fig Infection dynamics of SARS and Influenza A Virus (IAV). (PDF)

S20 Fig Immunogenicity data from model simulation of primary infection. (PDF)

S21 Fig Vaccine protection simulation. (PDF)

S22 Fig Full protection surface and severe prevention surface in initial Ab, B_M_ and CD8+T_M_ space. (PDF)

S23 Fig Immune memory distributions of different vaccines and predictions of efficacy against wildtype SARS-CoV-2 and variants. (PDF)

S24 Fig Physiological range of the cytokines in 95 clinical patients infected with WT SARS-CoV-2 virus. (PDF)

S25 Fig CPCA results on patients’ peripheral blood data. (PDF)

S26 Fig Different definition of clinical indicator for immune efficacy. (PDF)

S27 Fig A total of 95 clinical patients are classified by their IL-6 level and immune efficacy in peripheral blood. (PDF)

S28 Fig Fitting the viral dynamics data to the model (Eqs (4) ~ (5) in the main text) gives out a maximum estimate for *ε*. (PDF)

S29 Fig Statistics on the fitted immune efficacy. (PDF)

S1 Table Estimated cell density in lung area and draining lymph nodes. (PDF)

S2 Table Choice of parameter and sampling range. (PDF)

S3 Table Variable descriptions and choice of initial parameters. (PDF)

S4 Table Definition of Mode 1, 2, 3, and 4 patients in the main text. (PDF)

S5 Table The geometric mean parameter sets of Mode 1~4. (PDF)

S6 Table The sources of vaccine immunogenicity data and efficacies for different variants. (PDF)

S7 Table Choice of parameters of original strain (nCoV), Alpha and Delta variants viral infection and immune response. (PDF)

S8 Table Change of parameters for modelling SARS-CoV-1 infection and Influenza A Virus infection. SARS-CoV-1 induces stronger innate immunity and higher level of inflammation.
(PDF)

S9 Table The change on the model made by various drugs and parameters. (PDF)
