# Supplementary material for: Dynamical modelling of viral infection and cooperative immune protection in COVID-19 patients
Source: PLoS Comput Biol. 2023 Sep 1;19(9):e1011383. doi: 10.1371/journal.pcbi.1011383 (PMC10501599; doi:10.1371/journal.pcbi.1011383)
Supplement: S1 Text — (PDF) [file pcbi.1011383.s001.pdf]

Supplemental Information for

# Dynamics of SARS-CoV-2 and host immunity in infection and vaccine protection

**Authors:** Zhengqing Zhou[a,1], Dianjie Li[a, 1], Ziheng Zhao[b,1], Shuyu Shi[c,1], Jianghua Wu[d,1], Jianwei Li[a], Jingpeng Zhang[a], Ke Gui[b], Yu Zhang[b], Qi Ouyang[a], Heng Mei[d,*], Yu Hu[d,*], and Fangting Li[a,*]

[1]These authors contributed equally to this work.

[*]To whom correspondence should be addressed. Email: dr_huyu@126.com, hmei@hust.edu.cn, lft@pku.edu.cn

# Contents

1. Basic assumptions ........ 3
  1.1 Assumptions of the network ........ 3
  1.2 Basic assumptions of the ODE model ........ 4
  1.3 IFN-I in SARS-CoV-2 infection ........ 5
2. The Ordinary Differential Equation Model ........ 6
  2.1 T cell activation dynamics ........ 6
  2.2 Immune efficacy and derivation of $R_t$ ........ 6
  2.3 CD8+T supply affects the recovery process of mode 4 ........ 7
  2.4 Ordinary differential equations of the immune system against SARS-CoV-2 infection ........ 9
  2.5 Derivation of R0 based on next generation matrix ........ 12
  2.6 Simplified model for limit cases where only one arm of immunity is active ........ 13
  2.7 Non-cytopathic effect of IFN-γ ........ 14
3. Parameter Estimation ........ 15
4. Numerical methods ........ 17
  4.1 Sampling method ........ 17
  4.2 Class-based Principal Component Analysis ........ 18
5. *In silico* Treatment Strategies ........ 19
6. Sensitivity Analysis ........ 20
  6.1 Q definition and assessment of drug efficacy ........ 20
  6.2 Parameter sensitivity and robustness ........ 21
7. Immune Memory Protection and Vaccine Efficacy ........ 22

7.1 Vaccine protection rates in simulation ..... 22
8. Clinical indicators for immune efficacy ..... 22
SI References ..... 24

# 1. Basic assumptions

## 1.1 Assumptions of the network

1. We include IL-2, IL-4, IL-6, IL-10, IFN-γ, TNF-α in our model for they play different roles during infection. IL-2 has pleiotropic effects in T cell activation, as it both stimulates proliferation of conventional T cells and maintain Treg cells homeostasis[1,2]. IL-4 mediates Th2 differentiation and stimulates Ab production[3]. IL-6 is the major inflammatory cytokine in COVID patients[4]. IL-10 is a immunosuppressive cytokine that contains excessive inflammation[5]. TNF-α is a pleiotropic cytokine and we include it into our model for its pro-inflammatory effects[6]. IFN-γ is primarily secreted by CD4+ and CD8+ T cells and NK cells, and activates the pro-inflammatory, cytotoxic, and antigen-presentation response in macrophages[7,8]. While other cytokines also play important roles in the immune system, we do not include them in our network for overlapping functions and lack of reference in our clinical data. In particular, we do not include IFN-I for its significantly weakened role in SARS-CoV-2 infection (main text).
2. We take APC to be two states: antigen-loaded APC, $APC^l$, which executes antigen presentation, inflammation and effector functions, and APC not loaded up, $APC^u$, which mainly perform effector functions, and will be loaded upon antigen activation.
3. We consider the immunosuppressive function of Treg cells by two-fold: secretion of inhibitory cytokines like IL-10 and TGF-β, and a direct contact-dependent inhibitory effect. Other parts of Treg functions are omitted, including the role of CTLA-4 in inhibiting the antigen-presentation process and the role of CD25 extensively expressed on the surface of Treg cells that robs IL-2 from other lymphocytes.
4. We combine the functionally similar parts together, e.g. DC and MΦ are defined together as APC, IL-10 and TGF-β are also defined together.
5. We do not consider the effects of the hypothalamic-pituitary-adrenal (HPA) axis on the immune system, where secretion of cortisol inhibits body's immune response

and serves as an underlying factor for lymphopenia.

6. Multiple models regarding immune memory formation have been formulated. Here we adopt the linear development model for memory T cells and memory B cells where we assume low antigen level (low $APC^{l}$) promotes memory cell differentiation from effector cells.

### 1.2 Basic assumptions of the ODE model

1. We focus on the host immune response in lung and nearby draining lymph nodes (lung area).
2. With reference to the clinical data of serum cytokine level, we assume the cytokine level in lung area to be 10 times that of peripheral blood data. Change of this ratio does not affect the results of our model.
3. During Latin hypercube sampling, we assume virulence-related parameters (viral infectivity $k_{\mathrm{infect}}$, target cell abundance $[H]_0$, burst size $N_1$ and the dying rate of infected cells $d_{If}$) to be the same among the population, as to provide a reference for the level of $\varepsilon$ value and take initial viral load to be $10^4$/mL.
4. IL-6 is selected as the key indicator of inflammation [9,10].
5. The activation and differentiation processes of CD4+ and CD8+ T cells are highly TCR-dependent and the antigen-specific naïve T cells normally takes up $10^{-4}$ of the total T cell repertoire[11], thus we assume the SARS-CoV-2-specific naïve T cells change with the course of infection. On contrary, APCs, NKs and neutrophils are Ag-independent, and B lymphocytes undergo somatic hyper-mutation and affinity maturation during infection, we assume their pools to be sufficient and remain constant over time.
6. We assume the decay rate of cytokines to be constant, made up of the uptake process by immune cells and degradation.
7. We use Hill function with low power (n = 2) to describe the complex process of antigen presenting by APC. Similarly, immune cell activation/inhibition by cytokines and other immune cells are formulated as Hill function with n = 1. For

the sake of simplicity, we assume other cell-cell interactions are linear.

### 1.3 IFN-I in SARS-CoV-2 infection

One of IFN-I's function is to protect target cells from further infection. In the experiments by Sheahan et al.[12] and analysis by Jenner et al.[13], the half-effect dosage of IFN-β to inhibit viral production and infection capacity of MERS in Calu3 cells is 625 pg/mL. IFN-β level in groups of cohorts have been reported to range between 1 pg/mL and 15 pg/mL in COVID patients' serum[14,15]. If we assume the cytokine concentration in peripheral blood is 10-times lower than in lung area (consistent with our estimation of other cytokines), IFN-β is still far below the working concentration. Moreover, IFN-β and IFN-α2 protection of Calu3 cell viability against SARS-CoV-2 infection have both been estimated to have a half-effect dosage above $0.01\ \mu g/mL$, which translates into $\sim 10^4\ pg/mL$ [16]. The half-effect dosage of IFN-α2 to inhibit SARS-CoV-2 replication is $14532\ pg/mL$ in VeroE6 cells [17]. In contrast, clinical observations range from $1 - 100\ pg/mL$ [18-20], covering different extent of disease severity. Again, if we factor in the 10-fold difference between peripheral blood and lung area, the IFN-α2 titer is still below the working concentration. We therefore argue that even if there is a difference in IFN-I level across the spectrum of infection severity, it may not contribute significantly to the disease progress, for it is below the working concentration.

To back up our reasoning, we assess the effect of IFN-I production on COVID patients symptoms using a modified version of the model. We consider IFN-I effects in three-fold: limiting the generation of productive infected cells, promoting the antiviral and inflammatory functions of antigen-presenting cells, and promoting the functioning of NK cells (Section 2.4).

Here we consider the effect of IFN-α2, set $K_{IFN-I} = 14532\ pg/mL$, and test the effect it has to the immune dynamics across the four modes. We tested the IFN-I level in lung area as high (1000 pg/mL), medium (300 pg/mL) and low (0 pg/mL), as according to the physiological range of COVID patients. As shown in Figure S11, the difference in IFN-I level results in minimal changes in the immune dynamics, as it's

below the half-effect concentration. Therefore, we do not consider INF-I's effect in the model.

# 2. The Ordinary Differential Equation Model

## 2.1 T cell activation dynamics

Upon activation, CD4+ and CD8+ T cells are activated, undergoes limited generations of expansion, and differentiate into effector T cells. For CD4+ T cells, they are estimated to expand for 9 times, with 10 hours per generation. For CD8+ T cells, the numbers are 15~20 times and 6~8 hours per generation[21]. While a linear model[22] for exponential growth and contraction fits great to experimental data, the model tends to diverge during our sample parameterization. For the sake of simplicity, we estimate the burst size of activated CD4+ T cell and CD8+ T cells ($2^{11}$ and $2^{12}$), and assume they differentiate into activated CD4+ and CD8+ T cells with average rates of $2^{11}/(11\times10$ hrs) and $2^{12}/(12\times6$ hrs), respectively.

## 2.2 Immune efficacy and derivation of $R_t$

Model's reproductive number $R_t \equiv \frac{N_1 d_{If} k_{infect}\cdot[H]}{(\varepsilon_c+\varepsilon_v)\cdot\varepsilon_k}$ quantifies the strength of immune response as $\epsilon(t) \equiv \epsilon_c(t)\cdot\epsilon_k(t)$. When viral load is comparatively large $[nCoV]/K_m \to +\infty$, the mucosal term $\varepsilon_v(t) \equiv d_v \frac{1}{K_m+[nCoV]}$ goes to 0.

The immune efficacy is the numerical product of the killing and clearing effects by multiple innate and adaptive immune elements. As both immune arms actively participate in the killing of infected cells and clearance of virus particles, we denote innate immunity killing as $\epsilon_k^i \equiv f_{AntV}^{APC}k_1^{kill}[APC^l] + f_{AntV}^{APC}k_2^{kill}[APC^u] + f_{eff}^{NK}k_3^{kill}[NK]$, innate immunity clearance as $\epsilon_c^i \equiv f_{AntV}^{APC}k_1^{clear}[APC^l] + f_{AntV}^{APC}k_2^{clear}[APC^u] + k_3^{clear}[Neut]$, cellular immunity killing as $\epsilon_k^a \equiv k_4^{kill}[CTL] + k_5^{kill}[CD8 + T_M]$ and humoral immunity clearance as $\epsilon_c^a \equiv k_4^{clear}A\,[Ab]$. The immune

efficacy, by definition, is $\epsilon = (\epsilon_k^i + \epsilon_k^a + d_{If})(\epsilon_c^i + \epsilon_c^a)$. Thus theoretically it can be dissected into innate immunity $\epsilon_i = (\epsilon_k^i + d_{If})\epsilon_c^i$, and adaptive immunity $\epsilon_a = \epsilon - \epsilon_i = \epsilon_{aa} + \epsilon_{ai}$ , where $\epsilon_{aa} = (\epsilon_k^a + d_{If})\epsilon_c^a$ is the pure cooperation between T cell and antibody, and $\epsilon_{ai} = \epsilon_k^i\epsilon_c^a + \epsilon_c^i\epsilon_k^a$ is the cooperation between adaptive immune elements with innate immunity.

When assuming viral load at pseudo steady state, we can also derive $R_t$ but with more steps. Since virus dynamics usually have faster timescales[23] and viral load is usually taken as the fast variable[24], we assumed $\frac{d[nCoV]}{dt} = 0$ and have the relation that $[nCoV] = \frac{N_1 d_{If}}{\epsilon_c + \epsilon_v}[If]$. Substituting the relation into the equation of $[If]$, we have

$$\frac{d[If]}{dt} = k_{infect}[nCoV][H] - \epsilon_k[If] = \epsilon_k(R_t - 1)[If]$$

The infected cell reproductive number is $R_t = \frac{\gamma}{\epsilon_k(\epsilon_c + \epsilon_v)}\frac{[H]}{[H]_0}$, same as the viral reproduction number). According to previous work[25], next step is to substitute $[nCoV]$ in above equation and obtain viral load equation. However, in our model, since $\epsilon_c$ and $\epsilon_v$ are time-dependent, the left side $\frac{d[If]}{dt}$ is not equal to $\frac{d[nCoV]}{dt}$ and we could not directly obtain viral load equation. One possible way is assuming $\epsilon_c$ and $\epsilon_v$ at pseudo steady states. Then, we have

$$\frac{d[nCoV]}{dt} = \epsilon_k(R_t - 1)[nCoV]$$

where $R_t$ is now denoted as the viral reproduction number.

## 2.3 CD8+T supply affects the recovery process of mode 4

Since the effector T cells are supplied by the activated T cells that are only differentiated from the naïve and memory T cells in the model,

$$\frac{d[CD8^+T_A]}{dt} = A_{CD8}\left(1 + h_{IL-2}^{CD8}\frac{[IL-2]}{K_5^{IL-2} + [IL-2]}\right)\left(\frac{2^{g_3}}{t_{CD8}g_3}k_{naive}^{CD8}[CD8^+T_N] + \frac{2^{g_4}}{t_{CD8}g_4}k_{mem}^{CD8}[CD8^+T_M]\right)$$
$$-k_{CTL}[CD8^+T_A] - k_{CD8}^{mem}\frac{K_{mem}^2}{K_{mem}^2 + [APC^l]^2}[CD8^+T_A] - d_{Treg}^{CD8}[Treg^a][CD8^+T_A]$$

we define the CD8+ T supply flux,

$$J_{S,CD8} = \frac{2^{g_3}}{t_{CD8}g_3}k_{naive}^{CD8}[CD8^+T_N] + \frac{2^{g_4}}{t_{CD8}g_4}k_{mem}^{CD8}[CD8^+T_M]\ .$$

Among the four typical modes of SARS-CoV-2 infection, CD8+ T cell supply fluxes of mode 4 are significantly lower than the supply fluxes of other 3 modes (Figure S3A). It indicates that the insufficient supply is one of the key reasons for cytokine storm and persistent infection fo mode 4. 1) At early stage, low T cell supply results in slow and weak CTL response, and thus the host suffer higher extent infection and cytokine storm; 2) At late stage, the exhausted T supply contributes to the lower CTL level, and finally the insufficient immune efficacy to clear the virus.

To further understand the role of T cell exhaustion in mode 4, we categorize the mode 4 patient into mode 4.1 and mode 4.2 by the change of CD8+ T supply flux in Figure S3B,

$$\Delta J_{S,CD8} = J_{S,CD8}(t = t_T) - J_{S,CD8}(t = 0) \rightarrow \begin{cases} > 0, \text{mode 4.1} \\ \leq 0, \text{mode 4.2} \end{cases},$$

where the CD8+ T cell exhuastion is defined as $\Delta J_{S,CD8} \leq 0$, and $t_T$ is day 50 of infection simulation ($t_T = 50$ days in this section).

The level of antigen presentation ($APC^l$), indeed, determines the exhasution of CD8+ T cell in mode 4 (Figure S3C). For mode 4.2, although the higher antigen signal ($APC^l$) stimulates quick and strong T cell response at early stage, it drains the pool (naïve T cell) rapidly and suppresses the production (memory cell differentialtion) of T supply. After cellular response peak in week 2, the exhaustion of CD8+ T cell induces weak CTL response. Therefore, at late stage, the immune efficacy is inadequate to clear viruses, and mode 4.2 suffers from pesistent infection. However, although mode 4.1 shows sufficient T supply strengthening the immune efficacy to kill infected cells, the slow innate response (APC) leads to weakened humoral response, and thus casuses a

persistent infection.

### 2.4 Ordinary differential equations of the immune system against SARS-CoV-2 infection

Based on the main assumptions in section 1, we integrated the immune response network into a set of 32-variable ordinary differential equations as following. The equations in black are used for generating all the results except for Figure S11, S12, S13, and S14. The terms in red are related to the drug treatments and are only added in the equations when simulating treatment effect. For example: $\alpha$ stands for the effect of antiviral drugs in reducing viral production and $k_5^{clear}[Ab]_{ex}[nCoV]$ stands for the treatment with monoclonal antibodies.

**Viral infection module:**

$$\frac{d[nCoV]}{dt} = \alpha N_1 d_{If}[If] - \{f_{AntV}^{APC} k_1^{clear}[APC^l] + f_{AntV}^{APC} k_2^{clear}[APC^u] + k_3^{clear}[Neut] + k_4^{clear} A[Ab] + k_5^{clear}[Ab]_{ex}\}[nCoV] - d_v \frac{[nCoV]}{K_m + [nCoV]} \quad (1)$$

$$\frac{d[If]}{dt} = \left(\frac{K_{IFN-I}}{[IFN-I] + K_{IFN-I}}\right) k_{infect}[nCoV][H] - \{f_{AntV}^{APC} k_1^{kill}[APC^l] + f_{AntV}^{APC} k_2^{kill}[APC^u] + f_{eff}^{NK} k_3^{kill}[NK] + k_4^{kill}[CTL] + k_5^{kill}[CD8^+T_M]\}[If] - d_{If}[If] \quad (2)$$

$$\frac{d[H]}{dt} = r_H - \left(\frac{K_{IFN-I}}{[IFN-I] + K_{IFN-I}}\right) k_{infect}[nCoV][H] - d_H[H] \quad (3)$$

$$\epsilon = \epsilon_k(\epsilon_c + \epsilon_v)$$

$$\epsilon_c = d_v \frac{1}{K_m + [nCoV]}$$

$$\epsilon_c = f_{AntV}^{APC} k_1^{clear}[APC^l] + f_{AntV}^{APC} k_2^{clear}[APC^u] + k_3^{clear}[Neut] + k_4^{clear} A[Ab]$$

$$\epsilon_k = f_{AntV}^{APC} k_1^{kill}[APC^l] + f_{AntV}^{APC} k_2^{kill}[APC^u] + f_{eff}^{NK} k_3^{kill}[NK] + k_4^{kill}[CTL] + k_5^{kill}[CD8^+T_M] + d_{If}$$

$$\frac{d[D]}{dt} = \{f_{AntV}^{APC} k_1^{kill}[APC^l] + f_{AntV}^{APC} k_2^{kill}[APC^u] + f_{eff}^{NK} k_3^{kill}[NK] + k_4^{kill}[CTL] + k_5^{kill}[CD8^+T_M]\}[If] + d_{If}[If] - d_D[D] \quad (4)$$

$$\frac{dA}{dt} = m[B_{GC}] \frac{[Tfh]}{K_1^{Tfh} + [Tfh]}(1 - A) \quad (5)$$

$$f_{inf}^{APC} = 1 + h_{IFN-\gamma}^{APC} \frac{[IFN-\gamma]}{K_1^{IFN-\gamma} + [IFN-\gamma]} + h_{TNF-\alpha}^{APC} \frac{[TNF-\alpha]}{K_1^{TNF-\alpha} + [TNF-\alpha]} + h_D^{APC} \frac{[D]}{K_1^D + [D]} + h_{IFN-I}^{APC} \frac{[IFN-I]}{[IFN-I] + K_{IFN-I}}$$

$$f_{AntV}^{APC} = 1 + h_{IFN-\gamma}^{APC}\frac{[IFN-\gamma]}{K_1^{IFN-\gamma} + [IFN-\gamma]} + h_{IFN-I}^{APC}\frac{[IFN-I]}{[IFN-I] + K_{IFN-I}}$$

$$f_{eff}^{NK} = 1 + h_{IL-2}^{NK}\frac{[IL-2]}{K_1^{IL-2} + [IL-2]} + h_{IFN-I}^{NK}\frac{[IFN-I]}{[IFN-I] + K_{IFN-I}}$$

**White Blood Cell dynamics:**

$$\frac{d[APC^l]}{dt} = \left(k_{nCoV}^{APC}[nCoV] + k_{If}^{APC}\frac{[If]}{K_1^{If} + [If]}\right)[APC^u] - d_{Treg}^{APC}[Treg^a][APC^l] - d_{APC^l}[APC^l] \ (6)$$

$$\frac{d[APC^u]}{dt} = r_{APC} + k_{rcr}^{APC}\frac{f_{inf}^{APC}[APC^l]}{K_1^{APC}+f_{inf}^{APC}[APC^l]}[APC_0] - (k_{nCoV}^{APC}[nCoV] + k_{If}^{APC}\frac{[If]}{K_1^{If}+[If]})[APC^u] - d_{APC^u}[APC^u] \ (7)$$

$$\frac{d[NK]}{dt} = \left(k_{If}^{NK}\frac{[If]}{K_2^{If} + [If]} + k_{APC}^{NK}\frac{f_{inf}^{APC}[APC^l]}{K_2^{APC} + f_{inf}^{APC}[APC^l]}\right)[NK_0] - d_{Treg}^{NK}[Treg^a][NK] - d_{NK}[NK] \ (8)$$

$$\frac{d[Neut]}{dt} = k_{If}^{Neut}\frac{[If]}{K_3^{If} + [If]}[Neut_0] + k_D^{Neut}\frac{[D]}{K_2^D + [D]}[Neut_0] + k_{Th17}^{Neut}\frac{[Th17]}{K_1^{Th17} + [Th17]}[Neut_0]$$

$$- d_{Neut}[Neut] \ (9)$$

$$\frac{d[CD4^+T_N]}{dt} = -k_{naive}^{CD4}A_{CD4}[CD4^+T_N] \ (10)$$

$$\frac{d[CD4^+T_A]}{dt} = A_{CD4}\left(1 + h_{IL-2}^{CD4}\frac{[IL-2]}{K_2^{IL-2} + [IL-2]}\right)\left(\frac{2^{g_1}}{t_{CD4}g_1}k_{naive}^{CD4}[CD4^+T_N] + \frac{2^{g_2}}{t_{CD4}g_2}k_{mem}^{CD4}[CD4^+T_M]\right)$$

$$- \left(k_{Th1} + k_{Th2} + k_{Th17} + k_{Tfh} + k_{iTreg}\right)[CD4^+T_A] - k_{CD4}^{mem}\frac{K_{mem}^2}{K_{mem}^2 + [APC^l]^2}[CD4^+T_A]$$

$$- d_{Treg}^{CD4}[Treg^a][CD4^+T_A] - d_{GC}^{lymph}[CD4^+T_A] \ (11)$$

$$\frac{d[Th1]}{dt} = k_{Th1}[CD4^+T_A] - d_{Treg}^{CD4}[Treg^a][Th1] - d_{Th}[Th1] - d_{GC}^{lymph}[Th1] \ (12)$$

$$\frac{d[Th2]}{dt} = k_{Th2}[CD4^+T_A] - d_{Treg}^{CD4}[Treg^a][Th2] - d_{Th}[Th2] - d_{GC}^{lymph}[GC] \ (13)$$

$$\frac{d[Th17]}{dt} = k_{Th17}[CD4^+T_A] - d_{Treg}^{CD4}[Treg^a][Th17] - d_{Th}[Th17] - d_{GC}^{lymph}[Th17] \ (14)$$

$$\frac{d[Tfh]}{dt} = k_{Tfh}[CD4^+T_A] - d_{Treg}^{CD4}[Treg^a][Tfh] - d_{Th}[Tfh] - d_{GC}^{lymph}[Tfh] \ (15)$$

$$\frac{d[Treg^a]}{dt} = k_{iTreg}[CD4^+T_A] + k_{APC}^{nTreg}A_{CD4}\frac{[IL\text{-}2]}{K_4^{IL-2} + [IL\text{-}2]}[Treg^r] - d_{Treg^a}[Treg^a] \ (16)$$

$$\frac{d[Treg^r]}{dt} = r_{Treg^r} - k_{APC}^{nTreg}A_{CD4}\frac{[IL\text{-}2]}{K_4^{IL-2} + [IL\text{-}2]}[Treg^r] - d_{Treg^r}[Treg^r] \ (17)$$

$$\frac{d[CD4^+T_M]}{dt} = k_{CD4}^{mem}\frac{K_{mem}^2}{K_{mem}^2 + [APC^l]^2}[CD4^+T_A] - k_{mem}^{CD4}A_{CD4}[CD4^+T_M] - d_{CD4Tm}[CD4^+T_M] \ (18)$$

$$\frac{d[CD8^+T_N]}{dt} = -k_{naive}^{CD8}A_{CD8}[CD8^+T_N] \ (19)$$

$$\frac{d[CD8^+T_A]}{dt} = A_{CD8}\left(1 + h_{IL-2}^{CD8}\frac{[IL-2]}{K_5^{IL-2} + [IL-2]}\right)\left(\frac{2^{g_3}}{t_{CD8}g_3}k_{naive}^{CD8}[CD8^+T_N] + \frac{2^{g_4}}{t_{CD8}g_4}k_{mem}^{CD8}[CD8^+T_M]\right)$$

$$- k_{CTL}[CD8^+T_A] - k_{CD8}^{mem}\frac{K_{mem}^2}{K_{mem}^2 + [APC^l]^2}[CD8^+T_A] - d_{Treg}^{CD8}[Treg^a][CD8^+T_A]$$

$$- d_{GC}^{lymph}[CD8^+T_A] \ (20)$$

$$\frac{d[CTL]}{dt} = k_{CTL}[CD8^+T_A] - \frac{k_4^{kill}}{N_{ex}}[CTL][If] - d_{Treg}^{CD8}[Treg^a][CTL] - d_{CTL}[CTL] - d_{GC}^{lymph}[CTL] \quad (21)$$

$$\frac{d[CD8^+T_M]}{dt} = k_{CD8}^{mem}\frac{K_{mem}^2}{K_{mem}^2+[APC^l]^2}[CD8^+T_A] - k_{mem}^{CD8}A_{CD8}[CD8^+T_M] - d_{CD8Tm}[CD8^+T_M] \quad (22)$$

$$\frac{d[B_{GC}]}{dt} = A_B\left(k_{naive}^{GC}[B_0] + k_{mem}^{GC}[B_M] + r_{gc}\frac{[Tfh]}{K_1^{Tfh}+[Tfh]}[B_{GC}]\right)\left(1-\frac{[B_{GC}]}{K_{GC}}\right) - k_{PB}A_B[B_{GC}] - k_{Bm}[B_{GC}]$$

$$- d_{gc}[B_{GC}] - d_{GC}^{lymph}[B_{GC}] \quad (23)$$

$$\frac{d[PB]}{dt} = k_{PB}A_B[B_{GC}] - d_{PB}[PB] - d_{GC}^{lymph}[PB] \quad (24)$$

$$\frac{d[B_M]}{dt} = k_{Bm}[B_{GC}] - k_{mem}^{\mathrm{GC}}A_B[B_M] - d_{Bm}[B_M] \quad (25)$$

$$[APC_0] = Constant,\ [NK_0] = Constant,\ [Neut_0] = Constant,\ [B_0] = Constant$$

$$A_{CD4} = f_{AntV}^{APC}\frac{[APC^l]^2}{K_{ACD4}^2+[APC^l]^2},\ A_{CD8} = f_{AntV}^{APC}\frac{[APC^l]^2}{K_{ACD8}^2+[APC^l]^2},\ A_B = f_{AntV}^{APC}\frac{[APC^l]^2}{K_{AB}^2+[APC^l]^2}$$

$$k_{Th1} = k_{CD4}^{Th1}\left(1 + h_{IFN-\gamma}^{Th1}\frac{[IFN\text{-}\gamma]}{K_2^{IFN-\gamma}+[IFN\text{-}\gamma]}\right)\frac{K_1^{IL-4}}{K_1^{IL-4}+[IL-4]}\frac{K_1^{IL-10}}{K_1^{IL-10}+[IL\text{-}10/TGF\text{-}\beta]}$$

$$k_{Th2} = k_{CD4}^{Th2}\left(1 + h_{IL-4}^{Th2}\frac{[IL\text{-}4]}{K_2^{IL-4}+[IL\text{-}4]}\right)\frac{K_3^{IFN-\gamma}}{K_3^{IFN-\gamma}+[IFN\text{-}\gamma]}\frac{K_1^{IL-10}}{K_1^{IL-10}+[IL\text{-}10/TGF\text{-}\beta]}$$

$$k_{Th17} = k_{CD4}^{Th17}\left(1 + h_{IL-6+TGF-\beta}^{Th17}\frac{[IL\text{-}6]}{K_1^{IL-6}+[IL\text{-}6]}\frac{[IL\text{-}10/TGF\text{-}\beta]}{K_2^{IL-10}+[IL\text{-}10/TGF\text{-}\beta]} + h_{Neut}^{Th17}\frac{[Neut]}{K_1^{Neut}+[Neut]}\right)\frac{K_1^{IL-10}}{K_1^{IL-10}+[IL\text{-}10/TGF\text{-}\beta]}$$

$$k_{Tfh} = k_{CD4}^{Tfh}\frac{K_1^{IL-10}}{K_1^{IL-10}+[IL\text{-}10/TGF\text{-}\beta]}\left(1 + h_B^{Tfh}\frac{[B_{GC}]}{K_1^{GC}+[B_{GC}]}\right)$$

$$k_{iTreg} = k_{CD4}^{iTreg}\left(1 + h_{IL-10}^{iTreg}\frac{[IL\text{-}10/TGF\text{-}\beta]}{K_3^{IL-10}+[IL\text{-}10/TGF\text{-}\beta]}\right)\frac{[IL\text{-}2]}{K_3^{IL-2}+[IL\text{-}2]}\frac{K_2^{IL-6}}{K_2^{IL-6}+[IL\text{-}6]}$$

$$k_{CTL} = k_{Ta}^{CTL}\left(1 + h_{Th1}^{CTL}\frac{[Th1]}{K_1^{Th1}+[Th1]} + h_{IL-2}^{CTL}\frac{[IL\text{-}2]}{K_6^{IL-2}+[IL\text{-}2]} + h_{IL-6}^{CTL}\frac{[IL\text{-}6]}{K_3^{IL-6}+[IL\text{-}6]}\right)\frac{K_4^{IL-10}}{K_4^{IL-10}+[IL\text{-}10/TGF\text{-}\beta]}$$

**Cytokine and antibody dynamics:**

$$\frac{d[IL\text{-}2]}{dt} = p_1^{IL-2}[CD4^+T_A] + p_2^{IL-2}[CD8^+T_A] + p_3^{IL-2}[Th1] + p_4^{IL-2}[CTL] - c_{IL-2}[IL\text{-}2] \quad (26)$$

$$\frac{d[IL\text{-}4]}{dt} = p_0^{IL-4} + p_1^{IL-4}[Th2] - c_{IL-4}[IL\text{-}4] \quad (27)$$

$$\frac{d[IL\text{-}6]}{dt} = p_0^{IL-6} + p_1^{IL-6}[If] + p_2^{IL-6}\beta_{GC}f_{inf}^{APC}[APC^l] + p_3^{IL-6}[Neut] - c_{IL-6}[IL\text{-}6] \quad (28)$$

$$\frac{d[IL\text{-}10/TGF\text{-}\beta]}{dt} = p_0^{IL-10} + p_1^{IL-10}[Treg^a] + p_2^{IL-10}[Treg^r] - c_{IL-10}[IL\text{-}10/TGF\text{-}\beta] \quad (29)$$

$$\frac{d[TNF\text{-}\alpha]}{dt} = p_0^{TNF-\alpha} + p_1^{TNF-\alpha}[If] + p_2^{TNF-\alpha}\beta_{GC}f_{inf}^{APC}[APC^l] + p_3^{TNF-\alpha}f_{eff}^{NK}[NK] - c_{TNF-\alpha}[TNF\text{-}\alpha] \quad (30)$$

$$\frac{d[IFN\text{-}\gamma]}{dt} = p_0^{IFN-\gamma} + p_1^{IFN-\gamma}f_{eff}^{NK}[NK] + p_2^{IFN-\gamma}[Th1] + p_3^{IFN-\gamma}[CTL] - c_{IFN-\gamma}[IFN\text{-}\gamma] \quad (31)$$

$$\frac{d[Ab]}{dt} = \left(1 + h_{IL-4}^{Ab}\frac{[IL-4]}{K_3^{IL-4}+[IL-4]}\right)\left(p_1^{Ab}[PB] + p_2^{Ab}[B_M]\right) - c_{Ab}[Ab] \quad (32)$$

## 2.5 Derivation of R0 based on next generation matrix

It has been shown that $R_0$ can be derived using next generation matrix[26]. We refer to the readers this note for an brief introduction[27]. Here we elaborate the definition of $R_0$, as well as $\varepsilon$, does not depend on the latent phase of the viral infection.

Consider a system including n infectious variables $x_1, \ldots, x_n$, following a set of ODEs $\frac{dx_i}{dt} = \mathcal{F}(x) - \mathcal{V}(x)$, where $\mathcal{F}(x)$ denotes the introduction of new infections to this group of variables, while $\mathcal{V}(x)$ denotes the transitions between different infectious states. According to the next generation matrix, the basic reproduction number $R_0$ is the dominant eigenvalue of the matrix $G = FV^{-1}$ where $F = \left[\frac{\partial \mathcal{F}_i}{\partial x_j}\right]$ and $V = [\frac{\partial \mathcal{V}_i}{\partial x_j}]$.

If we consider a model without latent infected cells, as our model used in main text and detailed in section 2.4, we have:

$$\frac{d[If]}{dt} = k_{infect}[nCoV][H] - \varepsilon_k[If]$$

$$\frac{d[nCoV]}{dt} = N_1 d_{If}[If] - \varepsilon_c[nCoV]$$

Without loss of generality, we denote $\varepsilon_x = \varepsilon_v + \varepsilon_c$, where $\varepsilon_v$ and $\varepsilon_c$ are defined in the main text. $F = \begin{bmatrix} 0 & k_{infect}[H] \\ 0 & 0 \end{bmatrix}$ and $V = \begin{bmatrix} \epsilon_k & 0 \\ -N_1 d_{If} & \epsilon_c \end{bmatrix}$.

Therefore $G = FV^{-1} = \begin{bmatrix} 0 & k_{infect}[H] \\ 0 & 0 \end{bmatrix} \begin{bmatrix} 1/\varepsilon_k & 0 \\ N_1 d_{If}/\varepsilon & 1/\varepsilon_c \end{bmatrix} = \begin{bmatrix} \gamma/\varepsilon & k_{infect}[H]/\varepsilon_c \\ 0 & 0 \end{bmatrix}$,

which gives out dominant eigenvalue of $R_0 = \frac{k_{infect} N_1 d_{If}[H]}{\varepsilon} = \frac{\gamma}{\varepsilon}$, with $\varepsilon = \varepsilon_k \varepsilon_c$.

Similarly, if we consider a model with one latent stage of infection, denoted as L, we have:

$$\frac{d[L]}{dt} = k_{infect}[nCoV][H] - (d_L + \varepsilon_L)[L]$$

$$\frac{d[If]}{dt} = d_L[L] - \varepsilon_k[If]$$

$$\frac{d[nCoV]}{dt} = N_1 d_{If}[If] - \varepsilon_c[nCoV]$$

Then $F = \begin{bmatrix} 0 & 0 & k_{infect}[H] \\ 0 & 0 & 0 \\ 0 & 0 & 0 \end{bmatrix}$ and $V = \begin{bmatrix} d_L + \varepsilon_L & 0 & 0 \\ -d_L & \varepsilon_k & 0 \\ 0 & -N_1 d_{If} & \varepsilon_c \end{bmatrix}$.

$$G=\begin{bmatrix}0 & 0 & k_{infect}[H]\\ 0 & 0 & 0\\ 0 & 0 & 0\end{bmatrix}\begin{bmatrix}\frac{1}{d_L+\varepsilon_L} & 0 & 0\\ \frac{d_L}{(d_L+\varepsilon_L)\varepsilon_k} & \frac{1}{\varepsilon_k} & 0\\ \frac{N_1 d_{If} d_L}{\varepsilon_c\varepsilon_k(d_L+\varepsilon_L)} & \frac{N_1 d_{If}}{\varepsilon_k\varepsilon_c} & \frac{1}{\varepsilon_c}\end{bmatrix}$$

$$=\begin{bmatrix}\frac{\gamma d_L}{\varepsilon_c\varepsilon_k(d_L+\varepsilon_L)} & \frac{\gamma}{\varepsilon_k\varepsilon_c} & \frac{k_{infect}[H]}{\varepsilon_c}\\ 0 & 0 & 0\\ 0 & 0 & 0\end{bmatrix}.$$

Thus, $R_0=\frac{\gamma d_L}{\varepsilon_x\varepsilon_k(d_L+\varepsilon_L)}$. During the latent period, as long as the latent infected cells are killed comparatively slower than their transformation into productively infected cells, $d_L \gg \varepsilon_L$, a proper approximation yields $R_0=\frac{\gamma}{\varepsilon_c\varepsilon_k}$.

### 2.6 Simplified model for limit cases where only one arm of immunity is active

When $\epsilon=0$, theoretically the viral load will exhibit unbounded growth. To discuss the scenarios when only one arm of immunity is active, we use a simplified model that sets the rates of innate immunity clearing the virus ($\epsilon_c^i$) and killing the infection cells ($\epsilon_k^i$), humoral immunity clearing the virus ($\epsilon_c^a$), and cellular immunity killing the infected cells ($\epsilon_k^a$) to be constant. Other processes and parameters are the same as in the model we used in the main text. In this simple model, the immune efficacy is $\epsilon=\left(\epsilon_k^i+\epsilon_k^a+d_{If}\right)\left(\epsilon_c^i+\epsilon_c^a\right)$.

$$\frac{d[nCoV]}{dt}=N_1 d_{If}[If]-(\epsilon_c^i+\epsilon_c^a)[nCoV]$$

$$\frac{d[If]}{dt}=k_{infect}[nCoV][H]-\left(\epsilon_k^i+\epsilon_k^a+d_{If}\right)[If]$$

$$\frac{d[H]}{dt}=r_H-k_{infect}[nCoV][H]-d_H[H]$$

Using the model described above, we can simulate the following limit scenarios. 1) If there is only cellular immunity, where we set $\epsilon_c^i=0, \epsilon_c^a=0, \epsilon_k^i=0, \epsilon_k^a=2$, we show the results in the upper panels of Figure S1. Even though cellular immunity is

killing infected cells, the viral load will keep increasing due to $\epsilon = 0$. 2) In another limit case where only humoral immunity exists, we set $\epsilon_c^i = 0, \epsilon_c^a = 2, \epsilon_k^i = 0, \epsilon_k^a = 0$. In this case, the viral load reaches a plateau in the end (middle panel of Figure S1). As $d_{If} \neq 0$, the immune efficacy $\epsilon = \epsilon_k \epsilon_c = \left(\epsilon_k^i + \epsilon_k^a + d_{If}\right)\left(\epsilon_c^i + \epsilon_c^a\right) = d_{If}\epsilon_c^a > 0$, thus the viral load is limited in the end. However, we note these two scenarios are unlikely to happen since both cellular and humoral immunity depends on innate immunity's activation through antigen presentation. 3) If only innate immunity exists, where we set $\epsilon_c^i = 1, \epsilon_c^a = 0, \epsilon_k^i = 1, \epsilon_k^a = 0$, the immune efficacy is also non-zero: $\epsilon = \left(\epsilon_k^i + d_{If}\right)\epsilon_c^i > 0$. (Lower panels of Figure S1)

## 2.7 Non-cytopathic effect of IFN-γ

The non-cytopathic effects exist extensively among virus infection diseases, which limit the viral production efficiently, especially when chronic infection occurs [28]. To further confirm whether non-cytopathic effects will affect the key conclusions of our model, the direct role of IFN-γ on the viral infection process (transition of heathy cells to infected cell) was considered. We assumed that the infection rate $k_{infect}^{eff}$ depends on IFN-γ levels (Figure S12A),

$$k_{infect}^{eff} = k_{infect}\left(1 - k_{IFN-\gamma}\frac{[IFN-\gamma]}{[IFN-\gamma] + K_{IFN-\gamma}}\right),$$

where $k_{infect}$ is the maximum infection rate, $k_{IFN-\gamma}$ represents the extent of the non-cytopathic effect, and $K_{IFN-\gamma}$ is the Hill constant. Then, the equations of healthy cells (H) and infected cells (If) are written as,

$$\frac{d[If]}{dt} = k_{infect}^{eff}[nCoV][H] - d_{If}[If]$$
$$- \left\{f_{AntV}^{APC}k_1^{kill}[APC^l] + f_{AntV}^{APC}k_2^{kill}[APC^u] + f_{eff}^{NK}k_3^{kill}[NK]\right.$$
$$\left. + k_4^{kill}[CTL] + k_5^{kill}[CD8^+T_M]\right\}[If]$$

$$\frac{d[H]}{dt} = r_H - k_{infect}^{eff}[nCoV][H] - d_H[H]$$

In Figure S12B, to investigate the non-cytopathic effect of IFN-γ on viral infection, we increased $k_{IFN\text{-}\gamma}$ and simulate different immune response with a Mode 3 parameter set. Simulations with medium level of non-cytopathic effect ($k_{IFN\text{-}\gamma} = 0.1$) show slightly lower peaks of viral load and IL-6 than the simulation without such effect ($k_{IFN\text{-}\gamma} = 0$). Only when the non-cytopathic effect is dramatically strong ($k_{IFN\text{-}\gamma} = 0.5$), i.e., inhibiting half of the healthy-infected cell transition, IL-6 exhibits slight reduction in its peak level. Moreover, after reclassifying sampling results into four Modes (Figure S12C), we found that the number of Mode 1 increases while the numbers of other Modes decrease with elevated $k_{IFN\text{-}\gamma}$. Accordingly, these results reveal the non-cytopathic effect on reducing inflammation and severity and impairing the infection.

To examine whether the non-cytopathic effect of IFN-γ would change our main result, we computed the averaged trajectories of immune efficacy ε and IL-6 for each Mode with different $k_{IFN\text{-}\gamma}$ (Figure S12D). In simulations of all four Modes, both trajectories of ε and IL-6 exhibit little change regardless of the value of $k_{IFN\text{-}\gamma}$. This confirms the main result that faster and higher immune efficacy leads to lower infection severity. The minor drops of peak ε with increased $k_{IFN\text{-}\gamma}$, are probably caused by reduced $k_{infect}^{eff}$, weaker virulence $\gamma = N_1 d_{If} k_{infect}^{eff}[H]_0$, and lower requirement for ε to control the infection.

## 3. Parameter Estimation

According to Liao et al[29], the 20mL bronchoalveolar lavage fluid (BALF) of healthy people has a total density of 1.17 to 2.1 $\times 10^4$ cells/mL, with a median proportion of 86.1% alveolar macrophage. This indicates the total alveolar macrophage count washed out is $2.8\times10^5$. In the meantime, according to Crapo et al[30], the normal alveolar macrophage count estimated by morphometric analysis is $23\pm7\times10^6$. Due to the incomplete washout as well as the infiltration of the macrophages in the pulmonary

tissue, only a fraction (1.2%) of cells could be obtained via BALF. During the same BALF procedure, we assume this fraction to be constant over different types of cells (macrophages, dendritic cells, natural killer cells, T cells and B cells). Based on this assumption, we can estimate the physiological range of different immune cells in lung tissue in health control as well as in COVID patients accordingly (Table S1). For instance, T cells take up 6.3% to 33.9% of BALF cells ($1.25\times10^5$ cells/mL to $2.25\times10^6$ cells/mL)[31] in severe and critical patients. Therefore, the T cell count in BALF would be:

$$N_{BALF}^{T} = 2.25 \times 10^6\ cells/mL \times\ 20\ mL\ \times 33.9\% = 15.3 \times 10^6\ cells$$

And the estimated T cell count in pulmonary tissue would be

$$N_{Lung}^{T} = N_{BALF}^{T}\ /\ 1.2\% = 1.275 \times 10^9\ cells$$

Given the total pulmonary tissue volume has been estimated[32] to be 843±110mL, the estimated T cell density in lung tissue would therefore be

$$[T] = 1.275 \times 10^9\ cells\ /\ 843mL = 1.51 \times 10^6\ cells/mL.$$

Moreover, T cells and B cells also reside in draining lymph nodes near lung area, where they are activated and actively proliferate. We therefore set the upper physiological limit for these cells to be higher than the estimation in lung area, of $8 \times 10^6\ cells/mL$ for T cell and $5 \times 10^6\ cells/mL$ for B cell.

We further assume the cytokine level in lung area to be 10-fold of peripheral blood, and thus estimated the physiological range of the cytokines based on clinical data (Figure S24).

Given the apoptosis or death rates of the immune cells and decay rates of the cytokines and antibody, and by further confining the variables within physiological range, activation/recruitment rates of the immune cells and secretion of cytokines are estimated (the parameters denoted by $k$, $h$, and $p$). These estimations are automatically executed during the sampling method where the parameter sets that produces out-of-physiology-range dynamics are screened off.

# 4. Numerical methods

## 4.1 Sampling method

To understand the population heterogeneity in immune response and clinical conditions during SARS-CoV-2 infection and any other infectious diseases, it is necessary to explore the parameter space of the viral-immune interaction network and identify the plausible patterns immune response. For a system with 32 variables and 160 parameters, it is impossible to exhaust the parameter space. Alternatively, we reduce the dimensionality and size of sampling to increase efficiency. We fix the dissociation constants (Hill constants) of the dynamical terms, apoptosis rates of immune cells, production rates and decay rates of cytokines and virulence-related parameters (infection rate, infected cell dying rate and burst size). Then we sample the kinetic rates of cellular interactions, antigen-presentation-associated Hill constants, CD4+ and CD8+ T cell pool size using Latin Hypercube Sampling[33] method in the logarithmic space of $\log_{10}(P) \in [\log_{10}(P_i) - b, \log_{10}(P_i) + b]$. Range for each sampled parameter and values for each fixed parameter can be found in Table S2.

In the sampling process, the initial value (Table S3) of each sample is fixed for ODE integration (python scipy library [34], odeint function). The initial value for virus is set at $0.01\times10^6$/mL; the initial value for infected cells is set at 0; the initial values of naïve CD4+ and CD8+ T cells are the sampled parameters, $CD4^+T_N$ and $CD8^+T_N$; initial values of other variables are set at their steady state solutions.

Due to the complexity of patient's status as a whole, clinical conditions (mild, moderate, severe, critical and asymptomatic) are diagnosed based mainly on patients' symptoms. While our model could not accurately reflect patients' conditions, but we intend to illustrate the relation between immune response and viral infection. We turn to the definition of mode 1 - 4, as to qualitatively reflect patients' inflammatory response and recovery time. Mode 1 - 4 are defined based on their viral dynamics and maximum IL-6 level, as in Table S4. We assume Mode 1 - 4 patients should experience increasingly extensive inflammation, and Mode 4 patients resemble

immunocompromised patients, therefore taking longer time to recover from COVID-19 (Figure S4A). Despite they are qualitatively similar, we do not intend to make a one-to-one correspondence between the four modes and asymptomatic, mild/moderate, severe, and critical patients, but to reflect the difference in patient's inflammatory response and recovery time. Our sample results converge when the samples within physiological range are greater than 1500 (Figure S4C, in our simulation, we use a sample size of ~3000).

**4.2 Class-based Principal Component Analysis**

Principal Component Analysis (PCA) serves as a dimensional reduction method normally used for identification of principal components of a group of unlabeled data and for reduction of data's dimensionality for further analysis[35]. In Principal Component Analysis (PCA), after standardization, the variance of a set of *n*-dimensional data, including *m* data points, projected onto the direction **u** is written as:

$$\frac{1}{m}\sum_{i=1}^{m}\left(\boldsymbol{x^{i}}^{T}\boldsymbol{u}\right)^{2} = \boldsymbol{u}^{T}\left(\frac{1}{m}\sum_{i=1}^{m}\boldsymbol{x^{i}}^{T}x^{i}\right)\boldsymbol{u}$$

with the ($n\times n$) matrix in the brackets standing for the covariance matrix of the dataset. The variance takes its maximum when **u** is the eigenvector of the covariance matrix.

Here, to identify the key characteristics in labeled data (e.g., key parameters in the samples of Mode 1~4 patients, and key biomarkers in mild/moderate, severe and critical COVID patients), we propose the Class-Based Principal Component Analysis (CPCA) as a similar linear dimensionality reduction tool targeting at labeled data. Suppose we have a *n*-dimensional data set which has already been categorized into *p* classes with $m_j$ points in class *j*. To best represent the data in *n'*-dimensional space, we will need different classes to be separated from each other and the data points in one class to be as much close to each other as possible. We can easily define the divergence of a group of points by the covariance matrix shown above. Thus, we define the matrix **M** to be:

$$M = \frac{1}{p}\sum_{j=1}^{p}\boldsymbol{x}_{j}^{COM^{T}}\boldsymbol{x}_{j}^{COM} - \sum_{j=1}^{p}\frac{w_{j}}{m_{j}}\sum_{i=1}^{m_{j}}\boldsymbol{x}_{j}^{i^{T}}\boldsymbol{x}_{j}^{i}$$

in which $\mathbf{x}_j^{COM}$ and $w_j$ stand for the center of mass and the total weight of class $j$. We require $\mathbf{u}^T\mathbf{M}\mathbf{u}$ to be maximum to separate different classes while keeping each class compact. Therefore, when reducing the dimensionality to $n'$, we can simply take the first $n'$ eigenvectors with the biggest eigenvalues to be the principal dimensions. We can further look for the best performance we want by adjusting $w_j$.

We performed CPCA on the sampled parameters (Figure S9), to infer the key factors related with the four modes, and clinical data (Figure S25), to find potential biomarkers for patients' diagnosis.

## 5. *In silico* Treatment Strategies

Reducing the duration of viral shedding and inhibiting excessive inflammation to avoid exacerbation are the main goals of COVID-19 treatment. To this end, we propose the treatment strategies should come in three-fold, by augmenting patient's immune efficacy $\epsilon$, reducing virulence $\gamma$ and directly inhibiting inflammatory cytokine secretion. We consider several mostly discussed agents for COVID-19: (1) Antiviral drugs (AntV)[36], for their role in inhibiting viral infection or production, thus reducing virulence; (2) IFN-I[37], for their role in inhibiting viral infection and increasing innate immune response; (3) Monoclonal antibody (Ab)[38], for their effect in binding and neutralizing virion particles; (4) Glucocorticoids (GC)[39,40], inhibiting excessive cytokine production.

Based on the dynamic trajectories of *in silico* patients, we put forward corresponding treatments for different modes and plot their dynamic trajectories as in Figure S10. For the clarity of discussion, we divided the course of disease into early stage (0~7 days p.i., p.i. = post infection), middle stage (7~14 days p.i.) and late stage (14+ days p.i.). Antiviral agents help reduce $\gamma$ without side effects, and are therefore recommended for Mode 2, 3 and 4 patients. For Mode 4 patients, the early use of IFN-I promotes innate immune efficacy and helps contain initial tissue damage, while the use of GC during middle stage can alleviate cytokine storm. For Mode 4 patients, aside from the cytokine storm during the middle stage, persistent infection during the late stage should be dealt

with combination of antiviral agents and monoclonal antibodies, to increase immune efficacy and reduce virulence, thus clear the virus.

To further quantify the efficacy of treatments, we turn to a model-based scoring function $Q$ as an indicator for patients' status. The $Q$ value is defined by patient's respiratory capacity (minimum healthy lung epithelial cells $[H]_{\min}$), inflammation level (maximum IL-6 level $[IL-6]_{\max}$), and whether persistent infection happens (final state viral load $[nCoV]_{final}$). It is formulated as

$Q \equiv \left(1+q_1\frac{[H]_{min}}{[H]_c+[H]_{min}}\right)\left(1+q_2\frac{[IL-6]_c}{[IL-6]_c+[IL-6]_{max}}\right)\left(1+q_3\frac{[nCoV]_c}{[nCoV]_c+[nCoV]_{final}}\right)$, where we set $q_1 = 1$, $q_2 = 2$, $q_3 = 1$, $[H]_c = 30 \times 10^6/mL$, $[IL-6]_c = 2000pg/mL$ and $[nCoV]_c = 1 \times 10^6/mL$. As shown in Figure S14A, $Q$ values for Mode 1, 2, 3 and 4 patients are centered at $Q$ = 7.8, 6.6, 4.6 and 1.9, respectively.

The efficacy for different treatments defined as the relative change in $Q$ value, $\Delta Q/Q$. During the procedure, outliers identified by Interquartile Range (IQR) method are excluded. The improvements in Mode 2, 3 and 4 patients for their combinatory treatments are $\Delta Q/Q$ =0.08, 0.1 and 0.4, respectively. Following the same procedure, we give predictions about the efficacy of AntV, IFN-I, Ab and GC. Despite complex situations in clinic that is beyond our model, our predictions align with several clinical trials and case reports, highlighting the significance of early usage of AntV[41], IFN-I[42], and Ab[43,44], for their role in limiting virus invasion and tissue damage, middle-stage usage of GC[45] for limiting the cytokine storm and middle- and late-stage Ab[46] for increasing the immune efficacy to clear the virus.

# 6. Sensitivity Analysis

## 6.1 Q definition and assessment of drug efficacy

$Q$ function-related parameters, e.g., the $q$ values and Hill constants in the definition of $Q$ and treatment-related parameters, e.g., the effect of antiviral agents $\alpha$ in reducing the production of virions from infected cells, the effect of glucocorticoids in reducing

inflammatory cytokine secretion $\beta$, are drawn from uniform distribution with the range of [50%, 150%] and [80%, 120%] of their original value, respectively. Mean and standard deviation of $\Delta Q/Q$ are shown in Figure S14B.

## 6.2 Parameter sensitivity and robustness

### 6.2.1 Time series' sensitivity

To confirm the reliability of our model and examine the key parameters, we performed parameter sensitivity analysis as following: by changing one single parameter by two-fold, we examine the relative change of the sample-averaged trajectory of the system. We define the distance between two trajectories to be

$$d = \sum_{i=1}^{32} \int \left\| \frac{r_i^{ori}(t) - r_i^{ptb}(t)}{max(r_i^{ori})} \right\|_2 dt$$

as the integration of the Euclidean distance between the two normalized trajectories (divided by the maximum of the original trajectory) along time. The change of the Mode 1~4 patients' trajectories are shown in Figure S15. In the figure, the ticks correspond to the indices of parameters in Table S2.

### 6.2.2 Sampling robustness against fixed parameters

We further assess the robustness of our sampling method against the choice of fixed parameters and their values. First, for each fixed parameter, we change it by two-fold (×2 or ×1/2) and repeat the sampling procedure. By doing so, we examined the sampling efficiency, distribution of the 4 modes, early-stage immune efficacy ($\varepsilon$ at day 7) and maximum immune efficacy ($\varepsilon_{\max}$), and show our results in Figure S16.

Second, we examine the combinatory effects of the parameters if they are randomly sampled. We also build upon our sampling method, and allow each previously fixed parameter to be sampled within two-fold of their original value ($P \in [P_0/2, 2P_0]$). Since for a given virus, virulence $\gamma$ should not change dramatically, we fix the virulence-related parameters, $k_{infect}$, $r_H$, $d_H$, $d_{If}$ and $N_1$. As every other parameter being randomly sampled, we examined the sampling efficiency, distribution of 4 modes,

immune kinetics, and their immune efficacy dynamics in Figure S17. Results show that the random sampling effects of these previously fixed parameters reduce the sampling efficiency and mode distribution, yet do not change our main conclusions regarding immune kinetics and immune efficacy.

# 7. Immune Memory Protection and Vaccine Efficacy

### 7.1 Vaccine protection rates in simulation

We use the parameter sets of mode 1~4 (Table S4) to compute the vaccine protection rates and analyze the impacts of different factors in Figure S21B-C. The fractions of samples without infection process and whose IL-6 level is below 2000 pg/mL out of 1,000 samples, are defined as the full protection rate and severe prevention rate, respectively. 1000 samples of initial memory levels (CD8+$T_M$ and Ab) are generated following the distribution given in Figure S21B, and the viral load and IL-6 dynamics are used to determine whether one is fully protected or have mild/severe breakthrough infection. The simulation results (Figure S21C) suggest that both virulence and initial inoculum of the virus dramatically reduce the effectiveness of vaccine, as the antibody affinity increases the efficacies.

# 8. Clinical indicators for immune efficacy

For the convenience of clinical measurement, we propose an alternative for the immune efficacy which is defined in patient's lung area, by simple measurements of patient's hemogram data. We have the definition of $\epsilon(t) \equiv \epsilon_c(t)\epsilon_k(t)$ in the main text, where $\epsilon_k(t) \equiv f_{eff}^{APC} k_1^{kill}[APC^l] + f_{eff}^{APC} k_2^{kill}[APC^u] + f_{eff}^{NK} k_3^{kill}[NK] + k_4^{kill}[CTL] + d_{If}$ and $\varepsilon_c(t) \equiv f_{eff}^{APC} k_1^{clear}[APC^l] + f_{eff}^{APC} k_2^{clear}[APC^u] + k_3^{clear}[Neut] + k_4^{clear} A[Ig]$. Here we make weak correspondences between Monocytes and APCs, and

between lymphocytes and NK+CTL.

Firstly, we tried $E^* \equiv ([Neut] + [Mono]) \times ([Lymph] + [Mono])$ as the most straight forward way to reflect immune efficacy. We found in Figure S26A (right panel) that $E^*$ is higher in critical patients compared mild/moderate and severe groups. In critical patients, the course of disease tends to be longer, suggesting weaker immune efficacy. However, due to their elevated neutrophil counts, $E^*$ turned out to be greater in critical patients, compared to mild/moderate and severe patients. Thus $E^*$ does not serve as a good indicator for the patients' immune efficacy.

Next, we defined $\varepsilon^* \equiv (Neut\% + Mono\%) \times (Lymph\% + Mono\%)$ as the clinical immune efficacy. The results are also in Figure S26A (left panel). This indicator of patient's immune efficacy can be reformulated in the following two ways: $\varepsilon^* \approx (Neut\% + Mono\%) \times (1 - Neut\%)$, given that neutrophil count takes up more than 50% of WBC, $\varepsilon^*$ reflects the negative correlation between inflammation (neutrophil counts) and immune efficacy; $\varepsilon^* \approx (1 - Lymph\%) \times (Lymph\% + Mono\%)$ , similarly, lymphocyte percentage are usually under 50%, therefore decrease in lymphocyte (lymphopenia) corresponds to decrease in immune efficacy. Shown in the distribution in Figure S26B (upper panel), this key indicator distinguishes between the three groups.

Particularly, given the fact that $Neut\% + Mono\% + Lymph\% \approx 1$, it can be shown that $\varepsilon^* \approx Mono\% + Neut\% \times Lymph\%$. As monocytes take up only around 10% of the total peripheral blood WBC, the variation in itself among the patients is rather minor, thus we propose $\varepsilon^\# \equiv Neut\% \times Lymph\%$ also proves to be a good indicator heuristically (Figure S26A middle, S26B middle).

## SI References


1 Abbas Abul, K., Trotta, E., Dimitre, R. S., Marson, A. & Bluestone Jeffrey, A. Revisiting IL-2: Biology and therapeutic prospects. *Science Immunology* **3**, eaat1482, doi:10.1126/sciimmunol.aat1482 (2018).

2 Ross, S. H. & Cantrell, D. A. Signaling and Function of Interleukin-2 in T Lymphocytes. *Annu Rev Immunol* **36**, 411-433, doi:10.1146/annurev-immunol-042617-053352 (2018).

3 Spellberg, B. & Edwards, J. E., Jr. Type 1/Type 2 Immunity in Infectious Diseases. *Clinical Infectious Diseases* **32**, 76-102, doi:10.1086/317537 (2001).

4 Zhou, F. *et al.* Clinical course and risk factors for mortality of adult inpatients with COVID-19 in Wuhan, China: a retrospective cohort study. *The Lancet* **395**, 1054-1062, doi:10.1016/s0140-6736(20)30566-3 (2020).

5 Ouyang, W. & O'Garra, A. IL-10 Family Cytokines IL-10 and IL-22: from Basic Science to Clinical Translation. *Immunity* **50**, 871-891, doi:10.1016/j.immuni.2019.03.020 (2019).

6 Kalliolias, G. D. & Ivashkiv, L. B. TNF biology, pathogenic mechanisms and emerging therapeutic strategies. *Nature Reviews Rheumatology* **12**, 49-62, doi:10.1038/nrrheum.2015.169 (2016).

7 Cavalcanti, Y. V. N., Brelaz, M. C. A., Neves, J. K. d. A. L., Ferraz, J. C. & Pereira, V. R. A. Role of TNF-Alpha, IFN-Gamma, and IL-10 in the Development of Pulmonary Tuberculosis. *Pulmonary Medicine* **2012**, 745483, doi:10.1155/2012/745483 (2012).

8 Ivashkiv, L. B. IFNγ: signalling, epigenetics and roles in immunity, metabolism, disease and cancer immunotherapy. *Nature Reviews Immunology* **18**, 545-558, doi:10.1038/s41577-018-0029-z (2018).

9 Coomes, E. A. & Haghbayan, H. Interleukin-6 in Covid-19: A systematic review and <scp>meta-analysis</scp>. *Reviews in Medical Virology* **30**, 1-9, doi:10.1002/rmv.2141 (2020).

10 Del Valle, D. M. *et al.* An inflammatory cytokine signature predicts COVID-19 severity and survival. *Nature Medicine* **26**, 1636-1643, doi:10.1038/s41591-020-1051-9 (2020).

11 Anikeeva, N., Grosso, D., Flomenberg, N. & Sykulev, Y. Evaluating frequency and quality of pathogen-specific T cells. *Nature Communications* **7**, 13264, doi:10.1038/ncomms13264 (2016).

12 Sheahan, T. P. *et al.* Comparative therapeutic efficacy of remdesivir and combination lopinavir, ritonavir, and interferon beta against MERS-CoV. *Nat Commun* **11**, 222, doi:10.1038/s41467-019-13940-6 (2020).

13 Jenner, A. L. *et al.* COVID-19 virtual patient cohort suggests immune mechanisms driving disease outcomes. *PLoS Pathog* **17**, e1009753, doi:10.1371/journal.ppat.1009753 (2021).

14 Blanco-Melo, D. *et al.* Imbalanced Host Response to SARS-CoV-2 Drives Development of COVID-19. *Cell* **181**, 1036-1045.e1039, doi:10.1016/j.cell.2020.04.026 (2020).

15 Nagaoka, K. *et al.* Circulating Type I Interferon Levels in the Early Phase of COVID-19 Are Associated With the Development of Respiratory Failure. *Frontiers in Immunology* **13**, doi:10.3389/fimmu.2022.844304 (2022).

16 Aleksandr, I. *et al.* Interferon alpha-based combinations suppress SARS-CoV-2 infection in vitro and in vivo. *bioRxiv*, 2021.2001.2005.425331, doi:10.1101/2021.01.05.425331

(2021).

17 Schuhenn, J. *et al.* Differential interferon-α subtype induced immune signatures are associated with suppression of SARS-CoV-2 infection. *Proceedings of the National Academy of Sciences* **119**, e2111600119, doi:10.1073/pnas.2111600119 (2022).

18 Galani, I.-E. *et al.* Untuned antiviral immunity in COVID-19 revealed by temporal type I/III interferon patterns and flu comparison. *Nature Immunology* **22**, 32-40, doi:10.1038/s41590-020-00840-x (2021).

19 Lucas, C. *et al.* Longitudinal analyses reveal immunological misfiring in severe COVID-19. *Nature* **584**, 463-469, doi:10.1038/s41586-020-2588-y (2020).

20 Hadjadj, J. *et al.* Impaired type I interferon activity and inflammatory responses in severe COVID-19 patients. *Science* **369**, 718-724, doi:10.1126/science.abc6027 (2020).

21 Kaech, S. M., Wherry, E. J. & Ahmed, R. Effector and memory T-cell differentiation: implications for vaccine development. *Nat Rev Immunol* **2**, 251-262, doi:10.1038/nri778 (2002).

22 De Boer, R. J., Homann, D. & Perelson, A. S. Different Dynamics of CD4<sup>+</sup> and CD8<sup>+</sup> T Cell Responses During and After Acute Lymphocytic Choriomeningitis Virus Infection. *The Journal of Immunology* **171**, 3928, doi:10.4049/jimmunol.171.8.3928 (2003).

23 Perelson, A. S. Modelling viral and immune system dynamics. *Nature Reviews Immunology* **2**, 28-36, doi:10.1038/nri700 (2002).

24 Kim, K. S. *et al.* A quantitative model used to compare within-host SARS-CoV-2, MERS-CoV, and SARS-CoV dynamics provides insights into the pathogenesis and treatment of SARS-CoV-2. *PLOS Biology* **19**, e3001128, doi:10.1371/journal.pbio.3001128 (2021).

25 Kim, K. S. *et al.* A quantitative model used to compare within-host SARS-CoV-2, MERS-CoV, and SARS-CoV dynamics provides insights into the pathogenesis and treatment of SARS-CoV-2. *PLoS Biol* **19**, e3001128-e3001128, doi:10.1371/journal.pbio.3001128 (2021).

26 van den Driessche, P. & Watmough, J. Reproduction numbers and sub-threshold endemic equilibria for compartmental models of disease transmission. *Mathematical Biosciences* **180**, 29-48, doi:https://doi.org/10.1016/S0025-5564(02)00108-6 (2002).

27 van den Driessche, P. Reproduction numbers of infectious disease models. *Infectious Disease Modelling* **2**, 288-303, doi:https://doi.org/10.1016/j.idm.2017.06.002 (2017).

28 Iannacone, M. & Guidotti, L. G. Immunobiology and pathogenesis of hepatitis B virus infection. *Nature Reviews Immunology* **22**, 19-32, doi:10.1038/s41577-021-00549-4 (2021).

29 Liao, M. *et al.* Single-cell landscape of bronchoalveolar immune cells in patients with COVID-19. *Nat Med* **26**, 842-844, doi:10.1038/s41591-020-0901-9 (2020).

30 Crapo, J. D., Barry, B. E., Gehr, P., Bachofen, M. & Weibel, E. R. Cell number and cell characteristics of the normal human lung. *Am Rev Respir Dis* **126**, 332-337, doi:10.1164/arrd.1982.126.2.332 (1982).

31 Liao, M. *et al.* Single-cell landscape of bronchoalveolar immune cells in patients with COVID-19. *Nature Medicine* **26**, 842-844, doi:10.1038/s41591-020-0901-9 (2020).

32 Armstrong, J. D., Gluck, E. H., Crapo, R. O., Jones, H. A. & Hughes, J. M. Lung tissue volume estimated by simultaneous radiographic and helium dilution methods. *Thorax* **37**, 676-

679, doi:10.1136/thx.37.9.676 (1982).

33 Helton, J. C. & Davis, F. J. Latin hypercube sampling and the propagation of uncertainty in analyses of complex systems. *Reliability Engineering & System Safety* **81**, 23-69, doi:10.1016/S0951-8320(03)00058-9 (2002).

34 Virtanen, P. *et al.* SciPy 1.0: fundamental algorithms for scientific computing in Python. *Nature Methods* **17**, 261-272, doi:10.1038/s41592-019-0686-2 (2020).

35 Jolliffe, I. T. & Cadima, J. Principal component analysis: a review and recent developments. *Philosophical Transactions of the Royal Society A: Mathematical, Physical and Engineering Sciences* **374**, 20150202, doi:10.1098/rsta.2015.0202 (2016).

36 Sanders, J. M., Monogue, M. L., Jodlowski, T. Z. & Cutrell, J. B. Pharmacologic Treatments for Coronavirus Disease 2019 (COVID-19): A Review. *JAMA* **323**, 1824-1836, doi:10.1001/jama.2020.6019 (2020).

37 Lee, J. S. & Shin, E.-C. The type I interferon response in COVID-19: implications for treatment. *Nature Reviews Immunology* **20**, 585-586, doi:10.1038/s41577-020-00429-3 (2020).

38 Zhao, Y. *et al.* Critical slowing down and attractive manifold: A mechanism for dynamic robustness in the yeast cell-cycle process. *Physical Review E* **101**, 042405, doi:10.1103/PhysRevE.101.042405 (2020).

39 Cai, J. *et al.* The Neutrophil-to-Lymphocyte Ratio Determines Clinical Efficacy of Corticosteroid Therapy in Patients with COVID-19. *Cell Metabolism*, doi:10.1016/j.cmet.2021.01.002 (2021).

40 Russell, B. *et al.* Associations between immune-suppressive and stimulating drugs and novel COVID-19-a systematic review of current evidence. *Ecancermedicalscience* **14**, 1022-1022, doi:10.3332/ecancer.2020.1022 (2020).

41 Beigel, J. H. *et al.* Remdesivir for the Treatment of Covid-19 — Final Report. *New England Journal of Medicine* **383**, 1813-1826, doi:10.1056/NEJMoa2007764 (2020).

42 Monk, P. D. *et al.* Safety and efficacy of inhaled nebulised interferon beta-1a (SNG001) for treatment of SARS-CoV-2 infection: a randomised, double-blind, placebo-controlled, phase 2 trial. *The Lancet Respiratory Medicine* **9**, 196-206, doi:10.1016/S2213-2600(20)30511-7 (2021).

43 Chen, P. *et al.* SARS-CoV-2 Neutralizing Antibody LY-CoV555 in Outpatients with Covid-19. *New England Journal of Medicine* **384**, 229-237, doi:10.1056/NEJMoa2029849 (2020).

44 Duan, K. *et al.* Effectiveness of convalescent plasma therapy in severe COVID-19 patients. *Proceedings of the National Academy of Sciences* **117**, 9490, doi:10.1073/pnas.2004168117 (2020).

45 The-RECOVERY-Collaborative-Group. Dexamethasone in Hospitalized Patients with Covid-19 — Preliminary Report. *New England Journal of Medicine*, doi:10.1056/NEJMoa2021436 (2020).

46 Shen, C. *et al.* Treatment of 5 Critically Ill Patients With COVID-19 With Convalescent Plasma. *JAMA* **323**, 1582-1589, doi:10.1001/jama.2020.4783 (2020).

47 Tan, A. T. *et al.* Early induction of functional SARS-CoV-2-specific T cells associates with rapid viral clearance and mild disease in COVID-19 patients. *Cell Reports* **34**, doi:ARTN 108728
10.1016/j.celrep.2021.108728 (2021).

48 Néant, N. *et al.* Modeling SARS-CoV-2 viral kinetics and association with mortality in hospitalized patients from the French COVID cohort. *Proceedings of the National Academy of Sciences* **118**, e2017962118, doi:10.1073/pnas.2017962118 (2021).

49 Goyal, A., Cardozo-Ojeda, E. F. & Schiffer, J. T. Potency and timing of antiviral therapy as determinants of duration of SARS-CoV-2 shedding and intensity of inflammatory response. *Science Advances* **6**, eabc7112, doi:10.1126/sciadv.abc7112 (2020).

50 Liu, Y. *et al.* Viral dynamics in mild and severe cases of COVID-19. *The Lancet Infectious Diseases* **20**, 656-657, doi:10.1016/s1473-3099(20)30232-2 (2020).

51 Hancioglu, B., Swigon, D. & Clermont, G. A dynamical model of human immune response to influenza A virus infection. *J Theor Biol* **246**, 70-86, doi:10.1016/j.jtbi.2006.12.015 (2007).

52 Jenner, A. L. *et al.* COVID-19 virtual patient cohort suggests immune mechanisms driving disease outcomes. *PLOS Pathogens* **17**, e1009753, doi:10.1371/journal.ppat.1009753 (2021).

53 Goyal, A., Cardozo-Ojeda, E. F. & Schiffer, J. T. Potency and timing of antiviral therapy as determinants of duration of SARS-CoV-2 shedding and intensity of inflammatory response. *Sci Adv* **6**, doi:10.1126/sciadv.abc7112 (2020).

54 Bar-On, Y. M., Flamholz, A., Phillips, R. & Milo, R. SARS-CoV-2 (COVID-19) by the numbers. *eLife* **9**, e57309, doi:10.7554/eLife.57309 (2020).

55 De Boer Rob, J. *et al.* Recruitment Times, Proliferation, and Apoptosis Rates during the CD8+ T-Cell Response to Lymphocytic Choriomeningitis Virus. *Journal of Virology* **75**, 10663-10669, doi:10.1128/JVI.75.22.10663-10669.2001 (2001).

56 Price, I. *et al.* The inflammatory response to influenza A virus (H1N1): An experimental and mathematical study. *J Theor Biol* **374**, 83-93, doi:10.1016/j.jtbi.2015.03.017 (2015).

57 Lahoz-Beneytez, J. *et al.* Human neutrophil kinetics: modeling of stable isotope labeling data supports short blood neutrophil half-lives. *Blood* **127**, 3431-3438, doi:10.1182/blood-2016-03-700336 (2016).

58 De Boer, R. J., Homann, D. & Perelson, A. S. Different Dynamics of CD4+ and CD8+ T Cell Responses During and After Acute Lymphocytic Choriomeningitis Virus Infection. *The Journal of Immunology* **171**, 3928-3935, doi:10.4049/jimmunol.171.8.3928 (2003).

59 Dan, J. M. *et al.* Immunological memory to SARS-CoV-2 assessed for up to 8 months after infection. *Science* **371**, eabf4063, doi:10.1126/science.abf4063 (2021).

60 Bromage, E., Stephens, R. & Hassoun, L. The third dimension of ELISPOTs: quantifying antibody secretion from individual plasma cells. *J Immunol Methods* **346**, 75-79, doi:10.1016/j.jim.2009.05.005 (2009).

61 Andraud, M. *et al.* Living on three time scales: the dynamics of plasma cell and antibody populations illustrated for hepatitis a virus. *PLoS computational biology* **8**, e1002418-e1002418, doi:10.1371/journal.pcbi.1002418 (2012).

62 Palacios, R. a. B., Ana Paula and Albuquerque, Camila Santos Nascimento and Patiño, Elizabeth González and Santos, Joane do Prado and Tilli Reis Pessoa Conde, Mônica and Piorelli, Roberta de Oliveira and Pereira Júnior, Luiz Carlos and Raboni, Sonia Mara and Ramos, Fabiano and Sierra Romero, Gustavo Adolfo and Leal, Fábio Eudes and Camargo, Luis Fernando Aranha and Aoki, Francisco Hideo and Coelho, Eduardo Barbosa and Oliveira, Danise Senna and Fontes, Cor Jesus Fernandes and Pileggi, Gecilmara Cristina Salviato and Oliveira, Ana Lúcia Lyrio de and Siqueira, André Machado de and Oliveira,

Danielle Bruna Leal de and Botosso, Viviane Fongaro and Zeng, Gang and Xin, Qianqian and Teixeira, Mauro Martins and Nogueira, Maurício Lacerda and Kallas, Esper Georges, . *Efficacy and Safety of a COVID-19 Inactivated Vaccine in Healthcare Professionals in Brazil: The PROFISCOV Study* (2021).

63 Folegatti, P. M. *et al.* Safety and immunogenicity of the ChAdOx1 nCoV-19 vaccine against SARS-CoV-2: a preliminary report of a phase 1/2, single-blind, randomised controlled trial. *Lancet* **396**, 467-478, doi:10.1016/S0140-6736(20)31604-4 (2020).

64 Voysey, M. *et al.* Safety and efficacy of the ChAdOx1 nCoV-19 vaccine (AZD1222) against SARS-CoV-2: an interim analysis of four randomised controlled trials in Brazil, South Africa, and the UK. *Lancet* **397**, 99-111, doi:10.1016/S0140-6736(20)32661-1 (2021).

65 Lopez Bernal, J. *et al.* Effectiveness of Covid-19 Vaccines against the B.1.617.2 (Delta) Variant. *New England Journal of Medicine* **385**, 585-594, doi:10.1056/NEJMoa2108891 (2021).

66 Andrews, N. *et al.* Covid-19 Vaccine Effectiveness against the Omicron (B.1.1.529) Variant. *N Engl J Med* **386**, 1532-1546, doi:10.1056/NEJMoa2119451 (2022).

67 Sahin, U. *et al.* BNT162b2 vaccine induces neutralizing antibodies and poly-specific T cells in humans. *Nature* **595**, 572-577, doi:10.1038/s41586-021-03653-6 (2021).

68 Polack, F. P. *et al.* Safety and Efficacy of the BNT162b2 mRNA Covid-19 Vaccine. *N Engl J Med* **383**, 2603-2615, doi:10.1056/NEJMoa2034577 (2020).

69 Angyal, A. *et al.* T-cell and antibody responses to first BNT162b2 vaccine dose in previously infected and SARS-CoV-2-naive UK health-care workers: a multicentre prospective cohort study. *Lancet Microbe* **3**, e21-e31, doi:10.1016/S2666-5247(21)00275-5 (2022).

70 Chodick, G. *et al.* Assessment of Effectiveness of 1 Dose of BNT162b2 Vaccine for SARS-CoV-2 Infection 13 to 24 Days After Immunization. *JAMA Netw Open* **4**, e2115985, doi:10.1001/jamanetworkopen.2021.15985 (2021).

71 Jackson, L. A. *et al.* An mRNA Vaccine against SARS-CoV-2 - Preliminary Report. *N Engl J Med* **383**, 1920-1931, doi:10.1056/NEJMoa2022483 (2020).

72 Baden, L. R. *et al.* Efficacy and Safety of the mRNA-1273 SARS-CoV-2 Vaccine. *N Engl J Med* **384**, 403-416, doi:10.1056/NEJMoa2035389 (2021).

73 Sadoff, J. *et al.* Interim Results of a Phase 1-2a Trial of Ad26.COV2.S Covid-19 Vaccine. *N Engl J Med* **384**, 1824-1835, doi:10.1056/NEJMoa2034201 (2021).
