# Supplementary material for: Dynamical modelling of viral infection and cooperative immune protection in COVID-19 patients
Source: PLoS Comput Biol. 2023 Sep 1;19(9):e1011383. doi: 10.1371/journal.pcbi.1011383 (PMC10501599; doi:10.1371/journal.pcbi.1011383)
Supplement: S1 Fig — (PDF) [file pcbi.1011383.s002.pdf]

Figure S1

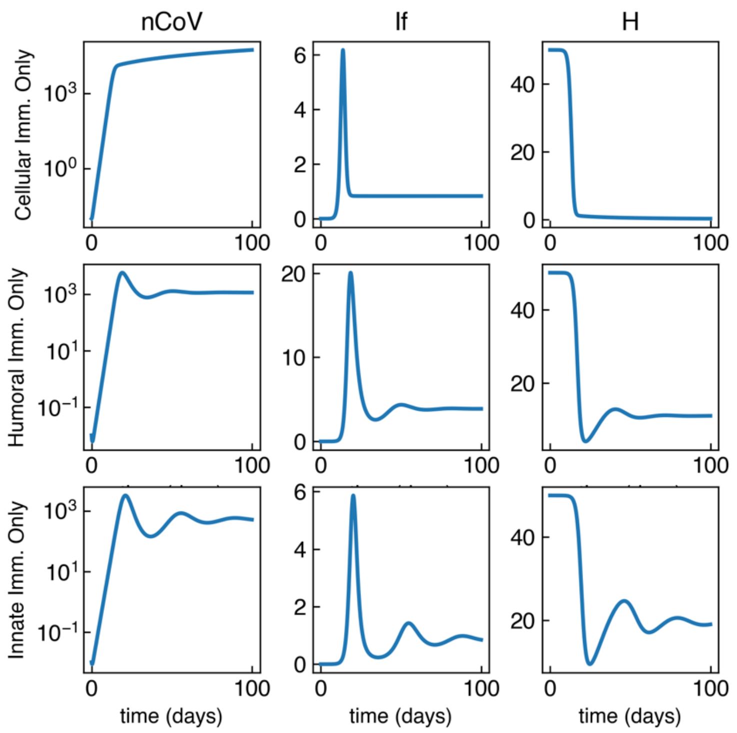

Figure S1. Simulation of time course of viral dynamics and immune response under extreme cases where only one arm of immunity exists. Upper Panel: viral load unlimitedly grows if only cellular immune response is present, as $\epsilon_c = 0$. Middle Panel: viral load will increase to high level and plateau if only humoral immunity exists. Lower Panel: viral load will increase and plateau if only innate immunity exists. In the middle and lower panel, as ϵ_k and ϵ_c are both non-zero, with the decrease in healthy epithelial cell $[H]$, R_t will eventually reach 1, where viral load stops to increase.
