# Supplementary material for: Dynamical modelling of viral infection and cooperative immune protection in COVID-19 patients
Source: PLoS Comput Biol. 2023 Sep 1;19(9):e1011383. doi: 10.1371/journal.pcbi.1011383 (PMC10501599; doi:10.1371/journal.pcbi.1011383)
Supplement: S2 Fig — (PDF) [file pcbi.1011383.s003.pdf]

Figure S2

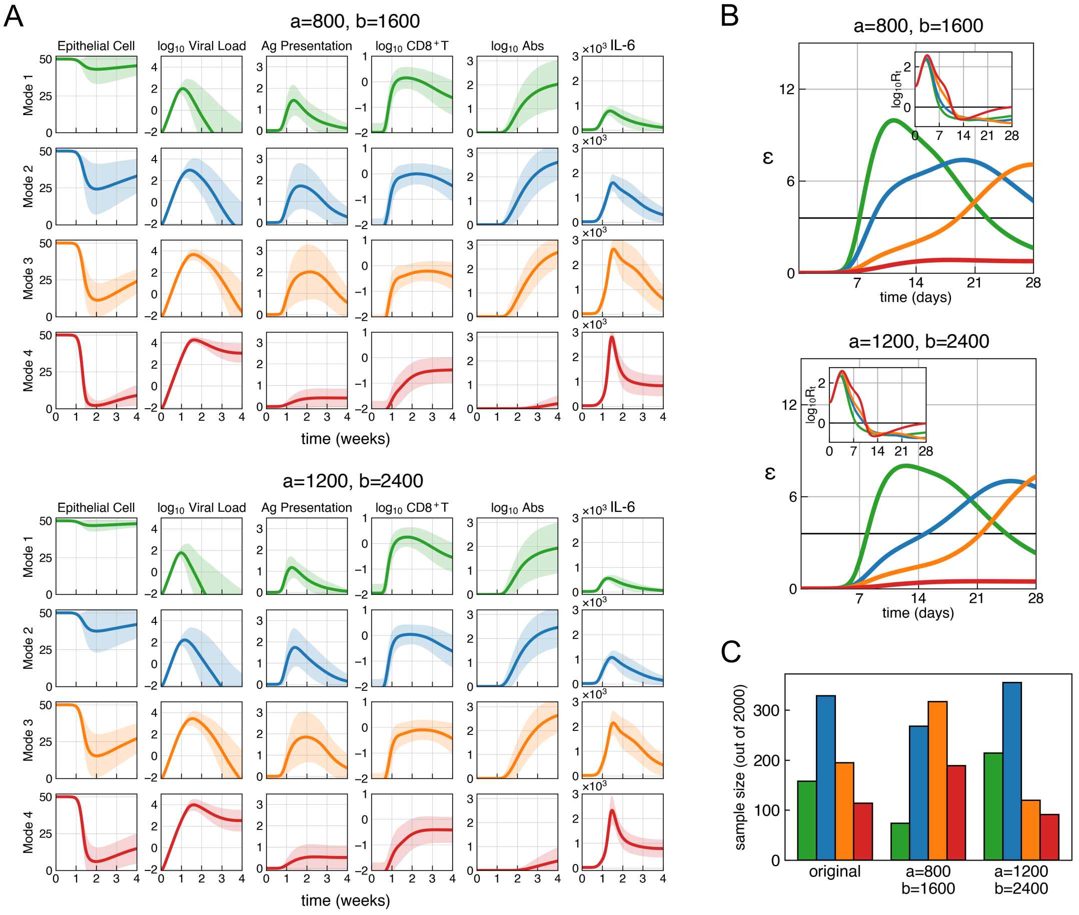

Figure S2. Choice of IL-6 boundary values for classification does not change the immune response.

(A) Immune response dynamics when the four modes are classified based on peak IL-6 level: Mode 1 < a < Mode 2 < b < Mode 3 & Mode 4.

(B) Across Mode 1 – 3, in more severe cases, the immune efficacy responds slower to viral challenge. In Mode 4, the immune efficacy cannot rise above γ, resulting in chronic infection.

(C) The change in boundary values only change the relative distribution of the four modes during sampling process.
