# Supplementary material for: Dynamical modelling of viral infection and cooperative immune protection in COVID-19 patients
Source: PLoS Comput Biol. 2023 Sep 1;19(9):e1011383. doi: 10.1371/journal.pcbi.1011383 (PMC10501599; doi:10.1371/journal.pcbi.1011383)
Supplement: S3 Fig — (PDF) [file pcbi.1011383.s004.pdf]

Figure S3

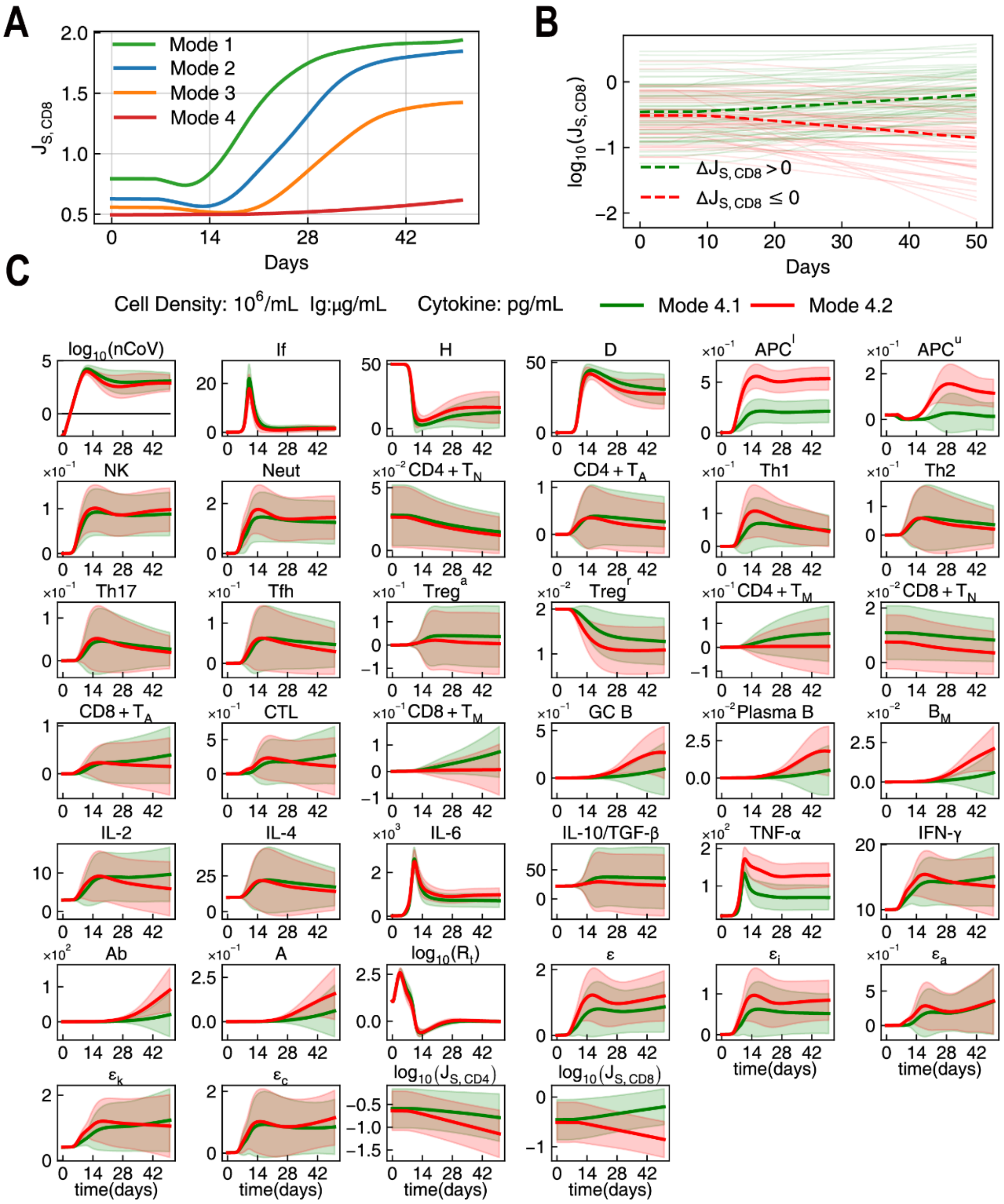

Figure S3. T cell supply fluxes and classification of Mode 4.

(A) Averaged time course of CD8+ T cell supply fluxes of mode 1~4.

(B) Time courses of CD8+ T cell supply fluxes in mode 4 sampling results, which are classified into two

subgroups. Mode 4.1 is defined as CD8+ T cell exhaustion ($\Delta J_{S,CD8} \leq 0$) and colored in red, while mode 4.2 has enough CD8+ T cell supply ($\Delta J_{S,CD8} > 0$) and colored in green, see details in section 2.3 in SI. Dashed lines are the average time courses of each subgroup.

(C) Mean±std time courses of two subgroups in mode 4.
