# Supplementary material for: Dynamical modelling of viral infection and cooperative immune protection in COVID-19 patients
Source: PLoS Comput Biol. 2023 Sep 1;19(9):e1011383. doi: 10.1371/journal.pcbi.1011383 (PMC10501599; doi:10.1371/journal.pcbi.1011383)
Supplement: S4 Fig — (PDF) [file pcbi.1011383.s005.pdf]

Figure S4

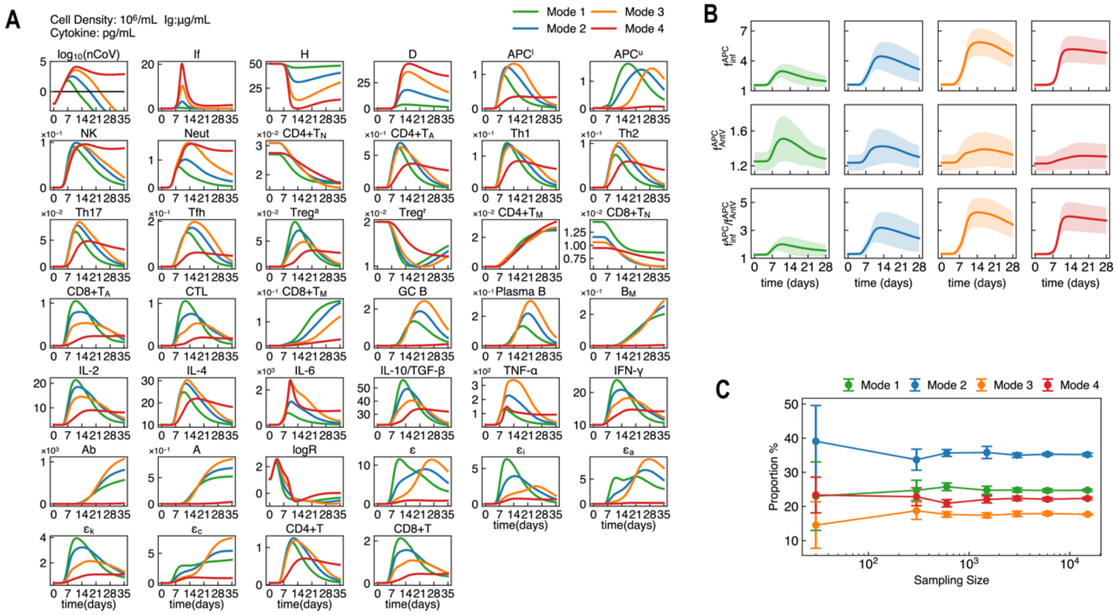

Figure S4. Sampling results.

(A) Averaged time course of the three immune response modes and of the asymptomatic patients against SARS-CoV-2 infection.

(B) APC's inflammatory and antiviral response are induced differently among the four modes. From Mode 1 to Mode 4, the inflammatory response increases, yet the antiviral response decreases.

(C) Sample results converge upon sample size of 1500.
