# Supplementary material for: Dynamical modelling of viral infection and cooperative immune protection in COVID-19 patients
Source: PLoS Comput Biol. 2023 Sep 1;19(9):e1011383. doi: 10.1371/journal.pcbi.1011383 (PMC10501599; doi:10.1371/journal.pcbi.1011383)
Supplement: S5 Fig — (PDF) [file pcbi.1011383.s006.pdf]

Figure S5

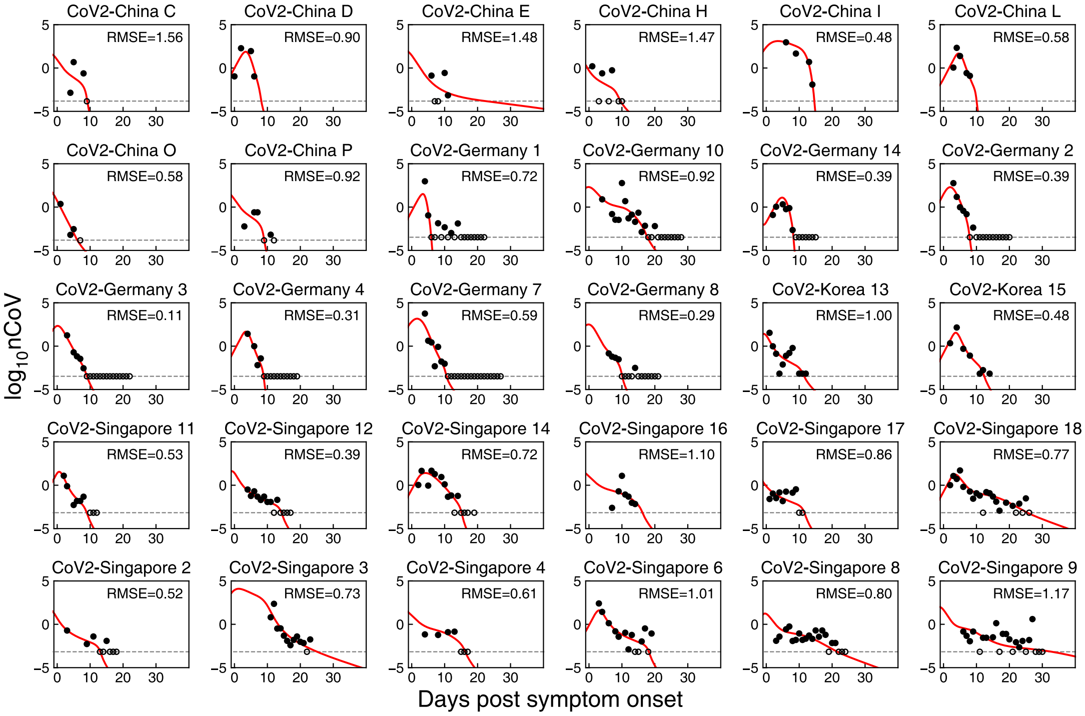

Figure S5. Comparison of viral load time courses between model simulation and clinical data.

Orange points represent viral load data from patients (1). Black curves are simulations that best match the clinical data. Gray dashed lines indicate the limit of detection for clinical viral load data. The root mean square error (RMSE) is shown in the upper right for each subfigure.

Reference

1. Kim KS, Ejima K, Iwanami S, Fujita Y, Ohashi H, Koizumi Y, et al. A quantitative model used to compare within-host SARS-CoV-2, MERS-CoV, and SARS-CoV dynamics provides insights into the pathogenesis and treatment of SARS-CoV-2. PLoS Biol. 2021;19(3):e3001128-e.
