# Supplementary material for: Dynamical modelling of viral infection and cooperative immune protection in COVID-19 patients
Source: PLoS Comput Biol. 2023 Sep 1;19(9):e1011383. doi: 10.1371/journal.pcbi.1011383 (PMC10501599; doi:10.1371/journal.pcbi.1011383)
Supplement: S6 Fig — (PDF) [file pcbi.1011383.s007.pdf]

Figure S6

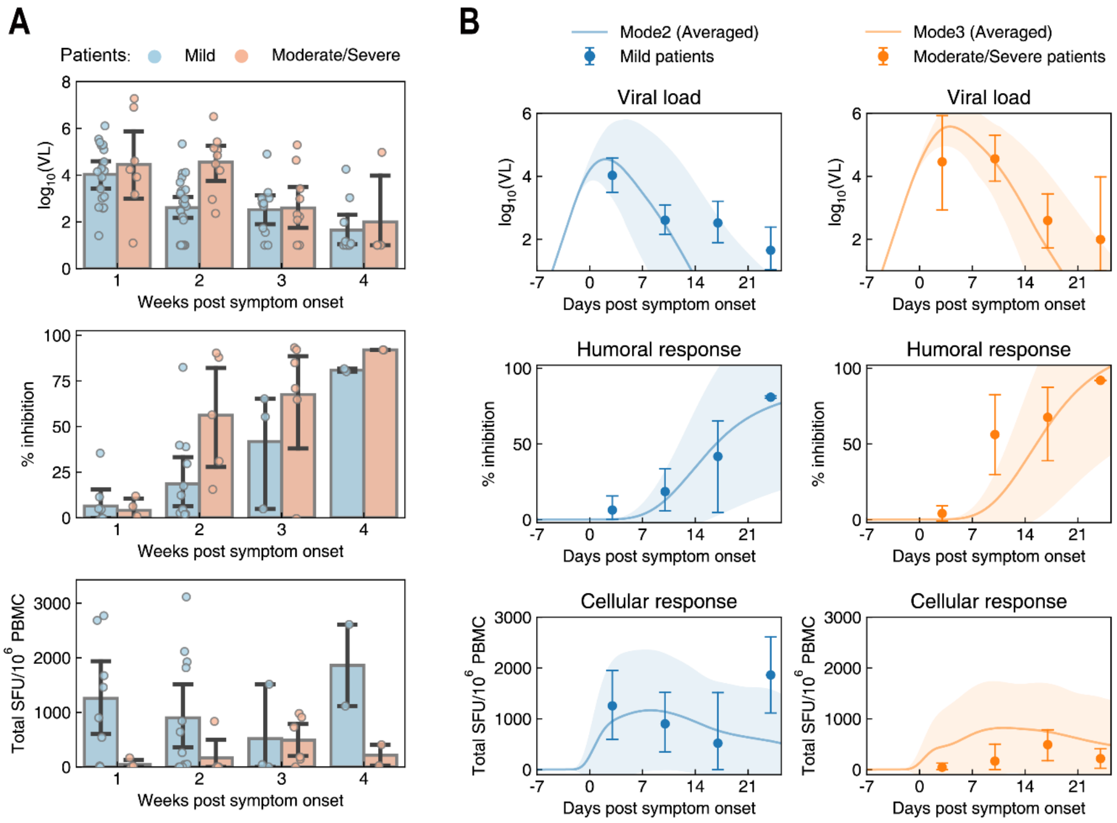

Figure S6. Comparison of viral load, humoral and cellular response between model simulation and COVID-19 patients' clinical data.

(A) Longitudinal viral load and immune responses data from (1).

(B) Comparison between clinical data and simulations (Mode 2 and 3). The time unit of clinical data are converted from weeks to days by the formula 'day = (week×7−4)'. The infection day is assumed to be 7 days before symptom onset. To compare clinical data with simulations, we defined '% inhibition' and 'Total SFU' of simulations as '% inhibition $= c_A A[\mathrm{Ig}]$' and 'TotalSFU $= c_T([\mathrm{Th1}] + [\mathrm{CTL}])$', where $c_A = 10$ and $c_T = 1500$.

Reference

1. Tan AT, Linster M, Tan CW, Le Bert N, Chia WN, Kunasegaran K, et al. Early induction of functional SARS-CoV-2-specific T cells associates with rapid viral clearance and mild disease in COVID-19 patients. Cell Reports. 2021;34(6).
