# Supplementary material for: Dynamical modelling of viral infection and cooperative immune protection in COVID-19 patients
Source: PLoS Comput Biol. 2023 Sep 1;19(9):e1011383. doi: 10.1371/journal.pcbi.1011383 (PMC10501599; doi:10.1371/journal.pcbi.1011383)
Supplement: S7 Fig — (PDF) [file pcbi.1011383.s008.pdf]

Figure S7

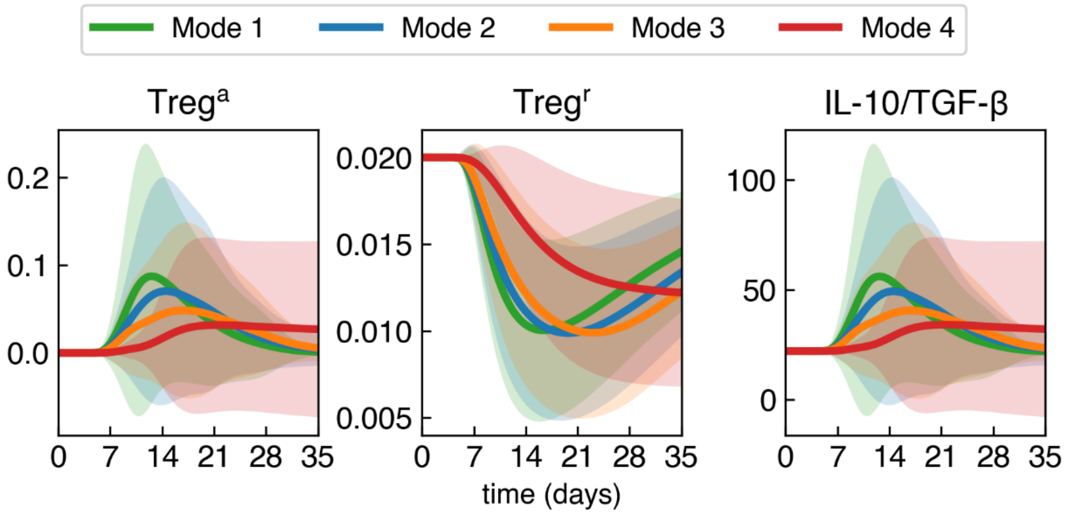

Figure S7. Severe cases (Mode 3 and 4) exhibited signatures of weaker immunosuppression, manifested by lower Treg activation level and suppressive cytokine IL-10 / TGF-β.
