# Supplementary material for: Dynamical modelling of viral infection and cooperative immune protection in COVID-19 patients
Source: PLoS Comput Biol. 2023 Sep 1;19(9):e1011383. doi: 10.1371/journal.pcbi.1011383 (PMC10501599; doi:10.1371/journal.pcbi.1011383)
Supplement: S8 Fig — (PDF) [file pcbi.1011383.s009.pdf]

Figure S8

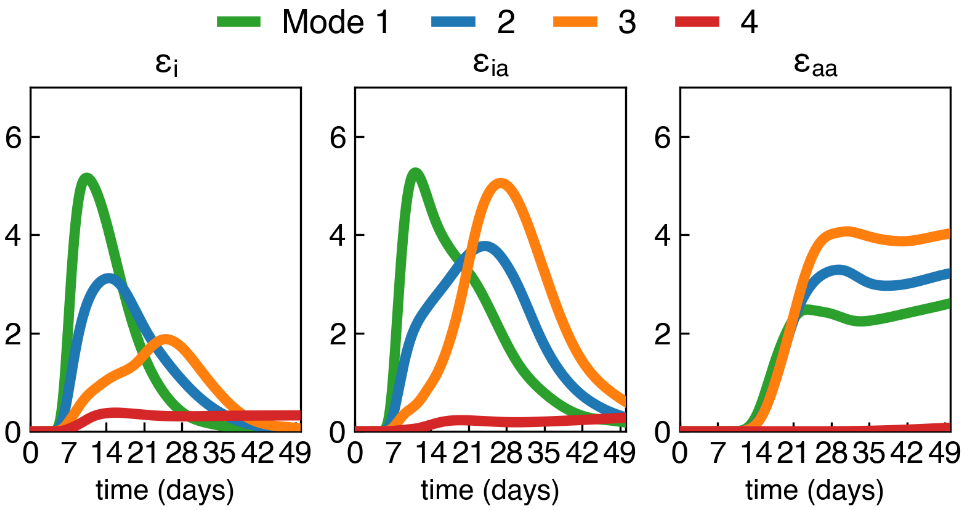

Figure S8. The dynamics of innate immune efficacy ϵ_i, adaptive immunity ϵ_{aa} and their cross term ϵ_{ia}.
