# Supplementary material for: Dynamical modelling of viral infection and cooperative immune protection in COVID-19 patients
Source: PLoS Comput Biol. 2023 Sep 1;19(9):e1011383. doi: 10.1371/journal.pcbi.1011383 (PMC10501599; doi:10.1371/journal.pcbi.1011383)
Supplement: S9 Fig — (PDF) [file pcbi.1011383.s010.pdf]

Figure S9

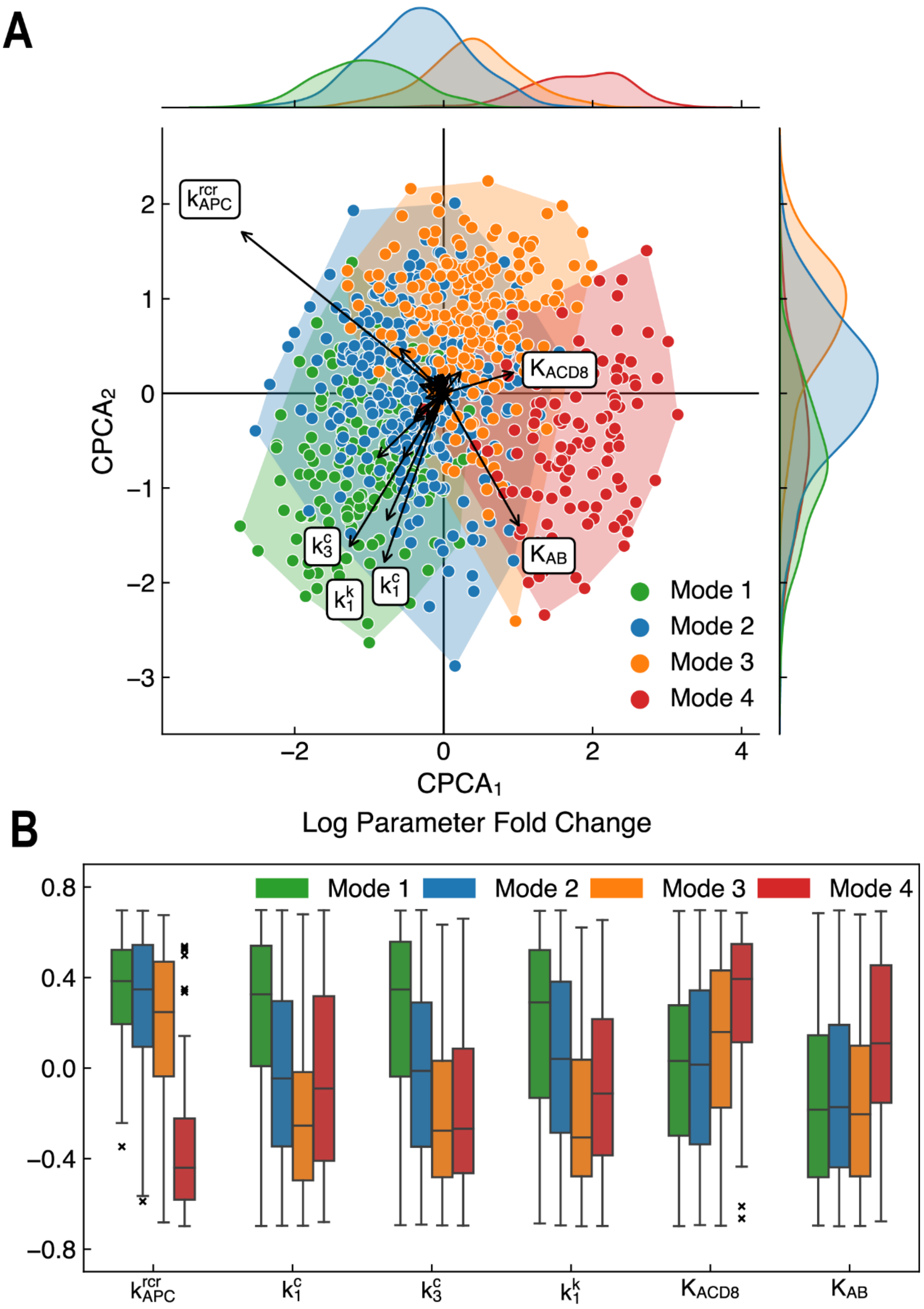
A
k_{APC}^{rcr}
K_{ACD8}
k_3^c
k_1^c
k_1^k
K_{AB}
Mode 1
Mode 2
Mode 3
Mode 4
$CPCA_1$
$CPCA_2$
B
Log Parameter Fold Change
Mode 1
Mode 2
Mode 3
Mode 4
k_{APC}^{rcr}
k_1^c
k_3^c
k_1^k
K_{ACD8}
K_{AB}

Figure S9. Class-based principal component analysis (CPCA) on the parameter samples.

(A) The distribution of the samples projected onto the 2-D parameter plane. The vectors represent the principal components of the samples.

(B) Statistics of the principal components, decentralized and normalized according to the sampling method. Major factors including APC recruitment rate, infected cell killing rate of APC, virus clearance rate of APC and neutrophils, APC antigen-presentation to CD8+ T cells and B cells distinguish the four modes.
