# Supplementary material for: Dynamical modelling of viral infection and cooperative immune protection in COVID-19 patients
Source: PLoS Comput Biol. 2023 Sep 1;19(9):e1011383. doi: 10.1371/journal.pcbi.1011383 (PMC10501599; doi:10.1371/journal.pcbi.1011383)
Supplement: S10 Fig — (PDF) [file pcbi.1011383.s011.pdf]

# Figure S10

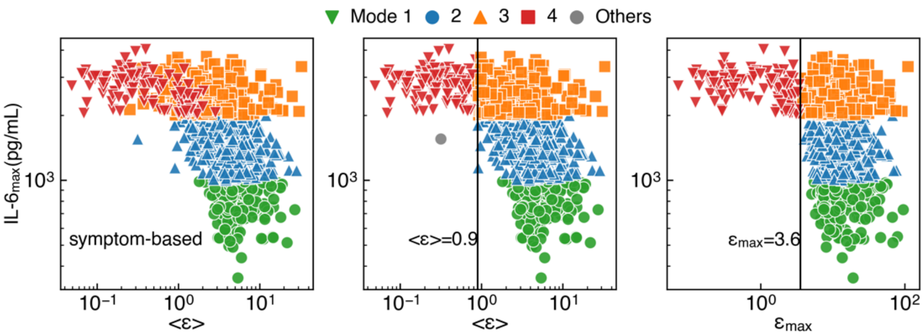


**Figure S10. Immune efficacy as a criterion to classify the modes.**

Immune efficacy could replace the original classification (Left) by final viral load to classify the simulations. Using averaged immune efficacy across the initial four weeks $<\epsilon>$ or maximum immune efficacy $\epsilon_{max}$ can both be used to classify the different modes.
