# Supplementary material for: Dynamical modelling of viral infection and cooperative immune protection in COVID-19 patients
Source: PLoS Comput Biol. 2023 Sep 1;19(9):e1011383. doi: 10.1371/journal.pcbi.1011383 (PMC10501599; doi:10.1371/journal.pcbi.1011383)
Supplement: S11 Fig — (PDF) [file pcbi.1011383.s012.pdf]

Figure S11

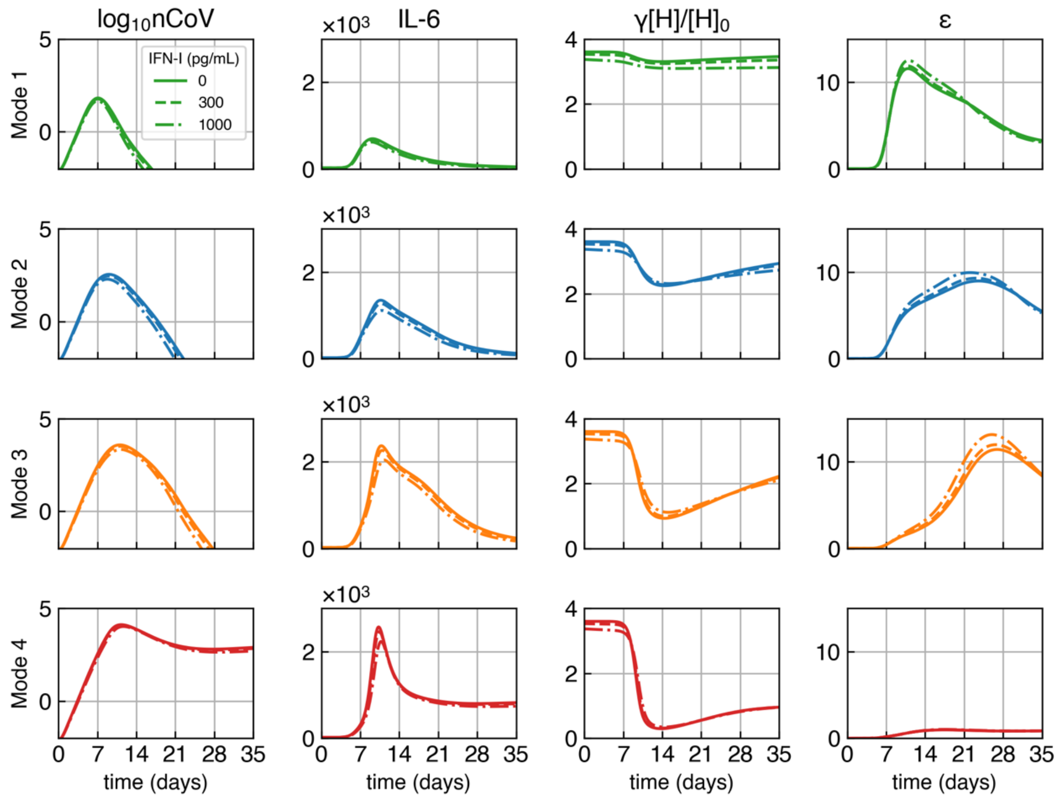

Figure S11. Immune dynamics of the 4 modes with different IFN-I levels. Increasing IFN-I level results in lower IL-6 and peak viral load, as well as increased immune efficacy ε and decreased virulence γ, but the change is minimal.
