# Supplementary material for: Dynamical modelling of viral infection and cooperative immune protection in COVID-19 patients
Source: PLoS Comput Biol. 2023 Sep 1;19(9):e1011383. doi: 10.1371/journal.pcbi.1011383 (PMC10501599; doi:10.1371/journal.pcbi.1011383)
Supplement: S12 Fig — (PDF) [file pcbi.1011383.s013.pdf]

Figure S12

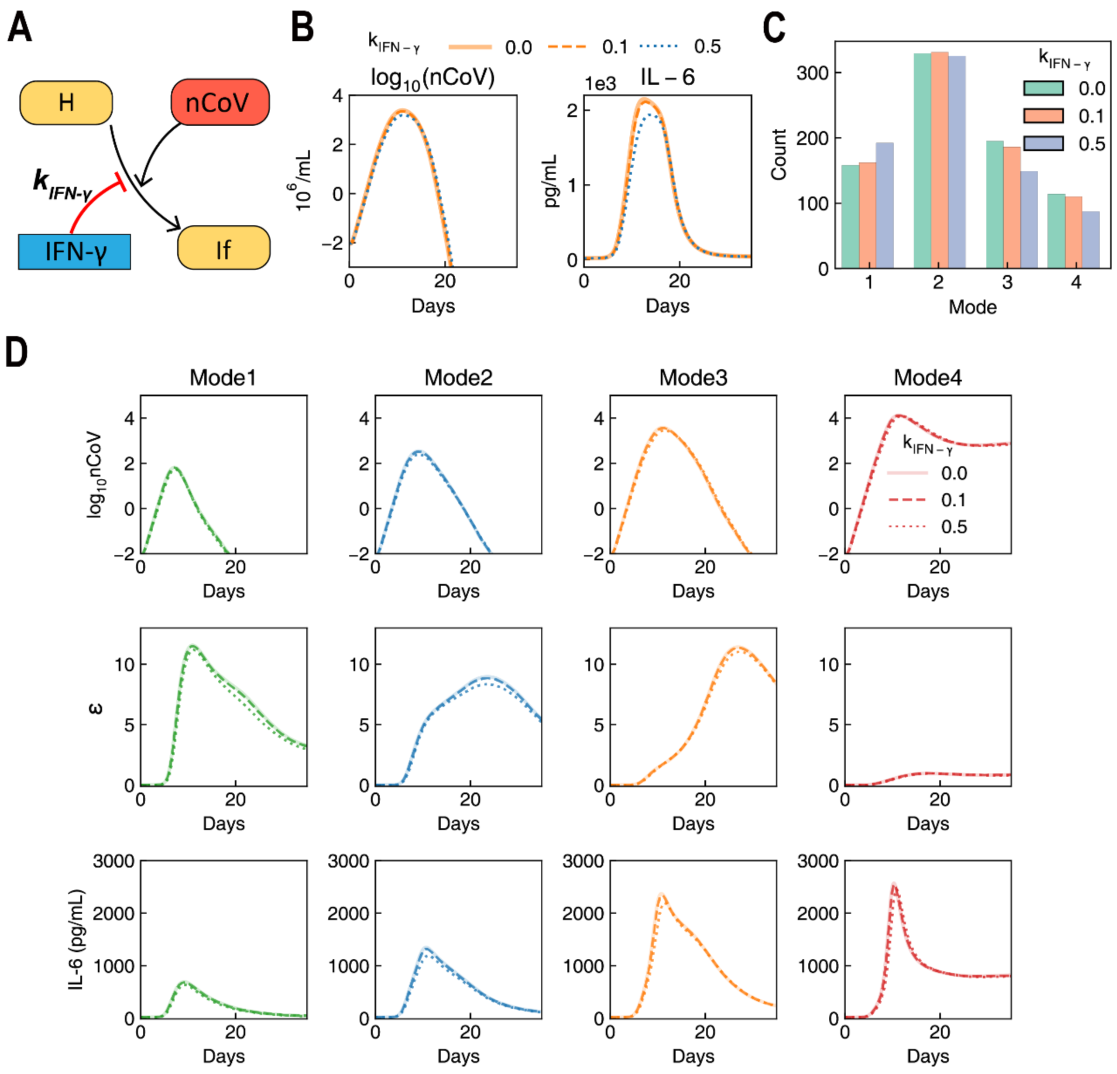

Figure S12. Simulations about the non-cytopathic effects of IFN-γ on viral infection and immune response.

(A) IFN-γ prohibits inhibits viral infection and kIFN-γ quantifies the strength of the inhibition prohibition.

(B) Time courses of viral load and IL-6 for a Mode 3 parameter set with/without $k_{IFN-γ}$.

(C) Distributions of Mode 1 – 4 with increased different $k_{IFN-γ}$.

(D) Averaged time courses of immune efficacy ε and IL-6 for Mode 1 - 4 with increased $k_{IFN-γ}$.
