# Supplementary material for: Dynamical modelling of viral infection and cooperative immune protection in COVID-19 patients
Source: PLoS Comput Biol. 2023 Sep 1;19(9):e1011383. doi: 10.1371/journal.pcbi.1011383 (PMC10501599; doi:10.1371/journal.pcbi.1011383)
Supplement: S13 Fig — (PDF) [file pcbi.1011383.s014.pdf]

Figure S13

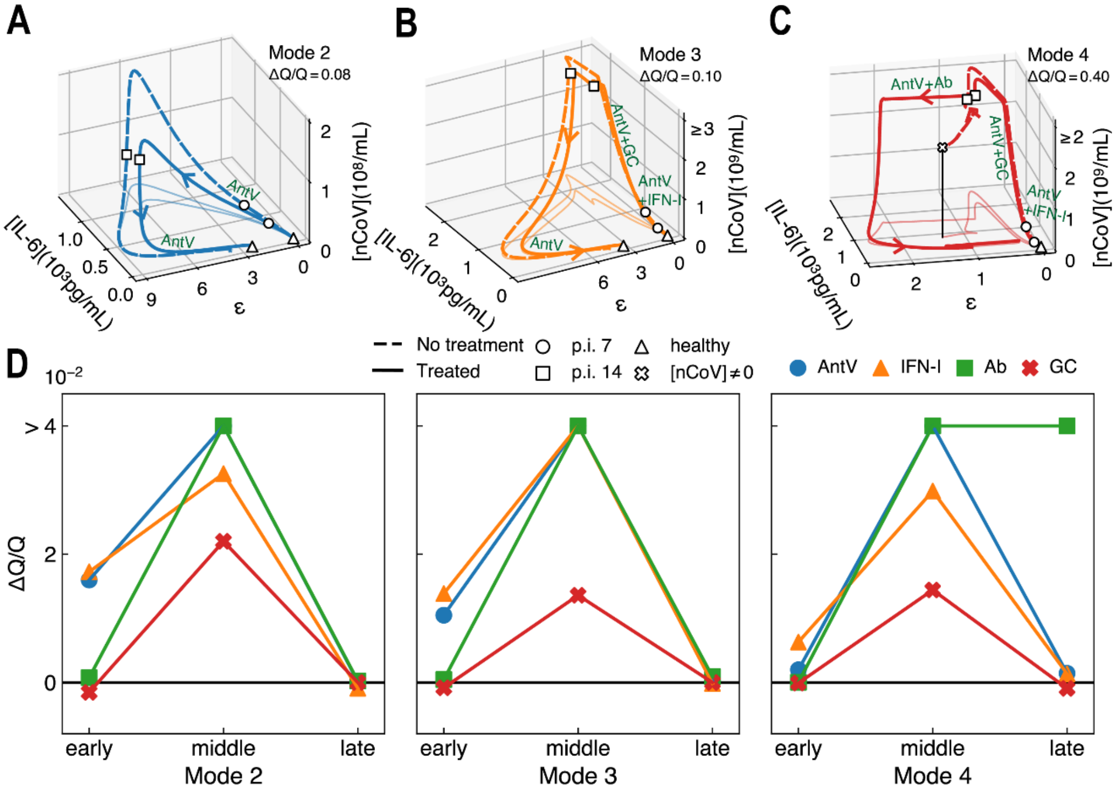

Figure S13. Treatment strategies for *in silico* patients.

(A~C) The immune response trajectories untreated (in dashed line) and the trajectories treated (in solid line) of mode 2 (A), mode 3 (B) and mode 4 patients (C). The trajectories are illustrated in the immune efficacy $\epsilon - [IL-6] - [nCoV]$ space.

(D) Effects of different drugs on improving patients' status (measure by $\Delta Q/Q$) in different immune response modes, when drug was used singly at different COVID-19 developmental stages. Early: 0~7 days p.i., middle: 7~14 days p.i., late: 14+ days p.i.. Sample-averaged (outliers excluded) value of $\Delta Q/Q$ is taken to assess the efficacy of the drugs. p.i. = post infection.
