# Supplementary material for: Dynamical modelling of viral infection and cooperative immune protection in COVID-19 patients
Source: PLoS Comput Biol. 2023 Sep 1;19(9):e1011383. doi: 10.1371/journal.pcbi.1011383 (PMC10501599; doi:10.1371/journal.pcbi.1011383)
Supplement: S14 Fig — (PDF) [file pcbi.1011383.s015.pdf]

Figure S14

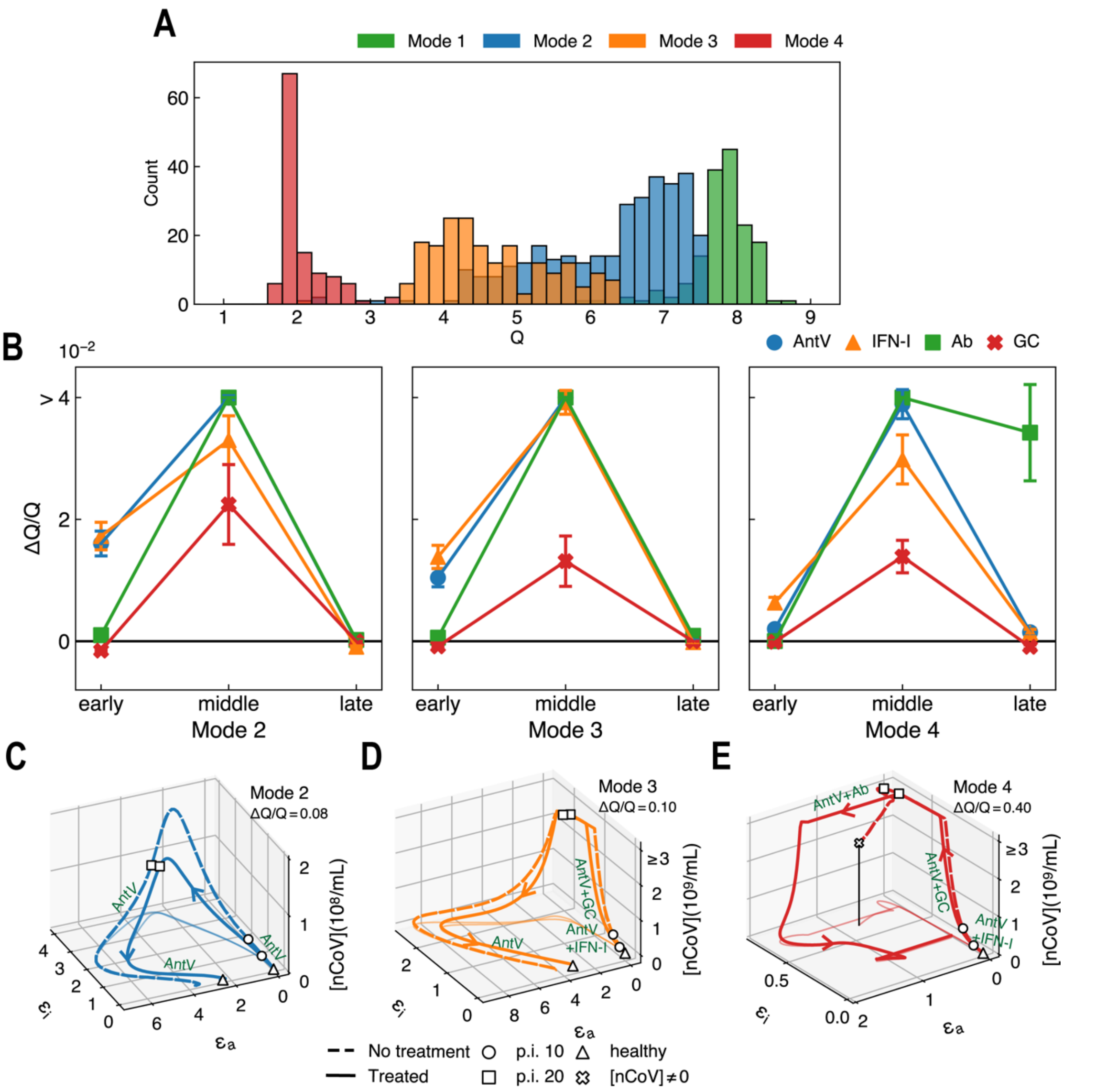

Figure S14. Q value distribution and sensitivity, and the ε_i-ε_a perspective of treatment strategies.

(A) Distribution of the Q value of Mode 1~4.

(B) Assessment in drug efficacy with Q-related parameters and treatment-related parameters randomly drawn from uniform distribution with amplitude of 50% and 20% of the original parameters, respectively.

(C~E) Sample-averaged trajectories of treated (solid) and non-treated (dashed) *in silico* patients, same treatment strategies as Figure 4, in the space of $\varepsilon_i - \varepsilon_a - [nCoV]$ space.
