# Supplementary material for: Dynamical modelling of viral infection and cooperative immune protection in COVID-19 patients
Source: PLoS Comput Biol. 2023 Sep 1;19(9):e1011383. doi: 10.1371/journal.pcbi.1011383 (PMC10501599; doi:10.1371/journal.pcbi.1011383)
Supplement: S15 Fig — (PDF) [file pcbi.1011383.s016.pdf]

Figure S15

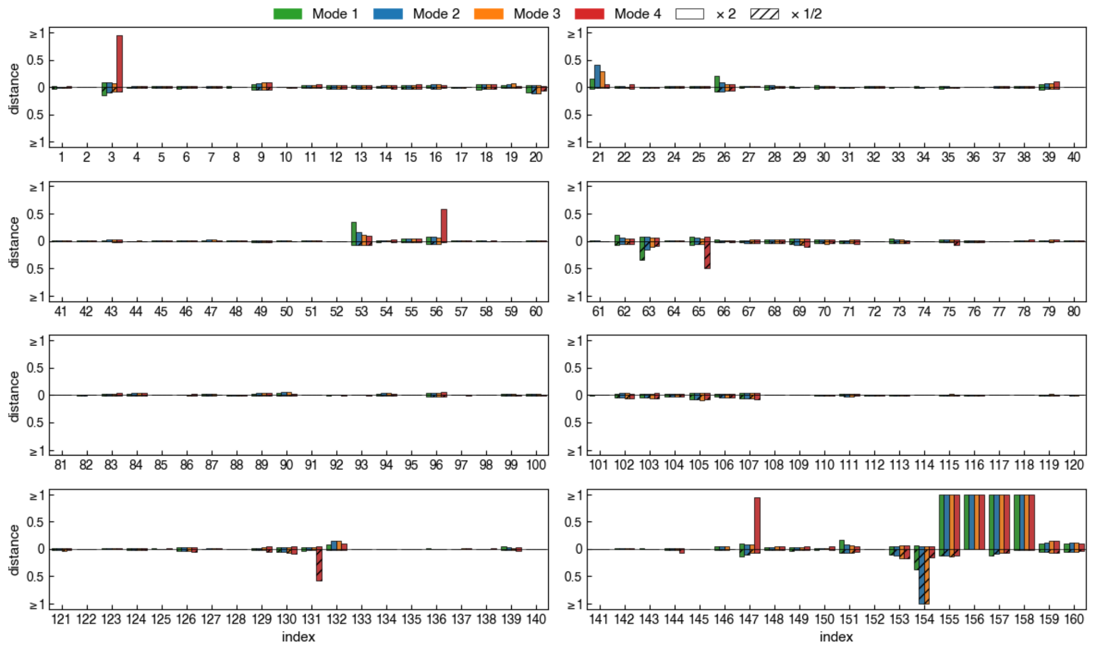

Figure S15. Parameter analysis on the model.

Each number on the x axis stands for the index of corresponding parameter in Table S2. Model's most sensitive parameters are t_{CD4}, t_{CD8}, $g_1 \sim g_4$, i.e., the generation of activated T cell expansion. Model's secondary sensitive parameters can be classified into several categories, including the virulence-related parameters, k_{infect}, r_H, d_H, and N_1; APC-activation-related parameters k_{rcr}^{APC}, $h_{IFN-\gamma}^{APC}$, d_{APC^l}, and APC_0, and humoral-immunity-related parameters r_{GC} and K_{AB}.
