# Supplementary material for: Dynamical modelling of viral infection and cooperative immune protection in COVID-19 patients
Source: PLoS Comput Biol. 2023 Sep 1;19(9):e1011383. doi: 10.1371/journal.pcbi.1011383 (PMC10501599; doi:10.1371/journal.pcbi.1011383)
Supplement: S16 Fig — (PDF) [file pcbi.1011383.s017.pdf]

Figure S16

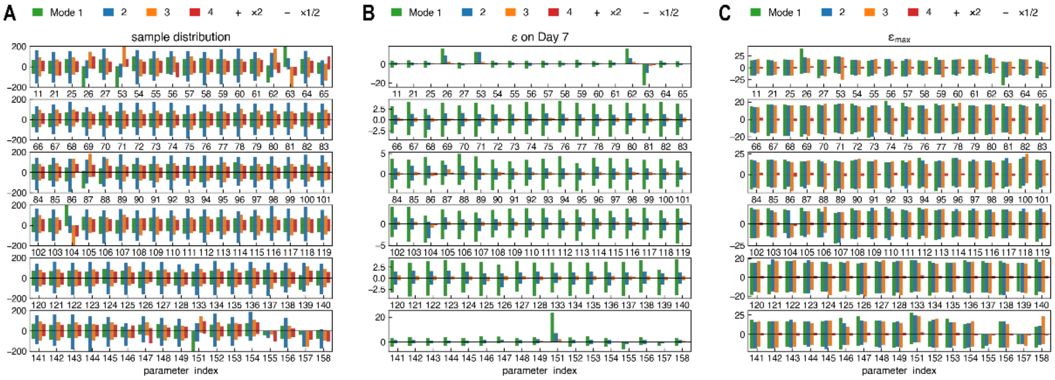

Figure S16. Robustness against fixed parameters.

Parameter indices can be referred to the parameter table Table S2.

(A) The change in the fixed parameter and thus parameter space shifted the distribution of Mode 1~4 patients yielded during sampling. Columns height refer to the number of samples for each mode yielded from the initial 1000 random samples. Columns above zero refer to the results where certain parameter is increased by 2-fold for sampling, and columns below zero refer to the results where the parameter is decreased by 2-fold (1/2).

(B) Change of early immune efficacy on day 7 post infection.

(C) Change of maximum immune efficacy. The maximum immune efficacy of mode 4 is significantly lower than γ. In particular, virulence-related parameters k_{infect}, r_H, d_H, and N_1, affect the virulence and therefore the entire viral dynamics. Loaded APC decay rated_{APC^l}, and IL-6 decay rate c_{IL-6}, decide the inflammatory level, therefore the distribution of Mode 1~4 are shifted accordingly. CD4+ and CD8+ T cell's proliferation generations, g_1 and g_3, determines the maximum T cell level, that they affect the course of immune response, and increase of these parameters will lead to T cell density to cross the upper limit of physiological range. As these results alter the distribution of mode 1~4 samples, and the magnitude of immune efficacy, our main results regarding the COVID pathogenesis remain robust. On day 7, the immune efficacy consistently decreases from Mode 1 to Mode 4, suggesting weak early immunity results in more severe inflammation, while the maximum immune efficacy needs to exceed

virulence γ to clear the infection.
