# Supplementary material for: Dynamical modelling of viral infection and cooperative immune protection in COVID-19 patients
Source: PLoS Comput Biol. 2023 Sep 1;19(9):e1011383. doi: 10.1371/journal.pcbi.1011383 (PMC10501599; doi:10.1371/journal.pcbi.1011383)
Supplement: S17 Fig — (PDF) [file pcbi.1011383.s018.pdf]

Figure S17

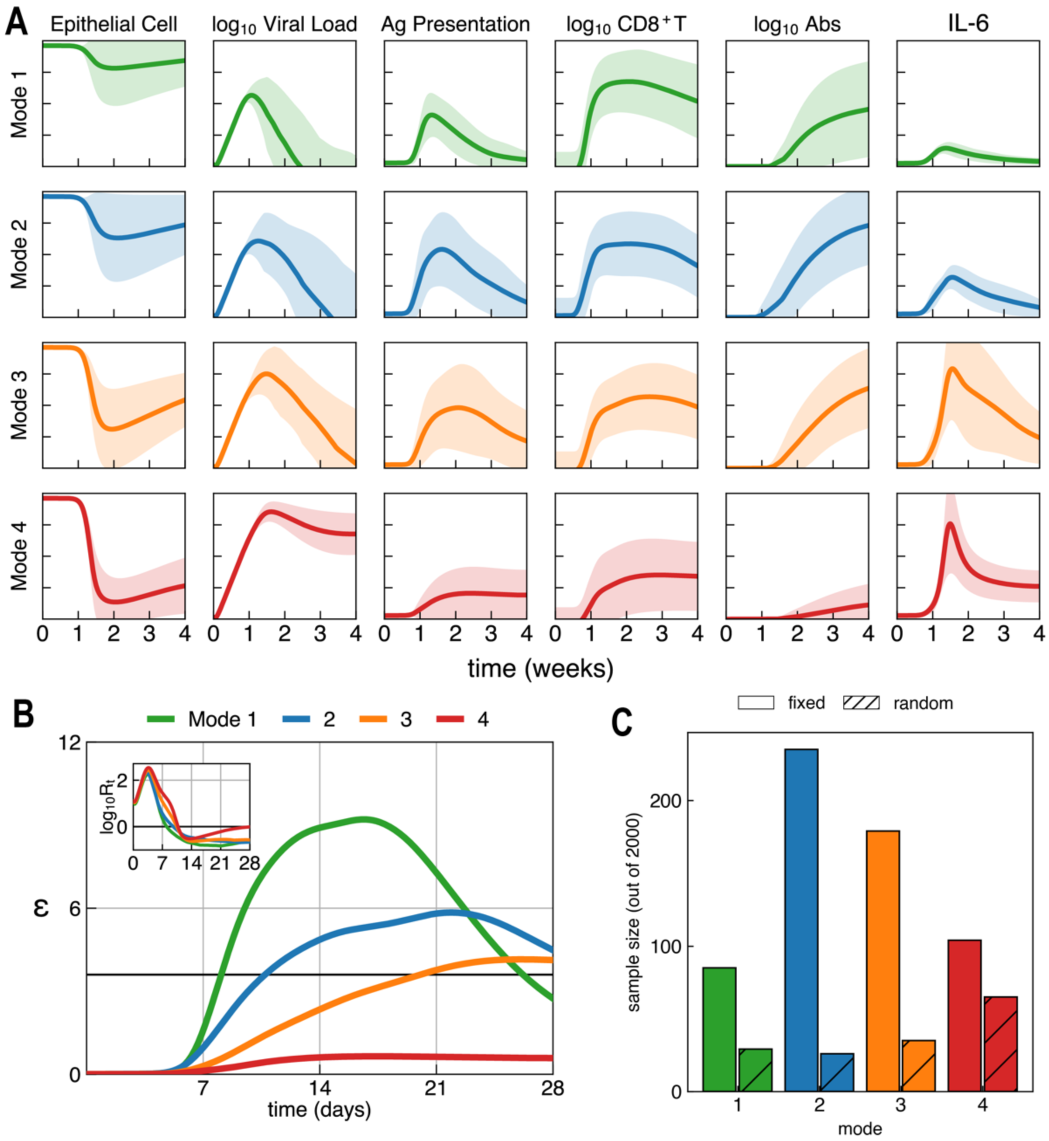

Figure S17. Robustness against random sampling.

Random sampling of the fixed parameters does not change the time course of viral and immune dynamics, and immune efficacy, but results in lower sampling efficiency and shift in mode distribution.

(A) Viral and immune kinetics.

(B) Time course of immune efficacy.

(C) Shift of the distribution of mode 1~4 samples when the previously fixed parameters are randomly sampled.
