# Supplementary material for: Dynamical modelling of viral infection and cooperative immune protection in COVID-19 patients
Source: PLoS Comput Biol. 2023 Sep 1;19(9):e1011383. doi: 10.1371/journal.pcbi.1011383 (PMC10501599; doi:10.1371/journal.pcbi.1011383)
Supplement: S18 Fig — (PDF) [file pcbi.1011383.s019.pdf]

Figure S18

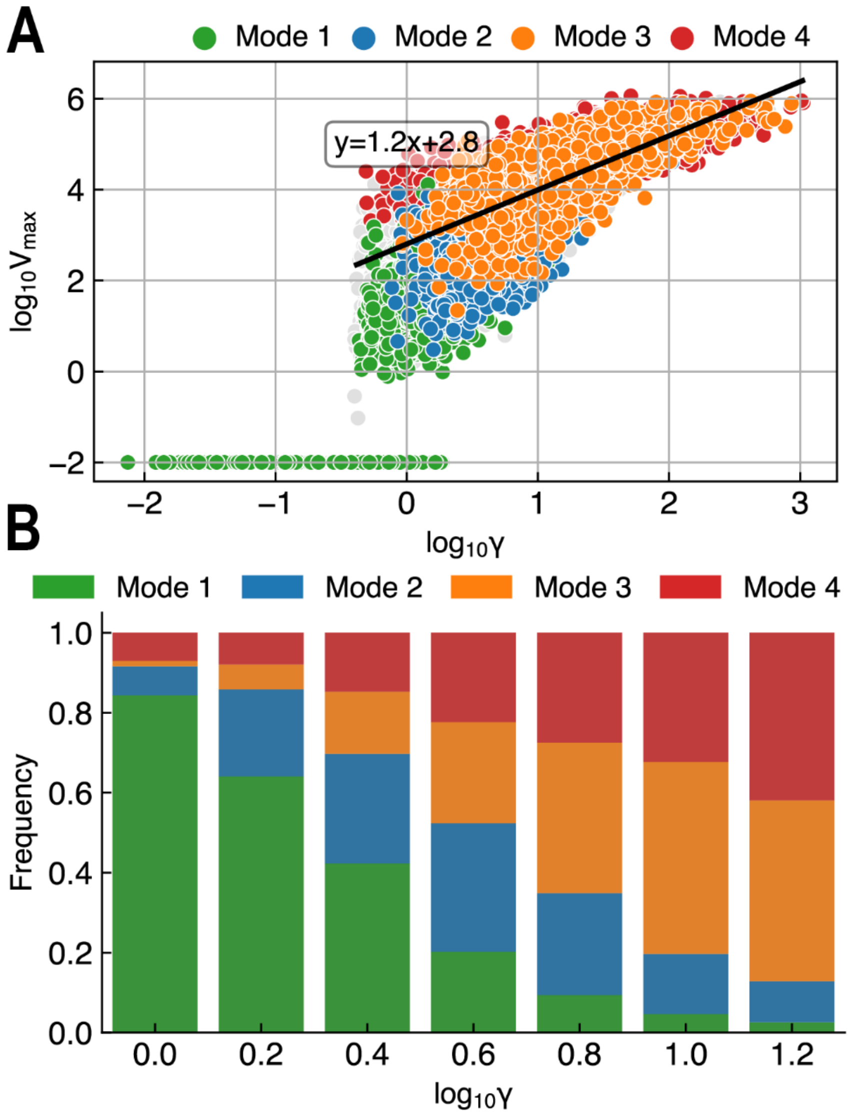

Figure S18. Virulence dictates maximum viral load during infection.

(A) By keeping parameter ranges the same as Figure 2, we loosen the constraints on virulence related parameters, and assume the four virulence-related parameters, viral infectivity k_{infect}, target cell abundance $[H]_0$, burst size N_1 and the dying rate of infected cells d_{If} to vary by 5-fold under logarithm, and sample again. Here we find as γ goes to infinity, the maximum viral load follows a 1.2-power law against γ.

(B) As virulence varies, the ratio of Mode 1~4 samples changes.
