# Supplementary material for: Dynamical modelling of viral infection and cooperative immune protection in COVID-19 patients
Source: PLoS Comput Biol. 2023 Sep 1;19(9):e1011383. doi: 10.1371/journal.pcbi.1011383 (PMC10501599; doi:10.1371/journal.pcbi.1011383)
Supplement: S19 Fig — (PDF) [file pcbi.1011383.s020.pdf]

Figure S19

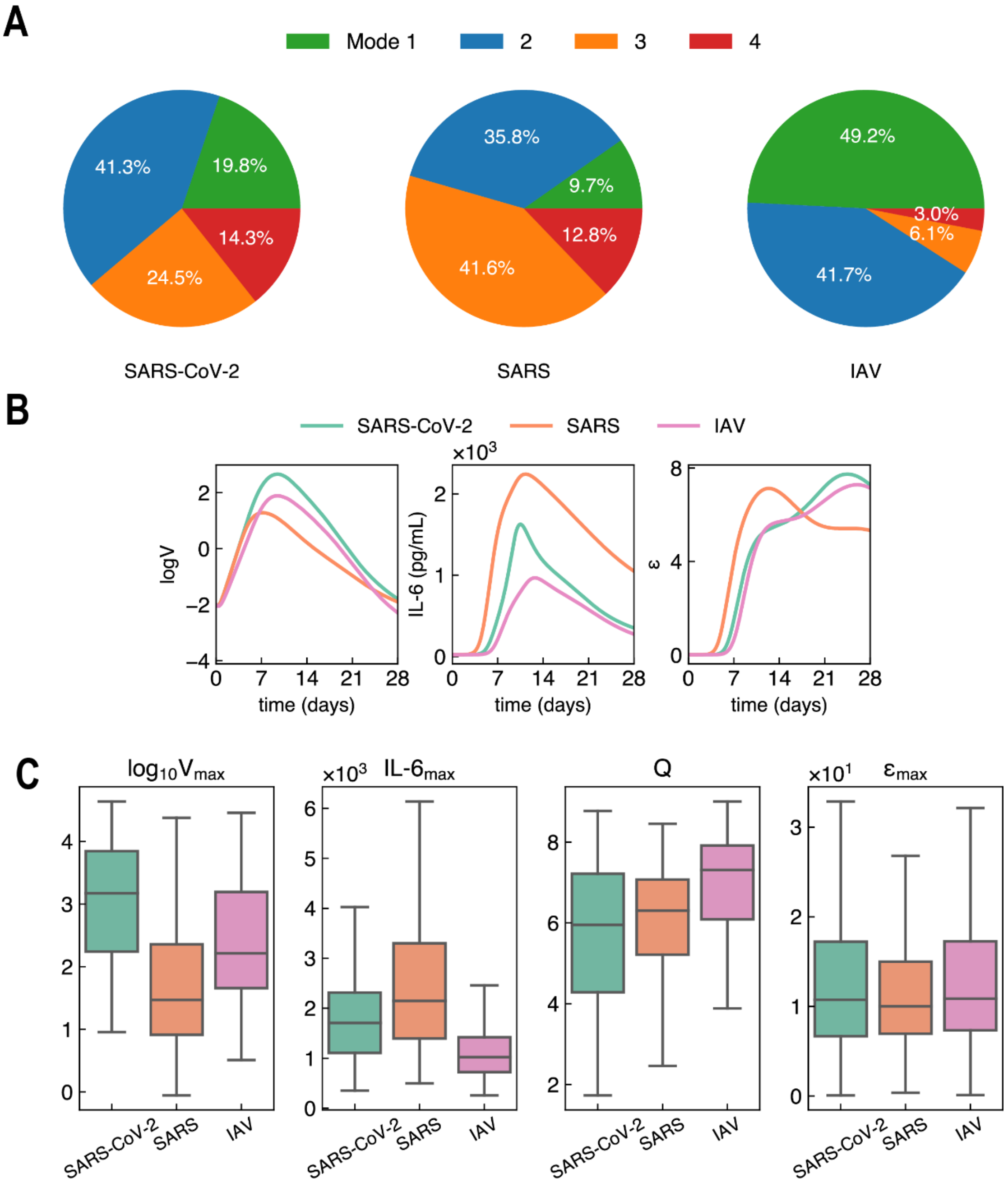

Figure S19. Infection dynamics of SARS and Influenza A Virus (IAV).

(A) Distribution of mode 1~4 during primary infection of SARS-CoV-2, SARS and IAV.

(B) Time course of virus and infection dynamics. In comparison to SARS-CoV-2, SARS infection and influenza infection induces stronger innate immune response (IFN-I). Due to the difference in virulence

and activation of adaptive immune response, the time courses of inflammation and immune efficacy are different.

(C) Sample distribution of infection characters in different viral infections.
