# Supplementary material for: Dynamical modelling of viral infection and cooperative immune protection in COVID-19 patients
Source: PLoS Comput Biol. 2023 Sep 1;19(9):e1011383. doi: 10.1371/journal.pcbi.1011383 (PMC10501599; doi:10.1371/journal.pcbi.1011383)
Supplement: S20 Fig — (PDF) [file pcbi.1011383.s021.pdf]

Figure S20

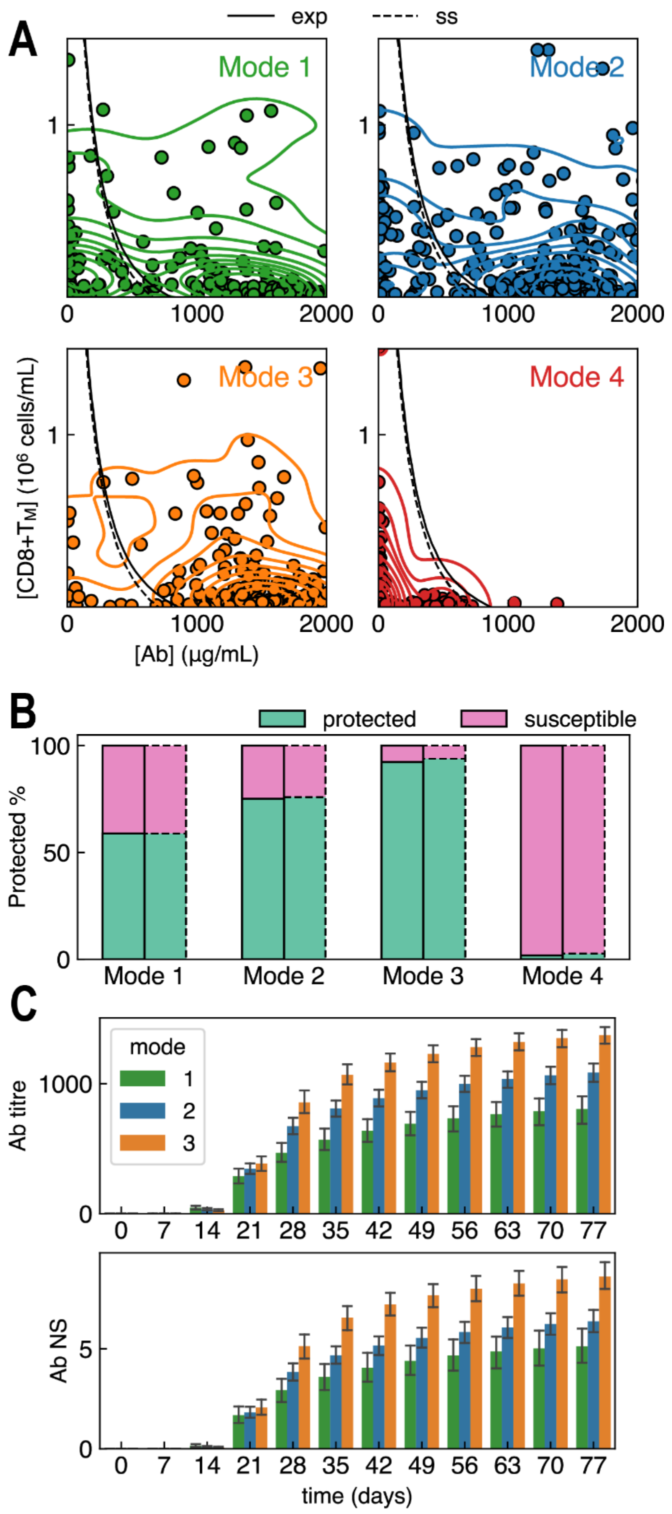

Figure S20. Immunogenicity data from model simulation of primary infection.

(A) Distribution of $CD8+T_M$ and B_M level 50 days after initial infection, black line denotes the average protection border of mode 1~4.

(B) Protection rate of convalescent patients in simulation.

(C) Ab formation during the infection process. Ab NS refers to the neutralizing strength of antibodies, defined as $k_4^{clear} Ab \cdot A$.
