# Supplementary material for: Dynamical modelling of viral infection and cooperative immune protection in COVID-19 patients
Source: PLoS Comput Biol. 2023 Sep 1;19(9):e1011383. doi: 10.1371/journal.pcbi.1011383 (PMC10501599; doi:10.1371/journal.pcbi.1011383)
Supplement: S21 Fig — (PDF) [file pcbi.1011383.s022.pdf]

# Figure S21

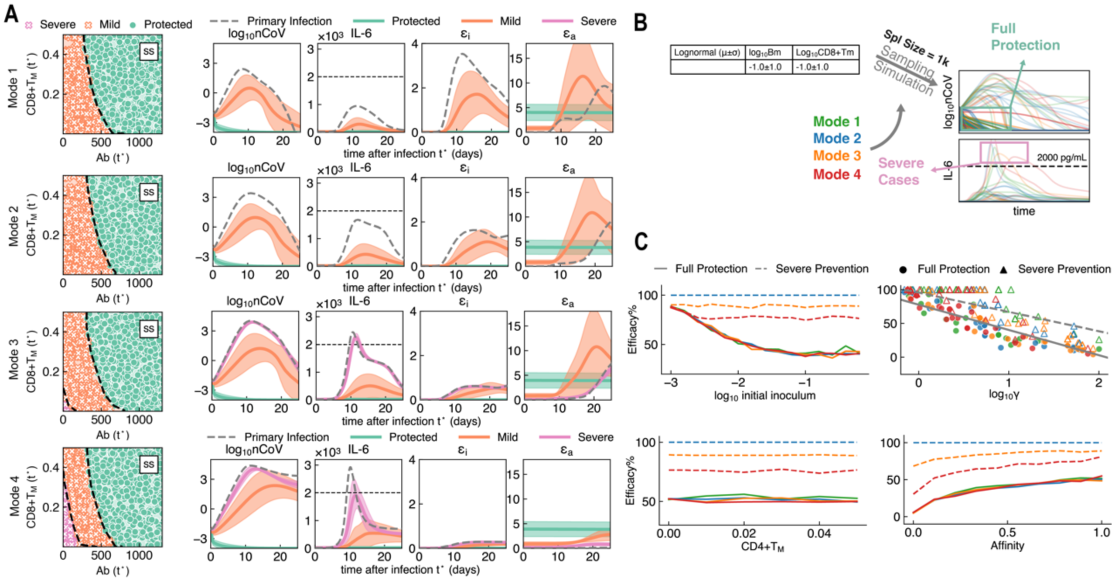

**Figure S21. Vaccine protection simulation.**

(A) Different outcomes of Mode 1~4 parameter sets (Table S4) with different immune memory level. Simulations are done using the limit case where Ab reaches the steady state determined by $B_M$ ('ss').

(B) Schematic of defining protection rate during simulation. Joint lognormal distribution of memory CD8+ T and B cells are assumed, matched with parameter sets drawn from the sample. Full protection is defined as maximum viral load=initial inoculum, and severe cases are defined as the ones with maximum IL-6 > 2000 pg/mL. Colors of the curves correspond to Mode 1~4.

(C) Different factors' impact on simulated (full and severe) protection rate. In particular, the dependence of protection rate on virulence $\gamma$ is inferred by sampling the virulence-related parameters, with each circle represents the full protection rate of one particular set of virulence-related parameters, and each triangle the severe protection rate.
