# Supplementary material for: Dynamical modelling of viral infection and cooperative immune protection in COVID-19 patients
Source: PLoS Comput Biol. 2023 Sep 1;19(9):e1011383. doi: 10.1371/journal.pcbi.1011383 (PMC10501599; doi:10.1371/journal.pcbi.1011383)
Supplement: S22 Fig — (PDF) [file pcbi.1011383.s023.pdf]

Figure S22

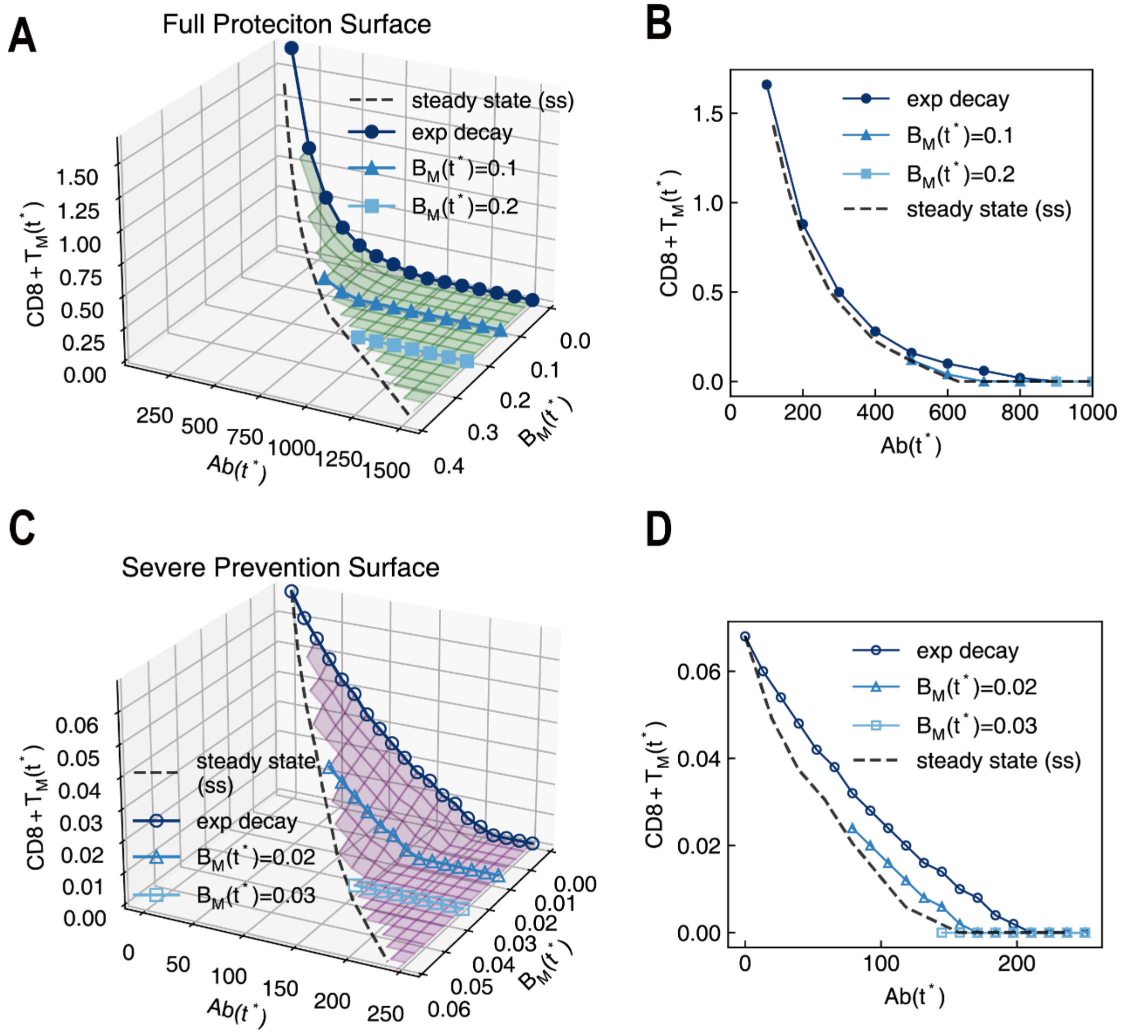

Figure S22. Full protection surface and severe prevention surface in initial Ab, B_M and CD8+T_M space.

(A) The host is fully protected (maximum viral load = initial inoculum), when the initial immune state (t=t*) is on the top of full protection surface (green). Normally, Ab exponentially declines to the steady state (‘intermediate’ case), which is constrained between two limit cases defined in the main text, ‘ss’ (dashed) and ‘exp’ (dark blue line with circles). Light blue lines show two protection borders in ‘intermediate’ cases with fixed initial B_M = 0.1 or 0.2.

(B) Four protection borders in (A) are projected on initial Ab and CD8+T_M plane. Initial levels of B_M hardly affect the protection border.

(C) The host is prevented from severe infection defined as maximum IL-6 level > 2000 pg/mL, when the initial immune state (t=t*) is on the top of severe prevention surface (purple). Two lighter blue lines show examples of severe prevention borders in ‘intermediate’ cases with fixed initial B_M = 0.02 or 0.03. Dashed line stands for prevention border in ‘ss’ limit case, as the border in ‘exp’ limit case is dark blue line with circles.

(D) Four severe prevention borders in (C) are projected on initial Ab and CD8+T_M plane. Increased initial B_M reduces severe prevention thresholds of Ab and CD8+T_M.

In all simulations of (A-D), we used the geometric mean parameter set of Mode 3 with initial [CD4+T_M] = 0.02.
