# Supplementary material for: Dynamical modelling of viral infection and cooperative immune protection in COVID-19 patients
Source: PLoS Comput Biol. 2023 Sep 1;19(9):e1011383. doi: 10.1371/journal.pcbi.1011383 (PMC10501599; doi:10.1371/journal.pcbi.1011383)
Supplement: S23 Fig — (PDF) [file pcbi.1011383.s024.pdf]

Figure S23

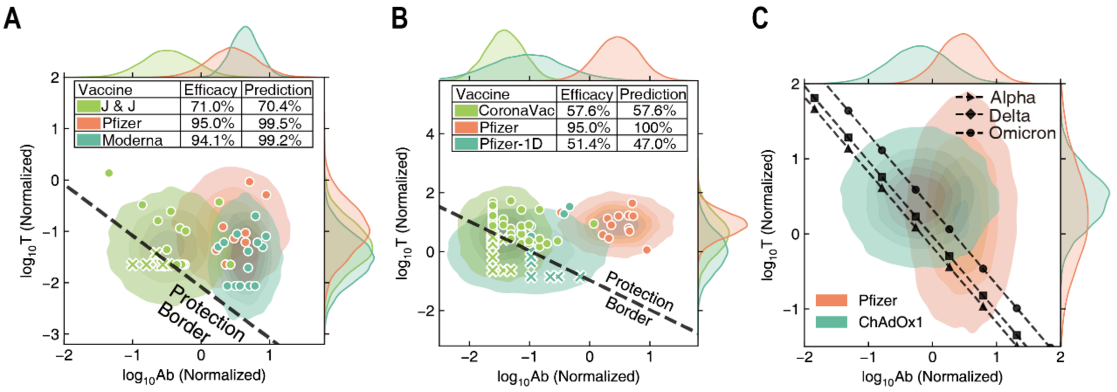

Figure S23. Immune memory distributions of different vaccines and predictions of efficacy against wildtype SARS-CoV-2 and variants.

(A) Fitted distributions of neutralizing antibody titers and T cell response (cytometry data) in Johnson & Johnson (J & J), Pfizer (BNT162b2) and Moderna (mRNA-1273).

(B) Fitted distributions of neutralizing antibody titers and T cell response (Elispot data) in two dose Pfizer, one dose Pfizer (Pfizer-1D) and CoronaVac. The source of Pfizer data is different from Fig3E, see details in Table S6. The black dashed lines in (A-B) is the protection border against wildtype SARS-CoV-2 infection.

(C) Fitted protection borders against SARS-CoV-2 variants and distributions of neutralizing antibody titers and T cell response (Elispot data) in Pfizer and ChAdOx1. Black lines indicate the protection border of Alpha (triangles), Delta (squares) and Omicron (circles) variants.
