# Supplementary material for: Dynamical modelling of viral infection and cooperative immune protection in COVID-19 patients
Source: PLoS Comput Biol. 2023 Sep 1;19(9):e1011383. doi: 10.1371/journal.pcbi.1011383 (PMC10501599; doi:10.1371/journal.pcbi.1011383)
Supplement: S24 Fig — (PDF) [file pcbi.1011383.s025.pdf]

Figure S24

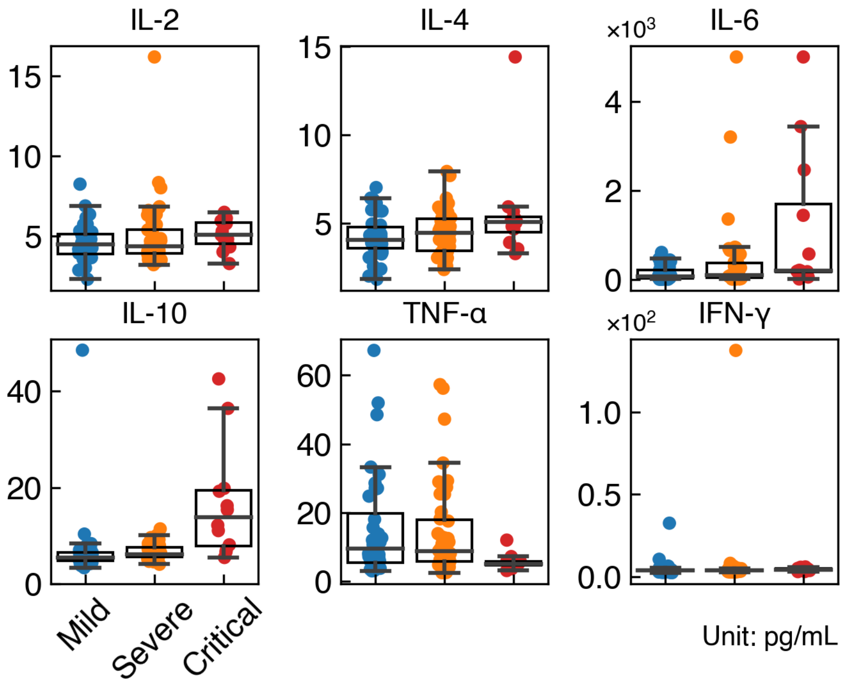

Figure S24. Physiological range of the cytokines in 95 clinical patients infected with WT SARS-CoV-2 virus.
