# Supplementary material for: Dynamical modelling of viral infection and cooperative immune protection in COVID-19 patients
Source: PLoS Comput Biol. 2023 Sep 1;19(9):e1011383. doi: 10.1371/journal.pcbi.1011383 (PMC10501599; doi:10.1371/journal.pcbi.1011383)
Supplement: S25 Fig — (PDF) [file pcbi.1011383.s026.pdf]

Figure S25

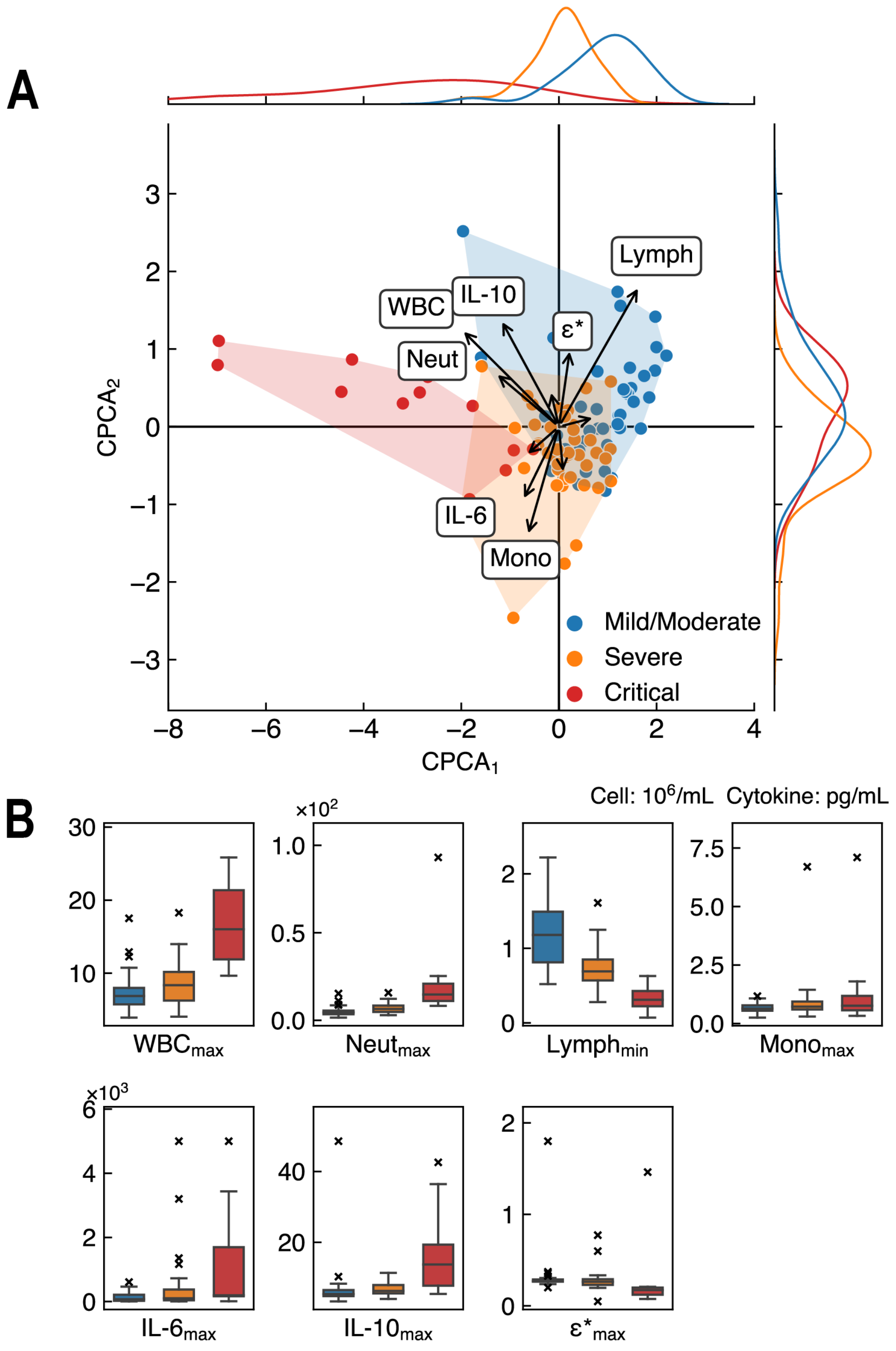

Figure S25. CPCA results on patients' peripheral blood data. White blood cell counts (WBC), neutrophil

counts (Neut), lymphocyte counts (Lymph), monocyte counts (Mono), IL-6 level, IL-10 level differs significantly among the mild/moderate, severe and critical patients.
