# Supplementary material for: Dynamical modelling of viral infection and cooperative immune protection in COVID-19 patients
Source: PLoS Comput Biol. 2023 Sep 1;19(9):e1011383. doi: 10.1371/journal.pcbi.1011383 (PMC10501599; doi:10.1371/journal.pcbi.1011383)
Supplement: S26 Fig — (PDF) [file pcbi.1011383.s027.pdf]

Figure S26

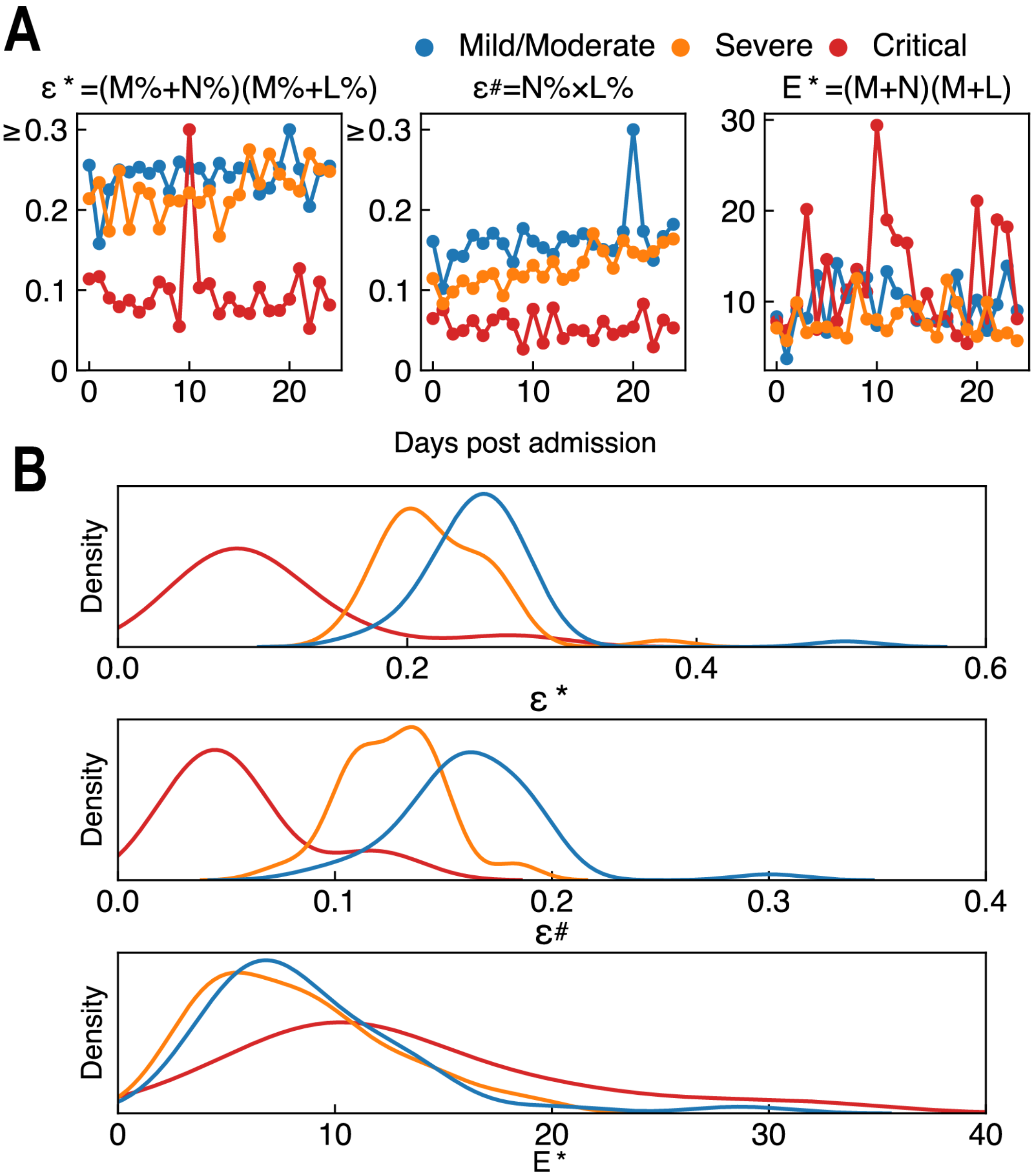

Figure S26. Different definition of clinical indicator for immune efficacy.

(A) Average time course of various clinical indicators for immune efficacy.

(B) Distribution of among altogether 95 patients (40 mild/moderate, 43 severe, 12 critical cases).
