# Supplementary material for: Dynamical modelling of viral infection and cooperative immune protection in COVID-19 patients
Source: PLoS Comput Biol. 2023 Sep 1;19(9):e1011383. doi: 10.1371/journal.pcbi.1011383 (PMC10501599; doi:10.1371/journal.pcbi.1011383)
Supplement: S27 Fig — (PDF) [file pcbi.1011383.s028.pdf]

# Figure S27

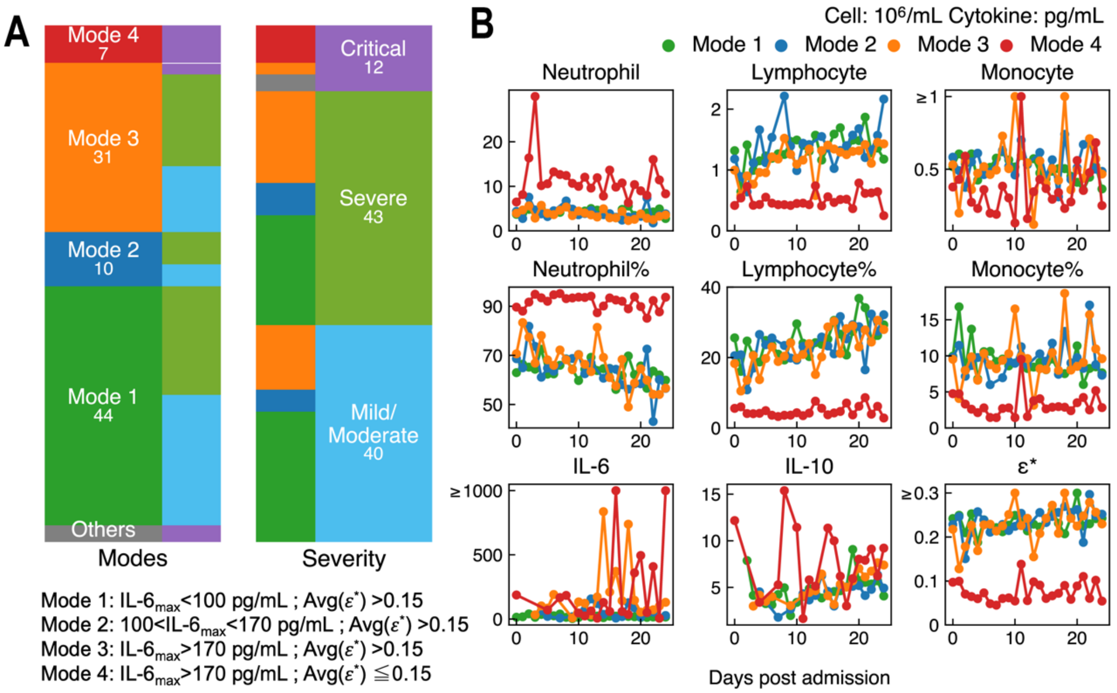


**Figure S27. A total of 95 clinical patients are classified by their IL-6 level and immune efficacy in peripheral blood.**

(A) We identify 44 patients as mode 1 (24 mild / moderate patients, 20 severe patients), 10 patients as mode 2 (4 mild/moderate patients, 6 severe patients), 31 patients as mode 3 (12 mild/moderate patients, 17 severe patients and 2 critical patients), and 7 patients as mode 4 profile (all critical). Three critical cases can't be classified based on definition, and are taken as Others.

(B) Averaged time series of mode 1, 2, 3, and 4 patients. Compared to the flat curve of mode 1 and 2 patients, mode 3 and 4 patients show different extent of inflammation, as characterized by IL-6 level. In addition, mode 4 patients show lower $\varepsilon^*$ level, compared to mode 1 and 2 patients.
