# Supplementary material for: Dynamical modelling of viral infection and cooperative immune protection in COVID-19 patients
Source: PLoS Comput Biol. 2023 Sep 1;19(9):e1011383. doi: 10.1371/journal.pcbi.1011383 (PMC10501599; doi:10.1371/journal.pcbi.1011383)
Supplement: S28 Fig — (PDF) [file pcbi.1011383.s029.pdf]

Figure S28

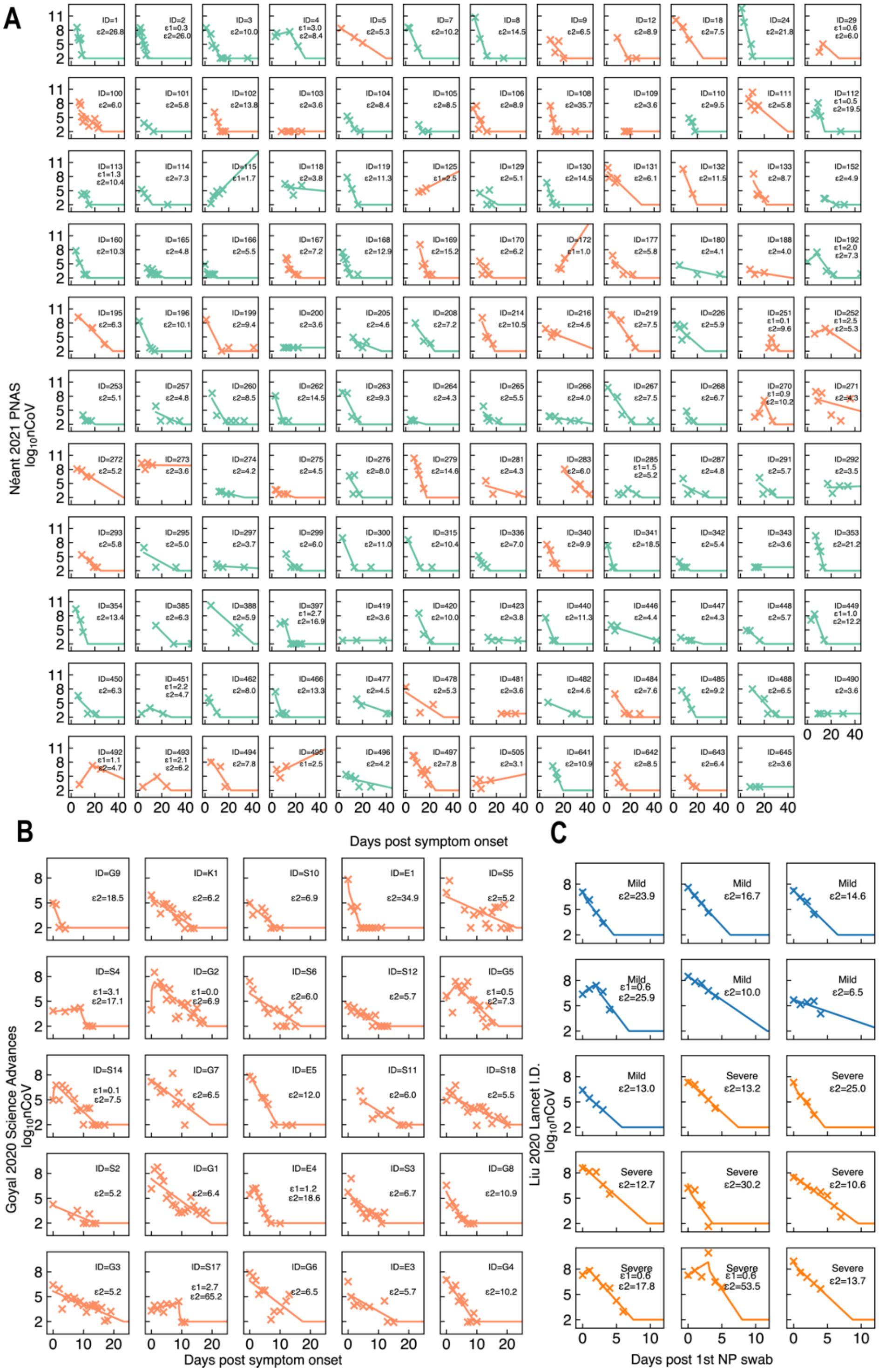

Figure S28. Fitting the viral dynamics data to the model (Eq. (4) ~ (5) in the main text) gives out a

maximum estimate for ε.

(A) Data from [48], green for age < 65 yrs old and orange for age ≥ 65 yrs old.

(B) Data from [49].

(C) Data from [50], Ct values are collected and transformed by $\log_{10} nCoV = 8 - \log_{10} 2 \times Ct$.
