# Supplementary material for: Dynamical modelling of viral infection and cooperative immune protection in COVID-19 patients
Source: PLoS Comput Biol. 2023 Sep 1;19(9):e1011383. doi: 10.1371/journal.pcbi.1011383 (PMC10501599; doi:10.1371/journal.pcbi.1011383)
Supplement: S29 Fig — (PDF) [file pcbi.1011383.s030.pdf]

Figure S29

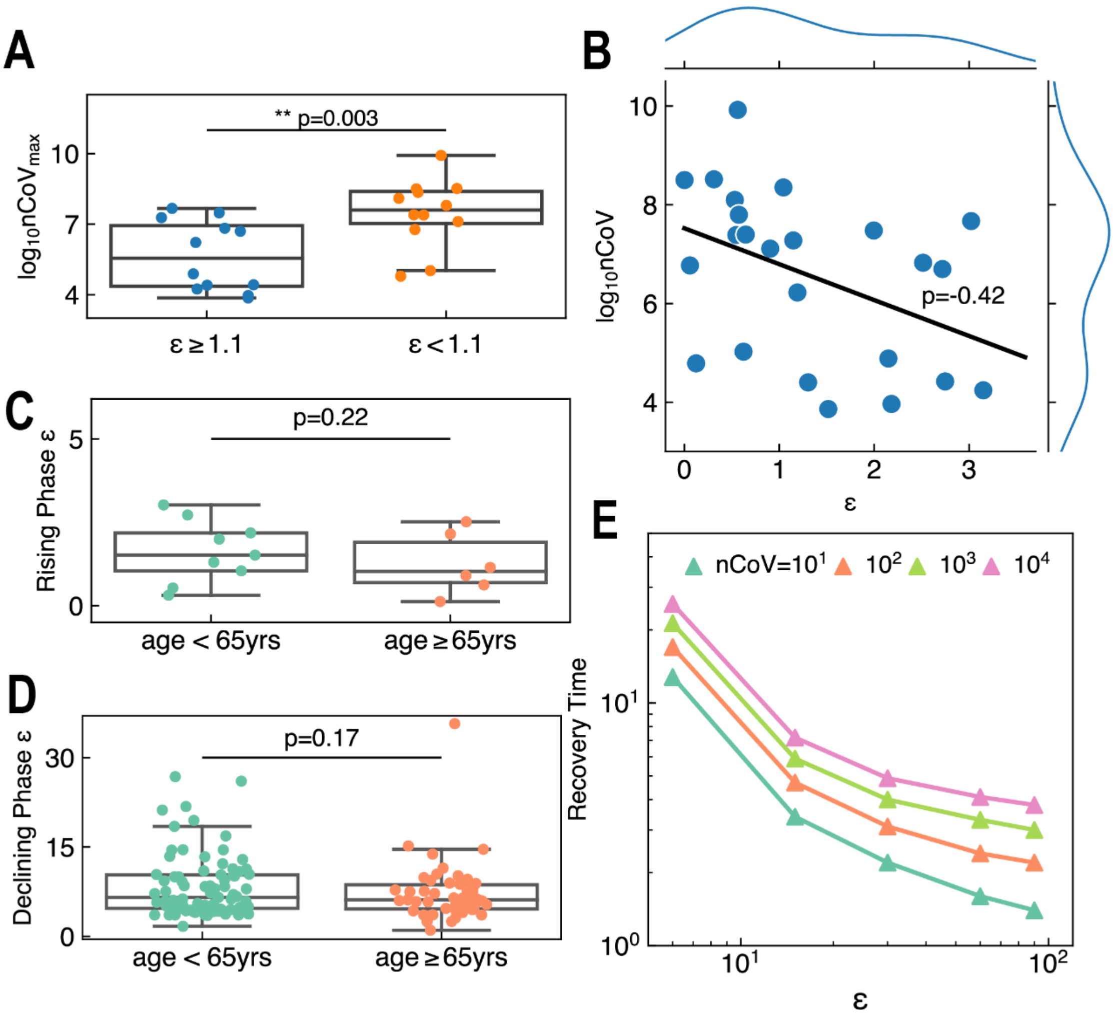

Figure S29. Statistics on the fitted immune efficacy.

(A) In the rising phase (time before viral load reaches peak), patients' ε value affects their maximum viral load (Whitney-Mann U Test, p=0.003).

(B) In the 24 individuals with rising phase observed, their maximum viral loads are negatively correlated with rising phase immune efficacy.

(C and D) During the rising and declining phase, patients with age < 65 yrs old show stronger immune efficacy ε than elderly patients (age ≥ 65 yrs old), but this difference is not significant (p=0.22 and 0.17,

respectively. Whitney-Mann U test). Data are collected from fitting viral dynamics data in (1).

(E) Simulation predicts the relation between declining phase ε and recovery time. The slope when ε is small $\approx$ -1.4, while when ε is large, ranges from -0.33 to -0.18.

Reference

1. Néant N, Lingas G, Le Hingrat Q, Ghosn J, Engelmann I, Lepiller Q, et al. Modeling SARS-CoV-2 viral kinetics and association with mortality in hospitalized patients from the French COVID cohort. Proceedings of the National Academy of Sciences. 2021;118(8):e2017962118.
