# Supplementary material for: Dynamical modelling of viral infection and cooperative immune protection in COVID-19 patients
Source: PLoS Comput Biol. 2023 Sep 1;19(9):e1011383. doi: 10.1371/journal.pcbi.1011383 (PMC10501599; doi:10.1371/journal.pcbi.1011383)
Supplement: S1 Table — (PDF) [file pcbi.1011383.s031.pdf]

Table S1.

Table S1. Estimated cell density in lung area and draining lymph nodes.

	Healthy Control		Severe & Critical	Baseline	Simulation
	Lower Limit	Upper Limit	Upper Limit		Upper Limit
BALF Cell Density (cells/mL)	1.17E+04	2.10E+04	2.25E+06		
BALF Cell Count (cells)	2.34E+05	4.20E+05	4.50E+07		
Alveolar Macrophage Proportion	0.52	0.98	0.86		
Alveolar Macrophage Density in Pulmonary Tissue (cells/mL)	1.20E+04	4.07E+04	**3.83E+06**	APC^u BaseLine: 2.00E+04	APC Upper Limit: 5.00E+06
Natural Killer Cell Proportion	0	0.023	0.04		
Natural Killer Cell Density in Pulmonary Tissue (cells/mL)	0.00E+00	9.55E+02	**1.78E+05**	0	3.00E+05
Neutrophil Proportion	0	0	0.26		

Neutrophil Density in Pulmonary Tissue (cells/mL)	0.00E+00	0.00E+00	**1.16E+06**	0	3.00E+06
Dendritic Cell Proportion	0.013	0.043	0.023		
Dendritic Cell Density in Pulmonary Tissue (cells/mL)	3.01E+02	1.79E+03	**1.02E+05**		
T Cell Proportion	0	0.406	0.339		
T Cell Density in Pulmonary Tissue (cells/mL)	0.00E+00	1.69E+04	**1.51E+06**	2.00E+04	8.00E+06
B Cell Proportion	0	0.017	0.327		
B Cell Density in Pulmonary Tissue (cells/mL)	0.00E+00	7.06E+02	**1.45E+06**	0	5.00E+06
