# Supplementary material for: Dynamical modelling of viral infection and cooperative immune protection in COVID-19 patients
Source: PLoS Comput Biol. 2023 Sep 1;19(9):e1011383. doi: 10.1371/journal.pcbi.1011383 (PMC10501599; doi:10.1371/journal.pcbi.1011383)
Supplement: S2 Table — (PDF) [file pcbi.1011383.s032.pdf]

Table S2.

Table S2. Choice of parameter and sampling range.

The parameter sets are generated by the sampling method in section 3.1 of SM in the parameter range above.

The parameter table is divided into six categories, viral infection, cellular interactions,

Viral Infection

index	parameter	description	value	unit	reference
26	k_{infect}	SARS-CoV-2 infection rate of target lung epithelial cell	0.00012	10^{-6}mL·day^{-1}	Influenza: 0.00003(1)
62	d_{If}	Infected cell dying rate due to viral infection	0.4	day^{-1}	1.5(1); 0.1(2); $d_{If}N_1 = 400$(3)
152	N_1	SARS-CoV-2 burst size	1500	1	~1000(4)

Cellular interactions

index	parameter	description	value	unit	reference
1	k_{nCoV}^{APC}	APC activation rate by SARS-CoV-2 antigen.	[0.02, 0.5]	day^{-1}	
2	k_{If}^{APC}	APC activation rate by infected cells	[0.02, 0.5]	day^{-1}	
3	k_{rcr}^{APC}	APC secreting chemokines to recruit APCs to site	[0.012, 0.3]	day^{-1}	

4	k_{If}^{NK}	NK activation by infected cell.	[0.001, 0.025]	day^{-1}	
5	k_{APC}^{NK}	NK activation by macrophage-derived cytokines.	[0.001, 0.025]	day^{-1}	
6	k_{If}^{Neut}	Neutrophil recruitment by infected cell-secreted chemokines.	[0.02, 0.5]	day^{-1}	
7	k_{D}^{Neut}	Neutrophil recruitment by DAMPs secreted by damaged cells	[0.02, 0.5]	day^{-1}	
8	k_{Th17}^{Neut}	Th17 recruitment of neutrophil through CXCL8 and activation via GM-CSF.	[0.01, 0.25]	day^{-1}	
9	k_{naive}^{CD4}	Naïve CD4+ T cell activation by APC	[0.015, 0.06]	day^{-1}	
10	k_{mem}^{CD4}	Memory CD4+ T cell activation by APC	[0.03, 0.12]	day^{-1}	
11	k_{CD4}^{mem}	Activated CD4+ T cell differentiating into CD4+ memory T cell	0.02	day^{-1}	0.01(5)

12	k_{CD4}^{Th1}	Activated CD4+ T cell differentiating into Th1	[0.2, 0.8]	day^{-1}	
13	k_{CD4}^{Th2}	Activated CD4+ T cell differentiating into Th2	[0.05, 0.2]	day^{-1}	
14	k_{CD4}^{Th17}	Activated CD4+ T cell differentiating into Th17	[0.05, 0.2]	day^{-1}	
15	k_{CD4}^{Tfh}	Activated CD4+ T cell differentiating into Tfh	[0.1, 0.4]	day^{-1}	
16	k_{CD4}^{iTreg}	Activated CD4+ T cell differentiating into Treg	[0.05, 0.2]	day^{-1}	
17	k_{APC}^{nTreg}	Resting Treg cell activation by APC	[0.06, 1.5]	day^{-1}	
18	k_{naive}^{CD8}	Naïve CD8+ T cell activation by APC	[0.02, 0.08]	day^{-1}	
19	k_{mem}^{CD8}	Memory CD8+ T cell activation by APC	[0.03, 0.12]	day^{-1}	
20	k_{CD8}^{CTL}	Activated CD8+ T cell differentiating into CTL	[0.2, 0.8]	day^{-1}	
21	k_{CD8}^{mem}	Activated CD8+ T cell differentiating into CD8+ memory T	0.04	day^{-1}	0.01(6); 0.02(5)

		cell			
22	k_{naive}^{GC}	Naïve B cell activation and entering germinal center	[0.15, 0.6]	day^{-1}	
23	k_{mem}^{GC}	Memory B cell activation and entering germinal center	[0.25, 1]	day^{-1}	
24	k_{PB}	Germinal center B cell differentiation into plasma cell	[0.025, 0.1]	day^{-1}	
25	k_{Bm}	Germinal center B cell differentiation into memory B cell	0.02	day^{-1}	

Antigen Presentation Hill constants

index	parameter	description	value	unit	reference
129	K_{ACD4}	Hill constant for antigen-presentation to naïve CD4+ T cell	[0.04, 1]	10^6/mL	
130	K_{ACD8}	Hill constant for antigen-presentation to naïve CD8+ T cell	[0.04, 1]	10^6/mL	
131	K_{AB}	Hill constant for antigen-presentation to naïve B cell	[0.1, 2.5]	10^6/mL	

132	K_{mem}	Hill constant for the formation of memory T cells at low antigen-level	[0.04, 1]	10^6/mL	

Cellular interaction Hill constants

index	parameter	description	value	unit	reference
134	K_1^{If}	Hill constant for APC activation by infected cell	0.1	10^6/mL	
135	K_2^{If}	Hill constant for NK activation by infected cell	0.1	10^6/mL	
136	K_3^{If}	Hill constant for neutrophil recruitment by infected cell	0.1	10^6/mL	
137	K_1^D	Hill constant for APC activation by damaged-cell-produced DAMP	20	10^6/mL	
138	K_2^D	Hill constant for neutrophil recruitment by damaged-cell-produced DAMP	20	10^6/mL	

139	K_1^{APC}	Hill constant for APC self-recruitment	0.2	10^6/mL	
140	K_2^{APC}	Hill constant for NK activation by APC	0.2	10^6/mL	
141	K_1^{Neut}	Hill constant for Th17 differentiation by neutrophil-secreted IL-1β	0.6	10^6/mL	
142	K_1^{Th1}	Hill constant for CTL differentiation by Th1	0.1	10^6/mL	
143	K_1^{Th17}	Hill constant for neutrophil activation and recruitment by Th17	0.05	10^6/mL	
144	K_1^{Tfh}	Hill constant for germinal center formation mediated by Tfh	0.02	10^6/mL	
145	K_1^{GC}	Hill constant for germinal center B cells to promote Tfh differentiation	0.5	10^6/mL	

DAMP and cytokine’s effect on immune cells during cellular interactions

index	parameter	description	value	unit	reference
37	h_D^{APC}	DAMP signal to activate the	[2, 8]	1	

		inflammatory and phagocytotic function of APC			
38	$h_{TNF-\alpha}^{APC}$	Activation of APC by TNF-α.	[1, 4]	1	
39	$h_{IFN-\gamma}^{APC}$	Activation of APC with increased antigen-presentation capacity by IFN-γ	[1, 4]	1	2.28(7)
40	h_{IL-2}^{NK}	Activation of NK efficacy by IL-2	[0.25, 1]	1	
41	h_{IL-2}^{CD4}	Activated CD4+ T cell proliferation promoted by IL-2	[0.25, 1]	1	
42	$h_{IFN-\gamma}^{Th1}$	Differentiation of naïve CD4+ T cell into Th1 by the help of IFN-γ.	[1, 4]	1	
43	h_{IL-4}^{Th2}	Differentiation of naïve CD4+ T cell into Th2 by the help of IL-4.	[1, 4]	1	
44	h_{IL-6}^{Th17}	Differentiation of naïve CD4+ T cell into Th17 by the help of IL-6 and TGF-β.	[0.5, 2]	1	
45	h_{Neut}^{Th17}	Differentiation of	[0.25, 1]	1	

		naïve CD4+ T cell into Th17 by the help of neutrophil-secreted IL-1β.			
46	h_{B}^{Tfh}	B cell activation of Tfh cell	[1, 4]	1	
47	h_{IL-10}^{iTreg}	Differentiation of naïve CD4+ T cell into Treg by the help of IL-10.	[1, 4]	1	
48	h_{IL-2}^{CD8}	IL-2 promotion of activated CD8+ T cell proliferation	[0.25, 1]	1	
49	h_{Th1}^{CTL}	Differentiation of naïve CD8+ T cell into CTL by the help of Th1.	[1.5, 6]	1	
50	h_{IL-2}^{CTL}	Differentiation of naïve CD8+ T cell into CTL by the help of IL-2.	[0.5, 2]	1	
51	h_{IL-6}^{CTL}	Differentiation of naïve CD8+ T cell into CTL by the help of IL-6.	[0.5, 2]	1	
52	h_{IL-4}^{Ab}	Increase of antibody secretion by the help	[0.1, 0.4]	1	

		of IL-4.			

Mucosal immune's clearance of virus

index	parameter	description	value	unit	reference
27	d_v	Mucosal immunity excluding virion particles	0.008	10^6/mL·day^{-1}	
133	K_m	Hill constant of mucosal immunity to clear the virus	0.001	10^6/mL	

Viral clearance by immune cells and Ab

index	parameter	description	value	unit	reference
28	k_1^{clear}	Antigen-loaded APC clearance of SARS-CoV-2 virion particles by phagocytosis.	[0.08, 2]	10^{-6}mL·day^{-1}	0.768(2)
29	k_2^{clear}	Unloaded APC clearance of SARS-CoV-2 virion particles by phagocytosis.	[0.08, 2]	10^{-6}mL·day^{-1}	
30	k_3^{clear}	Neutrophil clearance of SARS-CoV-2 virion particles by	[0.1, 2.5]	10^{-6}mL·day^{-1}	

		NET.			
31	k_4^{clear}	Antibody clearance of SARS-CoV-2 virion particles.	[0.003, 0.012]	$mL \cdot \mu g^{-1} \cdot day^{-1}$	

Infected cell elimination

index	parameter	description	value	unit	reference
32	k_1^{kill}	Antigen-loaded APC killing rate of SARS-CoV-2 infected cell.	[0.08, 2]	$10^{-6} mL \cdot day^{-1}$	0.121 (2)
33	k_2^{kill}	Unloaded APC killing rate of infected cell.	[0.08, 2]	$10^{-6} mL \cdot day^{-1}$	
34	k_3^{kill}	NK killing rate of SARS-CoV-2 infected cell.	[0.4, 10]	$10^{-6} mL \cdot day^{-1}$	
35	k_4^{kill}	CTL killing rate of SARS-CoV-2 infected cell.	[0.75, 3]	$10^{-6} mL \cdot day^{-1}$	1.1 (1)
36	k_5^{kill}	Memory CD8+ T cell killing rate of SARS-CoV-2 infected cell.	[0.5, 2]	$10^{-6} mL \cdot day^{-1}$	

Cell homeostasis proliferation/recruitment

index	parameter	description	value	unit	reference
53	r_H	Healthy epithelial cell regeneration rate	2	$10^6 ml^{-1} day^{-1}$	0.7~2.3 (1)

54	r_{APC}	APC renewal rate	0.004	10^6ml^{-1}day^{-1}	Initial state APC^u=2×10^4cells/mL
55	r_{Treg^r}	Resting Treg renewal rate	0.001	10^6ml^{-1}day^{-1}	Initial state Treg=2×10^4cells/mL

Treg inhibition of activated immune cells

index	parameter	description	value	unit	reference
58	d_{Treg}^{APC}	Treg inhibition of APC	0.4	10^{-6}mL·day^{-1}	
59	d_{Treg}^{NK}	Treg inhibition of NK	0.4	10^{-6}mL·day^{-1}	
60	d_{Treg}^{CD4}	Treg inhibition of activated CD4+ T cell	0.4	10^{-6}mL·day^{-1}	0.2 ~ 0.46 day^{-1} (5)
61	d_{Treg}^{CD8}	Treg inhibition of activated CD8+ T cell	0.4	10^{-6}mL·day^{-1}	

Activated cells inhibition / degradation / apoptosis rates

index	parameter	description	value	unit	reference
63	d_H	Healthy epithelial apoptosis rate	0.04	day^{-1}	
64	d_D	Damaged-cell-produced DAMP decay rate	0.05	day^{-1}	
65	d_{APC^l}	Antigen-loaded APC deactivation and	0.4	day^{-1}	0.01~0.9 (7);0.3 (2)

		apoptosis rate			
66	d_{APC^u}	Unloaded APC decay rate in lung	0.2	day^{-1}	
67	d_{NK}	NK deactivation and apoptosis rate	0.6	day^{-1}	
68	d_{Neut}	Neutrophil dying rate	1.6	day^{-1}	0.8~1.2 (8)
69	d_{Th}	Helper T cell apoptosis rate	0.8	day^{-1}	0.1~0.7 (7)
70	d_{Treg^a}	Activated Treg apoptosis rate	0.4	day^{-1}	
71	d_{Treg^r}	Resting Treg decay rate in lung	0.05	day^{-1}	
72	d_{CD4Tm}	CD4+ memory T cell net death rate	0.002	day^{-1}	0.0014(9)
73	d_{CTL}	CTL death rate	0.8	day^{-1}	0.1~0.7 (7); 0.4 (2)
74	d_{CD8Tm}	CD8+ memory T cell net death rate	0.0015	day^{-1}	0 for Murine CD8+ T Memory(5) 0.005 for SARS-CoV-2-specific CD8+ T Memory(10)
75	d_{GC}	Germinal center decay rate	0.2	day^{-1}	
76	d_{PB}	Plasma B cell death rate	0.4	day^{-1}	

77	d_{Bm}	Memory B cell net death rate	0.0003	day^{-1}	

Cytokine production

index	parameter	description	value	unit	reference
78	p_0^{IL-2}	IL-2 basic production rate	30	$pg \cdot mL^{-1} day^{-1}$	
79	p_1^{IL-2}	IL-2 production rate by Activated CD4+ T cell	80	$10^{-6} pg \cdot day^{-1}$	
80	p_2^{IL-2}	IL-2 production rate by Activated CD8+ T cell	80	$10^{-6} pg \cdot day^{-1}$	
81	p_3^{IL-2}	IL-2 production rate by Th1	60	$10^{-6} pg \cdot day^{-1}$	
82	p_4^{IL-2}	IL-2 production rate by CTL	40	$10^{-6} pg \cdot day^{-1}$	
83	p_0^{IL-4}	IL-4 basic production rate	100	$pg \cdot mL^{-1} day^{-1}$	
84	p_1^{IL-4}	IL-4 production rate by Th2	2000	$10^{-6} pg \cdot day^{-1}$	
85	p_0^{IL-6}	IL-6 basic production rate	200	$pg \cdot mL^{-1} day^{-1}$	
86	p_1^{IL-6}	IL-6 production rate by infected cell	1000	$10^{-6} pg \cdot day^{-1}$	
87	p_2^{IL-6}	IL-6 production rate	1500	$10^{-6} pg \cdot day^{-1}$	

		by APC			
88	p_3^{IL-6}	IL-6 production rate by neutrophil	3000	10^{-6}pg·day^{-1}	
89	p_0^{IL-10}	IL-10/TGF-β basic production rate	200	pg·mL^{-1}day^{-1}	
90	p_1^{IL-10}	IL-10/TGF-β production rate by activated Treg	4000	10^{-6}pg·day^{-1}	
91	p_2^{IL-10}	IL-10/TGF-β production rate by resting Treg	1000	10^{-6}pg·day^{-1}	
92	$p_0^{TNF-\alpha}$	TNF-α basic production rate	200	pg·mL^{-1}day^{-1}	
93	$p_1^{TNF-\alpha}$	TNF-α production rate by infected cell	40	10^{-6}pg·day^{-1}	
94	$p_2^{TNF-\alpha}$	TNF-α production rate by APC	400	10^{-6}pg·day^{-1}	
95	$p_3^{TNF-\alpha}$	TNF-α production rate by NK	200	10^{-6}pg·day^{-1}	
96	$p_0^{IFN-\gamma}$	IFN-γ basic production rate	100	pg·mL^{-1}day^{-1}	
97	$p_1^{IFN-\gamma}$	IFN-γ production rate by NK	200	10^{-6}pg·day^{-1}	
98	$p_2^{IFN-\gamma}$	IFN-γ production rate by Th1	100	10^{-6}pg·day^{-1}	
99	$p_3^{IFN-\gamma}$	IFN-γ production rate by CTL	100	10^{-6}pg·day^{-1}	

100	p_1^{Ab}	Antibody production rate by plasma cell	250	10^{-6}μg·day^{-1}	50~340 (11)
101	p_2^{Ab}	Antibody production rate by memory B cell	150	10^{-6}μg·day^{-1}	50~340 (11)

Cytokine degradation

index	parameter	description	value	unit	reference
102	c_{IL-2}	IL-2 decay rate	10	day^{-1}	
103	c_{IL-4}	IL-4 decay rate	10	day^{-1}	
104	c_{IL-6}	IL-6 decay rate	10	day^{-1}	16.6 (2)
105	c_{IL-10}	IL-10/TGF-β decay rate	10	day^{-1}	1.8~12 (7)
106	$c_{TNF-\alpha}$	TNF-α decay rate	10	day^{-1}	25~720 (7)
107	$c_{IFN-\gamma}$	IFN-γ decay rate	10	day^{-1}	1~80 (7); 17 (2)
108	c_{Ab}	Antibody decay rate	0.04	day^{-1}	0.8/month (12)<br>0.043/day (1)

Cytokine Hill constants

index	parameter	description	value	unit	reference
109	K_1^{IL-2}	Hill constant for IL-2 binding to NK	80	pg/mL	
110	K_2^{IL-2}	Hill constant for IL-2 promoting the proliferation of activated CD4+ T cell	80	pg/mL	
111	K_3^{IL-2}	Hill constant for IL-2 promotion of iTreg	40	pg/mL	

		differentiation			
112	K_4^{IL-2}	Hill constant for IL-2 promotion of nTreg activation	40	pg/mL	
113	K_5^{IL-2}	Hill constant for IL-2 promoting the proliferation of activated CD8+ T cell	80	pg/mL	
114	K_6^{IL-2}	Hill constant for IL-2 promotion of CTL differentiation	80	pg/mL	
115	K_1^{IL-4}	Hill constant for IL-4 binding to naïve CD4+ T cell for inhibition of Th1 differentiation	30	pg/mL	
116	K_2^{IL-4}	Hill constant for IL-4 binding to naïve CD4+ T cell for promotion of Th2 differentiation	30	pg/mL	
117	K_3^{IL-4}	Hill constant for IL-4 promoting the production of antibody	30	pg/mL	
118	K_1^{IL-6}	Hill constant for IL-6 promotion of Th17	1500	pg/mL	

		differentiation			
119	K_2^{IL-6}	Hill constant for IL-6 inhibition of iTreg differentiation	1500	pg/mL	
120	K_3^{IL-6}	Hill constant for IL-6 for CTL activation	1500	pg/mL	
121	K_1^{IL-10}	Hill constant for IL-10/TGF-β inhibition of Th differentiation	100	pg/mL	
122	K_2^{IL-10}	Hill constant for TGF-β promotion for Th17 differentiation	80	pg/mL	
123	K_3^{IL-10}	Hill constant for IL-10/TGF-β promotion for iTreg differentiation	80	pg/mL	
124	K_4^{IL-10}	Hill constant for IL-10/TGF-β inhibition of CTL differentiation	100	pg/mL	
125	$K_1^{TNF-\alpha}$	Hill constant for TNF-α binding to APC	100	pg/mL	
126	$K_1^{IFN-\gamma}$	Hill constant for IFN-γ binding to APC	80	pg/mL	45-1100 pg/mL(7)
127	$K_2^{IFN-\gamma}$	Hill constant for IFN-γ promotion for Th1	80	pg/mL	

		differentiation			
128	$K_3^{IFN-\gamma}$	Hill constant for IFN-γ inhibition of Th2 differentiation	80	pg/mL	

Immune cell reservoir

index	parameter	description	value	unit	reference
147	APC_0	APC reserve size	5	10^6/mL	
148	NK_0	NK reserve size	5	10^6/mL	
149	$Neut_0$	Neutrophil reserve size	10	10^6/mL	
150	B_0	B lymphocyte reserve size	[0.002, 0.05]	10^6/mL	
159	$CD4^+T_n$	Antigen-specific naïve CD4+ T cell frequency	[0.004, 0.1]	10^6/mL	
160	$CD8^+T_n$	Antigen-specific naïve CD8+ T cell frequency	[0.002, 0.05]	10^6/mL	

T cell activation and exhaustion

index	parameter	description	value	unit	reference
152	N_{ex}	Exhaustion of CTL by killing infected cell	10	1	10 (1)
153	t_{CD4}	Activated CD4+ T cell division time	0.417	day	10 hours(13)

154	t_{CD8}	Activated CD8+ T cell division time	0.25	day	6 hours(13)
155	g_1	Activated naïve CD4+ T cell division generations	11		9(13)
156	g_2	Activated memory CD4+ T cell division generations	6		
157	g_3	Activated naïve CD8+ T cell division generations	12		15~20(13)
158	g_4	Activated memory CD8+ T cell division generations	8		

Affinity Maturation

57	m	Increase in affinity during B cell affinity maturation	0.1	10^{-6}mL·day^{-1}	
146	K_{GC}	Carrying Capacity for Germinal center B cells	3	10^{6}/mL	
56	r_{GC}	GC B cell proliferation rate	1	10^{6}ml^{-1}day^{-1}	

Reference

1. Hancioglu B, Swigon D, Clermont G. A dynamical model of human immune response to influenza A virus infection. J Theor Biol. 2007;246(1):70-86.
2. Jenner AL, Aogo RA, Alfonso S, Crowe V, Deng X, Smith AP, et al. COVID-19 virtual patient cohort suggests immune mechanisms driving disease outcomes. PLOS Pathogens. 2021;17(7):e1009753.
3. Goyal A, Cardozo-Ojeda EF, Schiffer JT. Potency and timing of antiviral therapy as determinants of duration of SARS-CoV-2 shedding and intensity of inflammatory response. Sci Adv. 2020;6(47).
4. Bar-On YM, Flamholz A, Phillips R, Milo R. SARS-CoV-2 (COVID-19) by the numbers. eLife. 2020;9:e57309.
5. De Boer RJ, Homann D, Perelson AS. Different Dynamics of CD4⁺ and CD8⁺ T Cell Responses During and After Acute Lymphocytic Choriomeningitis Virus Infection. The Journal of Immunology. 2003;171(8):3928.
6. De Boer Rob J, Oprea M, Antia R, Murali-Krishna K, Ahmed R, Perelson Alan S. Recruitment Times, Proliferation, and Apoptosis Rates during the CD8+ T-Cell Response to Lymphocytic Choriomeningitis Virus. Journal of Virology. 2001;75(22):10663-9.
7. Price I, Mochan-Keef ED, Swigon D, Ermentrout GB, Lukens S, Toapanta FR, et al. The inflammatory response to influenza A virus (H1N1): An experimental and mathematical study. J Theor Biol. 2015;374:83-93.
8. Lahoz-Beneytez J, Elemans M, Zhang Y, Ahmed R, Salam A, Block M, et al. Human neutrophil kinetics: modeling of stable isotope labeling data supports short blood neutrophil half-lives. Blood. 2016;127(26):3431-8.
9. De Boer RJ, Homann D, Perelson AS. Different Dynamics of CD4+ and CD8+ T Cell Responses During and After Acute Lymphocytic Choriomeningitis Virus Infection. The Journal of Immunology. 2003;171(8):3928-35.
10. Dan JM, Mateus J, Kato Y, Hastie KM, Yu ED, Faliti CE, et al. Immunological memory to SARS-CoV-2 assessed for up to 8 months after infection. Science. 2021;371(6529):eabf4063.
11. Bromage E, Stephens R, Hassoun L. The third dimension of ELISPOTs: quantifying antibody secretion from individual plasma cells. J Immunol Methods. 2009;346(1-2):75-9.
12. Andraud M, Lejeune O, Musoro JZ, Ogunjimi B, Beutels P, Hens N. Living on three time scales: the dynamics of plasma cell and antibody populations illustrated for hepatitis a virus. PLoS computational biology. 2012;8(3):e1002418-e.
13. Kaech SM, Wherry EJ, Ahmed R. Effector and memory T-cell differentiation: implications for vaccine development. Nat Rev Immunol. 2002;2(4):251-62.
