# Supplementary material for: Dynamical modelling of viral infection and cooperative immune protection in COVID-19 patients
Source: PLoS Comput Biol. 2023 Sep 1;19(9):e1011383. doi: 10.1371/journal.pcbi.1011383 (PMC10501599; doi:10.1371/journal.pcbi.1011383)
Supplement: S3 Table — (PDF) [file pcbi.1011383.s033.pdf]

Table S3.

Table S3. Variable descriptions and choice of initial parameters.

Epithelial cells, immune cells, cytokines and antibody level is set to be at steady state number density / concentration. Viral load is set to be 1×10^4/mL as a signal for this excitable system.

Variable	Description	Initial Value	Unit	Variable	Description	Initial Value	Unit
nCoV	SARS-CoV-2 viral load	0.01	10^6/mL	$Treg^r$	Resting Treg cell (nTreg)	1	10^6/mL
If	Infected cell	0	10^6/mL	$CD4^+T_M$	Memory SARS-CoV-2-specifc CD4+ T cell	0	10^6/mL
H	Healthy lung epithelial cell	50	10^6/mL	$CD8^+T_N$	Naïve SARS-CoV-2-specific CD8+ T cell	K_{CD8}	10^6/mL
D	Dead infected cell	0	10^6/mL	$CD8^+T_A$	Activated SARS-CoV-2-specific CD8+ T cell	0	10^6/mL
A	Antibody affinity for antigen	0	-	CTL	Cytotoxic T lymphocytes	0	10^6/mL

APC^l	Antigen-loaded Antigen-presenting cell (DC & Mφ)	0	10^6/mL	$CD8^+T_M$	Memory SARS-CoV-2-specifc CD4+ T cell	0	10^6/mL
APC^u	Unloaded antigen-presenting cell (DC & Mφ)	1	10^6/mL	B_{GC}	Germinal center B cell	0	10^6/mL
NK	Natural Killer cell	0	10^6/mL	PB	Plasma B cell	0	10^6/mL
Neut	Neutrophil	0	10^6/mL	B_M	Memory B cell	0	10^6/mL
$CD4^+T_N$	Naïve SARS-CoV-2-specifc CD4+ T cell	K_{CD4}	10^6/mL	IL-2	Cytokine	3	pg/mL
$CD4^+T_A$	Activated SARS-CoV-2-specifc CD4+ T cell	0	10^6/mL	IL-4	Cytokine	30	pg/mL
Th1	T helper 1 cell	0	10^6/mL	IL-6	Cytokine	20	pg/mL
Th2	T helper 2 cell	0	10^6/mL	IL-10/ TGF-β	Cytokine	30	pg/mL

Th17	T helper 17 cell	0	10^6/mL	TNF-α	Cytokine	20	pg/mL
Tfh	Follicular T helper cell	0	10^6/mL	IFN-γ	Cytokine	10	pg/mL
Treg[a]	Activated Treg cell (iTreg & nTreg)	0	10^6/mL	Ab	Antibody	0	μg/mL
