# Supplementary material for: Dynamical modelling of viral infection and cooperative immune protection in COVID-19 patients
Source: PLoS Comput Biol. 2023 Sep 1;19(9):e1011383. doi: 10.1371/journal.pcbi.1011383 (PMC10501599; doi:10.1371/journal.pcbi.1011383)
Supplement: S4 Table — (PDF) [file pcbi.1011383.s034.pdf]

Table S4.

Table S4. Definition of Mode 1, 2, 3, and 4 patients in the main text.

Viral load & cell density: 10^6/mL; Cytokine: pg/mL; Antibody: μg/mL

Var	Mode 1	Mode 2	Mode 3	Mode 4
$[nCoV]$	$[nCoV]_{final} < 10^{-6}$	$[nCoV]_{\max} > 1$ $[nCoV]_{final} < 10^{-6}$	$[nCoV]_{\max} > 1$ $[nCoV]_{final} < 10^{-6}$	$[nCoV] > 1$ After viral load peak
$[IL-6]$	$[IL-6]_{\max} < 1000$	$[IL-6]_{\max} \in [1000,2000]$	$[IL-6]_{\max} > 2000$	$[IL-6]_{\max} > 2000$
Physiological Range				
	$[IL-6]_{\max} < 50000$			
$[APC]$	$[APC]_{\max} < 5$			
	$[NK]_{\max} < 0.5$			
$[Neut]$	$[Neut]_{\max} < 5$			
Lymphocytes	$[CD4+T]_{\max} + [CD8+T]_{\max} < 8$			
Cytokines	$[IL-2]_{\max} < 500$, $[IL-4]_{\max} < 200$, $[IL-6]_{\max} < 50000[IL-10]_{\max} < 2000$, $[TNF-\alpha]_{\max} < 1000$, $[IFN-\gamma]_{\max} < 500$			
