# Supplementary material for: Dynamical modelling of viral infection and cooperative immune protection in COVID-19 patients
Source: PLoS Comput Biol. 2023 Sep 1;19(9):e1011383. doi: 10.1371/journal.pcbi.1011383 (PMC10501599; doi:10.1371/journal.pcbi.1011383)
Supplement: S5 Table — (PDF) [file pcbi.1011383.s035.pdf]

Table S5.

Table S5. The geometric mean parameter sets of Mode 1~4.

index	parameter	Mode 1	Mode 2	Mode 3	Mode 4	unit
1	k_{nCoV}^{APC}	1.323E-01	1.095E-01	1.005E-01	1.019E-01	day^{-1}
2	k_{If}^{nCoV}	8.842E-02	1.071E-01	1.091E-01	1.017E-01	day^{-1}
3	k_{rcr}^{APC}	1.346E-01	1.159E-01	9.355E-02	2.530E-02	day^{-1}
4	k_{If}^{NK}	4.909E-03	5.396E-03	3.879E-03	3.957E-03	day^{-1}
5	k_{APC}^{NK}	5.983E-03	4.868E-03	4.241E-03	4.931E-03	day^{-1}
6	k_{If}^{Neut}	1.122E-01	9.025E-02	9.470E-02	8.019E-02	day^{-1}
7	k_{D}^{Neut}	8.904E-02	9.773E-02	1.035E-01	1.061E-01	day^{-1}
8	k_{Th17}^{Neut}	5.089E-02	4.916E-02	5.047E-02	4.899E-02	day^{-1}
9	k_{naive}^{CD4}	3.051E-02	2.960E-02	3.102E-02	3.023E-02	
10	k_{mem}^{CD4}	6.049E-02	5.994E-02	5.875E-02	6.175E-02	
11	k_{CD4}^{mem}	2.000E-02	2.000E-02	2.000E-02	2.000E-02	
12	k_{CD4}^{Th1}	4.063E-01	3.933E-01	3.983E-01	4.148E-01	day^{-1}
13	k_{CD4}^{Th2}	1.004E-01	1.003E-01	1.085E-01	9.676E-02	day^{-1}
14	k_{CD4}^{Th17}	9.906E-02	9.982E-02	1.002E-01	9.936E-02	day^{-1}
15	k_{CD4}^{Tfh}	1.995E-01	2.037E-01	2.123E-01	1.892E-01	day^{-1}
16	k_{CD4}^{iTreg}	1.010E-01	9.761E-02	1.002E-01	9.479E-02	day^{-1}
17	k_{APC}^{nTreg}	2.975E-01	2.984E-01	2.998E-01	3.698E-01	day^{-1}
18	k_{naive}^{CD8}	4.068E-02	3.799E-02	3.719E-02	3.658E-02	
19	k_{mem}^{CD8}	6.030E-02	5.767E-02	6.128E-02	6.076E-02	
20	k_{CD8}^{CTL}	4.149E-01	4.041E-01	4.147E-01	4.207E-01	day^{-1}
21	k_{CD8}^{mem}	4.000E-02	4.000E-02	4.000E-02	4.000E-02	
22	k_{naive}^{GC}	3.117E-01	2.989E-01	3.071E-01	3.049E-01	

23	k_{mem}^{GC}	4.887E-01	5.086E-01	5.042E-01	4.733E-01	
24	k_{PB}	4.821E-02	5.008E-02	5.042E-02	4.612E-02	
25	k_{Bm}	2.000E-02	2.000E-02	2.000E-02	2.000E-02	
26	k_{infect}	1.200E-04	1.200E-04	1.200E-04	1.200E-04	10^{-6}mL·day^{-1}
27	d_v	8.000E-03	8.000E-03	8.000E-03	8.000E-03	
28	k_1^{clear}	6.752E-01	3.753E-01	2.396E-01	3.564E-01	10^{-6}mL·day^{-1}
29	k_2^{clear}	4.725E-01	4.545E-01	3.546E-01	4.050E-01	μg^{-1}·day^{-1}
30	k_3^{clear}	8.195E-01	4.833E-01	3.044E-01	3.229E-01	10^{-6}mL·day^{-1}
31	k_4^{clear}	6.630E-03	6.000E-03	6.225E-03	6.069E-03	mL·μg^{-1}·day^{-1}
32	k_1^{kill}	5.925E-01	4.273E-01	2.523E-01	3.221E-01	10^{-6}mL·day^{-1}
33	k_2^{kill}	2.219E-01	2.210E-01	2.226E-01	2.165E-01	10^{-6}mL·day^{-1}
34	k_3^{kill}	2.395E+00	2.161E+00	1.820E+00	1.514E+00	10^{-6}mL·day^{-1}
35	k_4^{kill}	1.622E+00	1.538E+00	1.483E+00	1.432E+00	10^{-6}mL·day^{-1}
36	k_5^{kill}	1.028E+00	9.822E-01	1.009E+00	1.021E+00	10^{-6}mL·day^{-1}
37	h_D^{APC}	3.887E+00	4.009E+00	4.327E+00	3.815E+00	1
38	$h_{TNF-\alpha}^{APC}$	1.751E+00	2.071E+00	2.124E+00	2.026E+00	1
39	$h_{IFN-\gamma}^{APC}$	2.055E+00	1.950E+00	1.954E+00	1.881E+00	1
40	h_{IL-2}^{NK}	5.035E-01	5.068E-01	4.859E-01	5.078E-01	

41	h_{IL-2}^{CD4}	5.004E-01	4.845E-01	5.174E-01	5.418E-01	
42	$h_{IFN-\gamma}^{Th1}$	1.927E+00	1.934E+00	2.006E+00	1.895E+00	1
43	h_{IL-4}^{Th2}	2.046E+00	1.998E+00	1.941E+00	2.067E+00	1
44	h_{IL-6}^{Th17}	1.042E+00	1.002E+00	9.844E-01	1.023E+00	1
45	h_{Neut}^{Th17}	5.116E-01	5.073E-01	5.164E-01	4.791E-01	1
46	h_{B}^{Tfh}	2.083E+00	1.955E+00	1.991E+00	1.910E+00	
47	h_{IL-10}^{iTreg}	2.043E+00	1.963E+00	1.940E+00	1.973E+00	1
48	h_{IL-2}^{CD8}	4.895E-01	4.882E-01	5.037E-01	4.985E-01	
49	h_{Th1}^{CTL}	3.052E+00	3.033E+00	2.881E+00	3.110E+00	1
50	h_{IL-2}^{CTL}	1.020E+00	9.940E-01	1.012E+00	1.022E+00	1
51	h_{IL-6}^{CTL}	9.838E-01	1.013E+00	1.032E+00	9.238E-01	1
52	h_{IL-4}^{Ab}	1.977E-01	1.974E-01	1.973E-01	1.921E-01	1
53	r_{H}	2.000E+00	2.000E+00	2.000E+00	2.000E+00	$10^6 ml^{-1} day^{-1}$
54	r_{APC}	4.000E-03	4.000E-03	4.000E-03	4.000E-03	$10^6 ml^{-1} day^{-1}$
55	r_{Treg^r}	1.000E-03	1.000E-03	1.000E-03	1.000E-03	$10^6 ml^{-1} day^{-1}$
56	r_{GC}	1.000E+00	1.000E+00	1.000E+00	1.000E+00	
57	m	1.000E-01	1.000E-01	1.000E-01	1.000E-01	$10^{-6} mL\cdot day^{-1}$
58	d_{Treg}^{APC}	4.000E-01	4.000E-01	4.000E-01	4.000E-01	$10^{-6} mL\cdot day^{-1}$
59	d_{Treg}^{NK}	4.000E-01	4.000E-01	4.000E-01	4.000E-01	$10^{-6} mL\cdot day^{-1}$
60	d_{Treg}^{CD4}	4.000E-01	4.000E-01	4.000E-01	4.000E-01	$10^{-6} mL\cdot day^{-1}$

61	d_{Treg}^{CD8}	4.000E-01	4.000E-01	4.000E-01	4.000E-01	10^{-6}mL·day^{-1}
62	d_{If}	4.000E-01	4.000E-01	4.000E-01	4.000E-01	day^{-1}
63	d_{H}	4.000E-02	4.000E-02	4.000E-02	4.000E-02	day^{-1}
64	d_{D}	5.000E-02	5.000E-02	5.000E-02	5.000E-02	day^{-1}
65	$d_{APC^{l}}$	4.000E-01	4.000E-01	4.000E-01	4.000E-01	day^{-1}
66	$d_{APC^{u}}$	2.000E-01	2.000E-01	2.000E-01	2.000E-01	day^{-1}
67	d_{NK}	6.000E-01	6.000E-01	6.000E-01	6.000E-01	day^{-1}
68	d_{Neut}	1.600E+00	1.600E+00	1.600E+00	1.600E+00	day^{-1}
69	d_{Th}	8.000E-01	8.000E-01	8.000E-01	8.000E-01	day^{-1}
70	$d_{Treg^{a}}$	4.000E-01	4.000E-01	4.000E-01	4.000E-01	day^{-1}
71	$d_{Treg^{r}}$	5.000E-02	5.000E-02	5.000E-02	5.000E-02	day^{-1}
72	d_{CD4Tm}	2.000E-03	2.000E-03	2.000E-03	2.000E-03	day^{-1}
73	d_{CTL}	8.000E-01	8.000E-01	8.000E-01	8.000E-01	day^{-1}
74	d_{CD8Tm}	1.500E-03	1.500E-03	1.500E-03	1.500E-03	day^{-1}
75	d_{GC}	2.000E-01	2.000E-01	2.000E-01	2.000E-01	day^{-1}
76	d_{PB}	4.000E-01	4.000E-01	4.000E-01	4.000E-01	day^{-1}
77	d_{Bm}	3.000E-05	3.000E-05	3.000E-05	3.000E-05	day^{-1}
78	p_{0}^{IL-2}	3.000E+01	3.000E+01	3.000E+01	3.000E+01	pg·mL^{-1}day^{-1}
79	p_{1}^{IL-2}	8.000E+01	8.000E+01	8.000E+01	8.000E+01	10^{-6}pg·day^{-1}
80	p_{2}^{IL-2}	8.000E+01	8.000E+01	8.000E+01	8.000E+01	10^{-6}pg·day^{-1}
81	p_{3}^{IL-2}	6.000E+01	6.000E+01	6.000E+01	6.000E+01	10^{-6}pg·day^{-1}
82	p_{4}^{IL-2}	4.000E+01	4.000E+01	4.000E+01	4.000E+01	10^{-6}pg·day^{-}

						1
83	p_0^{IL-4}	1.000E+02	1.000E+02	1.000E+02	1.000E+02	pg·mL^{-1}day^{-1}
84	p_1^{IL-4}	2.000E+03	2.000E+03	2.000E+03	2.000E+03	10^{-6}pg·day^{-1}
85	p_0^{IL-6}	2.000E+02	2.000E+02	2.000E+02	2.000E+02	pg·mL^{-1}day^{-1}
86	p_1^{IL-6}	1.000E+03	1.000E+03	1.000E+03	1.000E+03	10^{-6}pg·day^{-1}
87	p_2^{IL-6}	1.500E+03	1.500E+03	1.500E+03	1.500E+03	10^{-6}pg·day^{-1}
88	p_3^{IL-6}	3.000E+03	3.000E+03	3.000E+03	3.000E+03	10^{-6}pg·day^{-1}
89	p_0^{IL-10}	2.000E+02	2.000E+02	2.000E+02	2.000E+02	pg·mL^{-1}day^{-1}
90	p_1^{IL-10}	4.000E+03	4.000E+03	4.000E+03	4.000E+03	10^{-6}pg·day^{-1}
91	p_2^{IL-10}	1.000E+03	1.000E+03	1.000E+03	1.000E+03	10^{-6}pg·day^{-1}
92	$p_0^{TNF-\alpha}$	2.000E+02	2.000E+02	2.000E+02	2.000E+02	pg·mL^{-1}day^{-1}
93	$p_1^{TNF-\alpha}$	4.000E+01	4.000E+01	4.000E+01	4.000E+01	10^{-6}pg·day^{-1}
94	$p_2^{TNF-\alpha}$	4.000E+02	4.000E+02	4.000E+02	4.000E+02	10^{-6}pg·day^{-1}
95	$p_3^{TNF-\alpha}$	2.000E+02	2.000E+02	2.000E+02	2.000E+02	10^{-6}pg·day^{-1}

96	$p_0^{IFN-\gamma}$	1.000E+02	1.000E+02	1.000E+02	1.000E+02	$pg\cdot mL^{-1}day^{-1}$
97	$p_1^{IFN-\gamma}$	2.000E+02	2.000E+02	2.000E+02	2.000E+02	$10^{-6}pg\cdot day^{-1}$
98	$p_2^{IFN-\gamma}$	1.000E+02	1.000E+02	1.000E+02	1.000E+02	$10^{-6}pg\cdot day^{-1}$
99	$p_3^{IFN-\gamma}$	1.000E+02	1.000E+02	1.000E+02	1.000E+02	$10^{-6}pg\cdot day^{-1}$
100	p_1^{Ab}	2.500E+02	2.500E+02	2.500E+02	2.500E+02	$10^{-6}\mu g\cdot day^{-1}$
101	p_2^{Ab}	1.500E+02	1.500E+02	1.500E+02	1.500E+02	$10^{-6}\mu g\cdot day^{-1}$
102	c_{IL-2}	1.000E+01	1.000E+01	1.000E+01	1.000E+01	day^{-1}
103	c_{IL-4}	1.000E+01	1.000E+01	1.000E+01	1.000E+01	day^{-1}
104	c_{IL-6}	1.000E+01	1.000E+01	1.000E+01	1.000E+01	day^{-1}
105	c_{IL-10}	1.000E+01	1.000E+01	1.000E+01	1.000E+01	day^{-1}
106	$c_{TNF-\alpha}$	1.000E+01	1.000E+01	1.000E+01	1.000E+01	day^{-1}
107	$c_{IFN-\gamma}$	1.000E+01	1.000E+01	1.000E+01	1.000E+01	day^{-1}
108	c_{Ab}	4.000E-02	4.000E-02	4.000E-02	4.000E-02	day^{-1}
109	K_1^{IL-2}	8.000E+01	8.000E+01	8.000E+01	8.000E+01	pg/mL
110	K_2^{IL-2}	8.000E+01	8.000E+01	8.000E+01	8.000E+01	pg/mL
111	K_3^{IL-2}	4.000E+01	4.000E+01	4.000E+01	4.000E+01	pg/mL
112	K_4^{IL-2}	4.000E+01	4.000E+01	4.000E+01	4.000E+01	pg/mL
113	K_5^{IL-2}	8.000E+01	8.000E+01	8.000E+01	8.000E+01	
114	K_6^{IL-2}	8.000E+01	8.000E+01	8.000E+01	8.000E+01	
115	K_1^{IL-4}	3.000E+01	3.000E+01	3.000E+01	3.000E+01	pg/mL
116	K_2^{IL-4}	3.000E+01	3.000E+01	3.000E+01	3.000E+01	pg/mL

117	K_3^{IL-4}	3.000E+01	3.000E+01	3.000E+01	3.000E+01	pg/mL
118	K_1^{IL-6}	1.500E+03	1.500E+03	1.500E+03	1.500E+03	pg/mL
119	K_2^{IL-6}	1.500E+03	1.500E+03	1.500E+03	1.500E+03	pg/mL
120	K_3^{IL-6}	1.500E+03	1.500E+03	1.500E+03	1.500E+03	pg/mL
121	K_1^{IL-10}	1.000E+02	1.000E+02	1.000E+02	1.000E+02	pg/mL
122	K_2^{IL-10}	8.000E+01	8.000E+01	8.000E+01	8.000E+01	pg/mL
123	K_3^{IL-10}	8.000E+01	8.000E+01	8.000E+01	8.000E+01	pg/mL
124	K_4^{IL-10}	1.000E+02	1.000E+02	1.000E+02	1.000E+02	pg/mL
125	$K_1^{TNF-\alpha}$	1.000E+02	1.000E+02	1.000E+02	1.000E+02	pg/mL
126	$K_1^{IFN-\gamma}$	8.000E+01	8.000E+01	8.000E+01	8.000E+01	pg/mL
127	$K_2^{IFN-\gamma}$	8.000E+01	8.000E+01	8.000E+01	8.000E+01	pg/mL
128	$K_3^{IFN-\gamma}$	8.000E+01	8.000E+01	8.000E+01	8.000E+01	pg/mL
129	K_{ACD4}	1.786E-01	2.149E-01	1.842E-01	2.005E-01	10^6/mL
130	K_{ACD8}	1.931E-01	2.032E-01	2.589E-01	3.996E-01	10^6/mL
131	K_{AB}	3.710E-01	3.811E-01	3.406E-01	6.513E-01	10^6/mL
132	K_{mem}	1.704E-01	1.881E-01	1.763E-01	1.150E-01	10^6/mL
133	K_m	1.000E-03	1.000E-03	1.000E-03	1.000E-03	
134	K_1^{If}	1.000E-01	1.000E-01	1.000E-01	1.000E-01	10^6/mL
135	K_2^{If}	1.000E-01	1.000E-01	1.000E-01	1.000E-01	10^6/mL
136	K_3^{If}	1.000E-01	1.000E-01	1.000E-01	1.000E-01	10^6/mL
137	K_1^{D}	2.000E+01	2.000E+01	2.000E+01	2.000E+01	10^6/mL
138	K_2^{D}	2.000E+01	2.000E+01	2.000E+01	2.000E+01	10^6/mL
139	K_1^{APC}	2.000E-01	2.000E-01	2.000E-01	2.000E-01	10^6/mL
140	K_2^{APC}	2.000E-01	2.000E-01	2.000E-01	2.000E-01	10^6/mL
141	K_1^{Neut}	6.000E-01	6.000E-01	6.000E-01	6.000E-01	10^6/mL
142	K_1^{Th1}	1.000E-01	1.000E-01	1.000E-01	1.000E-01	10^6/mL
143	K_1^{Th17}	5.000E-02	5.000E-02	5.000E-02	5.000E-02	10^6/mL

144	K_1^{Tfh}	2.000E-02	2.000E-02	2.000E-02	2.000E-02	10^6/mL
145	K_1^{GC}	5.000E-01	5.000E-01	5.000E-01	5.000E-01	10^6/mL
146	K_{GC}	5.000E+00	5.000E+00	5.000E+00	5.000E+00	
147	APC_0	5.000E+00	5.000E+00	5.000E+00	5.000E+00	10^6/mL
148	NK_0	5.000E+00	5.000E+00	5.000E+00	5.000E+00	10^6/mL
149	$Neut_0$	1.000E+01	1.000E+01	1.000E+01	1.000E+01	10^6/mL
150	B_0	1.199E-02	1.089E-02	1.052E-02	7.832E-03	10^6/mL
151	N_I	1.500E+03	1.500E+03	1.500E+03	1.500E+03	1
152	N_{ex}	1.000E+01	1.000E+01	1.000E+01	1.000E+01	1
153	t_{CD4}	4.167E-01	4.167E-01	4.167E-01	4.167E-01	day
154	t_{CD8}	2.500E-01	2.500E-01	2.500E-01	2.500E-01	day
155	g_1	1.100E+01	1.100E+01	1.100E+01	1.100E+01	
156	g_2	6.000E+00	6.000E+00	6.000E+00	6.000E+00	
157	g_3	1.200E+01	1.200E+01	1.200E+01	1.200E+01	
158	g_4	8.000E+00	8.000E+00	8.000E+00	8.000E+00	
159	$CD4^{+}T_n$	1.892E-02	2.090E-02	2.077E-02	1.824E-02	10^6/mL
160	$CD8^{+}T_n$	1.058E-02	8.273E-03	6.958E-03	6.650E-03	10^6/mL
