# Supplementary material for: Dynamical modelling of viral infection and cooperative immune protection in COVID-19 patients
Source: PLoS Comput Biol. 2023 Sep 1;19(9):e1011383. doi: 10.1371/journal.pcbi.1011383 (PMC10501599; doi:10.1371/journal.pcbi.1011383)
Supplement: S6 Table — (PDF) [file pcbi.1011383.s036.pdf]

Table S6.

Table S6. The sources of vaccine immunogenicity data and efficacies for different variants.

Vaccine	Reference of Immunogenicity Data		Figure	Strain	Reference of Efficacy
	Antibody	T cell			
CoronaVac (Sinovac)	From Sinovac	From Sinovac (Elispot)	Figure 3F, Figure S23B	SARS-CoV-2	[62]
ChAdOx1 nCoV-19 (AstraZeneca/University of Oxford)	Fig.4B, Day 35 [63]	Fig.6 right, Day 35 [63] (Elispot)	Figure S23C	SARS-CoV-2	[64]
				B.1.1.7 (Alpha)	[65]
				B.1.617.2 (Delta)	[65]
				B.1.1.529 (Omicron)	[66]
BNT162b2 (Pfizer/BioNTech)	Fig.1a, 30 μg, Day 29 [67]	Fig.2a, bottom right, 30 μg [67] (Elispot)	Figure 3F	SARS-CoV-2	[68]
			Figure S23C	B.1.1.7 (Alpha)	[65]
				B.1.617.2 (Delta)	[65]
				B.1.1.529 (Omicron)	[66]

		Fig.1B infection naïve (two dose) S2 [69] (Elispot)	Figure S23B	SARS-CoV-2	[68]
		Fig.3b, bottom left, Day 29 [67] (Flow Cytometry)	Figure S23A	SARS-CoV-2	[68]
BNT162b2 (one dose)	Fig.1a, 30ug, Day 22 [67]	Fig1B infection naïve (one dose) S2 [69]	Figure S23B	SARS-CoV-2	[70]
mRNA-1273 (Moderna)	Fig. 2D, 100ug, Day 43 [71]	SI Figure S11B S1 Pool, Day 43, Any Response (Flow Cytometry) [71]	Figure S23A	SARS-CoV-2	[72]
Johnson & Johnson	Fig. 2B, low dose / placebo group, Day 29 [73]	Fig. 3C Low dose, Day 15 [73](Flow Cytometry)	Figure S23A	SARS-CoV-2	From press release
