# Supplementary material for: Dynamical modelling of viral infection and cooperative immune protection in COVID-19 patients
Source: PLoS Comput Biol. 2023 Sep 1;19(9):e1011383. doi: 10.1371/journal.pcbi.1011383 (PMC10501599; doi:10.1371/journal.pcbi.1011383)
Supplement: S7 Table — (PDF) [file pcbi.1011383.s037.pdf]

Table S7.

Table S7. Choice of parameters of original strain (nCoV), Alpha and Delta variants viral infection and immune response.

Parameter	nCoV (original strain)	Alpha Variant (B.1.1.7)	Delta Variant (B.1.617.2)
k_{infect}(10^{-6}mL·day^{-1})	1.2×10^{-4}	1.56×10^{-4}	1.92×10^{-4}
d_{If} (day^{-1})	0.4	0.4	0.4
N_1	1500[54]	1500[54]	1800[54]
$[H]_0$	50	65	75
Others	See Table S2	See Table S2	See Table S2
