# Supplementary material for: Dynamical modelling of viral infection and cooperative immune protection in COVID-19 patients
Source: PLoS Comput Biol. 2023 Sep 1;19(9):e1011383. doi: 10.1371/journal.pcbi.1011383 (PMC10501599; doi:10.1371/journal.pcbi.1011383)
Supplement: S8 Table — SARS-CoV-1 induces stronger innate immunity and higher level of inflammation. (PDF) [file pcbi.1011383.s038.pdf]

Table S8.

Table S8. Change of parameters for modelling SARS-CoV-1 infection and Influenza A Virus infection.

SARS-CoV-1 induces stronger innate immunity and higher level of inflammation,

Virus	Parameter	Change
SARS-CoV-1	k_{nCoV}^{APC}, k_{If}^{APC}, k_{If}^{Neut}, k_{D}^{Neut}, k_{Th17}^{Neut}	×10
	k_{naive}^{CD4}, k_{naive}^{CD8}, k_{naive}^{GC}	×1/2
Influenza A Virus (IAV)	r_H	×0.7
	k_{nCoV}^{APC}, k_{If}^{APC}	×1.5
