# Supplementary material for: Dynamical modelling of viral infection and cooperative immune protection in COVID-19 patients
Source: PLoS Comput Biol. 2023 Sep 1;19(9):e1011383. doi: 10.1371/journal.pcbi.1011383 (PMC10501599; doi:10.1371/journal.pcbi.1011383)
Supplement: S9 Table — (PDF) [file pcbi.1011383.s039.pdf]

Table S9.

Table S9. The change on the model made by various drugs and parameters.

Drug	Effect	Parameter
Antiviral durgs (AntV)	Suppressing viral replication in infected cells	$\alpha = 0.8$
IFN-I	Blocking virus infecting healthy epithelial cells<br>Augmenting the antigen-presentation ability of APC and effector function of NK	$[IFN - I] = K_{IFN-I}/20$<br>$K_{IFN-I} = 14532\ pg/mL$<br>$h_{IFN-I}^{APC} = 2$<br>$h_{IFN-I}^{NK} = 2$
Ab	Increasing Ab concentration	$Ab_{ex} = 400\mu g/mL$<br>$k_5^{clear} = 0.006\ \mu g^{-1}/day$
Glucocorticoids (GC)	Decreasing the cytokine production of APC and inducing activated lymphocyte apoptosis	$\beta = 0.6$<br>$d_{GC}^{lymph} = 0.1\ day^{-1}$
